# Supplementary material for: Cognitive function and treatment response trajectories in first-episode schizophrenia: evidence from a prospective cohort study
Source: BMJ Open. 2022 Nov 21;12(11):e062570. doi: 10.1136/bmjopen-2022-062570 (PMC9680154; doi:10.1136/bmjopen-2022-062570)
Supplement: Supplementary data [file bmjopen-2022-062570supp001.pdf]

Supplementary material

Table S.1

Results from univariable and multivariable logistic regression models for response status using PANSS total >20% reduction criteria and baseline BACS performance

	Unadjusted					Adjusted for age, gender and DUP				
BACS task	β	SE	95%CI	OR	P-value	β	SE	95%CI	OR	P-value
Verbal Memory	-0.01	0.03	-0.07 ; 0.06	0.99	.819	<-0.01	0.04	-0.08 ; 0.07	1.00	.920
Digit Sequencing	-0.10	0.10	-0.29 ; 0.09	0.90	.297	-0.06	0.11	-0.27 ; 0.16	0.95	.604
Verbal Fluency	0.04	0.05	-0.06 ; 0.14	1.04	.403	0.07	0.06	-0.05 ; 0.18	1.07	.259
Token Motor	0.03	0.04	-0.04 ; 0.10	1.03	.432	0.06	0.05	-0.03 ; 0.15	1.06	.218
Symbol Coding	-0.04	0.03	-0.11 ; 0.02	0.96	.206	-0.05	0.04	-0.12 ; 0.03	0.96	.237
Tower of London	0.07	0.09	-0.11 ; 0.24	1.07	.445	0.07	0.09	-0.12 ; 0.25	1.07	.465
t score composite	<0.01	0.03	-0.06 ; 0.07	1.00	.924	0.01	0.03	-0.06 ; 0.07	1.00	.892
z score composite	0.02	0.33	-0.63 ; 0.66	1.02	.960	0.03	0.34	-0.63 ; 0.69	1.03	.931

Note. *BACS* = Brief Assessment of Cognition in Schizophrenia; *DUP* = duration of untreated psychosis; *CIs* = confidence intervals.

Table S.2

Results from unadjusted and adjusted growth curve models comparing trajectory groups on BACS performance across all visits

	Unadjusted				Adjusted for age, gender and DUP			
BACS task	β	SE	95%CI	P-value	β	SE	95%CI	P-value
Verbal Memory	-2.15	45.79	-13. 49 ; 9.19	.710	-2.67	5.88	-14.20 ;8.86	.650
Digit Sequencing	-2.25	1.63	-5.45 ; 1 0.95	.167	-2.03	1.62	-5.19 ; 1.14	.210
Verbal Fluency	-0.90	3.29	-7.36 ; 5.55	.784	-1.31	2.99	-7.17 ; 4.54	.660
Token Motor	-2.00	6.39	-14.52 ; 10.53	.755	-0.45	5.89	11.99 ; 11.10	.939
Symbol Coding	-3.81	5.56	-14.71 ; 7.08	.493	-4.25	5.42	-14.87 ; 6.37	.433
Tower of London	0.18	1.56	-2.87 ; 3.24	.906	0.17	1.57	-2.90 ; 3.25	.912
t score composite	-2.49	5.70	-13.66 ; 8.68	.662	-2.43	5.78	-13.76 ; 8.89	.674
z score composite	-0.25	0.57	-1.37 ; 0.87	.658	-0.25	0.58	-1.38 ; 0.88	.665

Note. BACS = Brief Assessment of Cognition in Schizophrenia; *DUP* = duration of untreated psychosis; *CIs* = confidence intervals

Table S.3

Results from unadjusted and adjusted growth curve models comparing PANSS >20% reduction groups on BACS performance across all visits

	Unadjusted				Adjusted for age, gender and DUP			
BACS task	β	SE	95%CI	P-value	β	SE	95%CI	P-value
Verbal Memory	-4.18	4.21	-12.44 ; 4.08	.321	3.27	4.34	-11.78 ; 5.23	.451
Digit Sequencing	-2.36	1.35	-5.01 ; 0.29	.081	-1.78	1.34	-4.41 ; 0.86	.186
Verbal Fluency	-0.97	2.55	-5.97 ; 4.02	.703	-0.54	2.49	-5.42 ; 4.35	.830
Token Motor	1.81	5.52	-9.01 ; 12.62	.744	5.60	5.07	-4.33 ; 15.53	.269
Symbol Coding	-4.23	3.92	-11.92 ; 3.46	.281	-4.13	4.03	-12.03 ; 3.78	.306
Tower of London	-0.77	1.25	-3.22 ; 1.68	.538	-0.56	1.30	-3.10 ; 1.98	.667
t score composite	-2.39	4.75	-11.71 ; 6.93	.615	-1.56	4.84	-11.03 ; 7.92	.747
z score composite	-0.25	0.48	-1.18 ; 0.68	.600	-0.17	0.48	-1.12 ; 0.78	.728

Note. BACS = Brief Assessment of Cognition in Schizophrenia; *DUP* = duration of untreated psychosis; *CIs* = confidence intervals

Table S.4

Descriptive statistics of clinical and demographic variables for each trajectory group at baseline assessments

	Non-responder			Responder		
Variable	N	M	SD	N	M	SD
Age (at consent)	6	29.57	6.70	39	26.90	8.41
Gender (*male*)	6	-	-	27	-	-
Gender (*female*)	1	-	-	12	-	-
Age of illness onset (years)	7	27.54	8.09	39	26.34	8.60
Duration from 1st Psychotic symptom (days) to baseline antipsychotic (DUP)	7	177.09	207.93	39	261.08	251.38
Duration from 1st contact with mental health services (days) to baseline antipsychotic	7	461.89	801.88	39	325.88	567.82
Chlorpromazine equivalents (mg/day)	7	271.43	249.76	39	159.92	75.02
Number of hospitalisations	7	1.00	1.00	39	0.87	0.57
Years of education	6	17.00	2.53	36	13.06	2.47
PANSS positive	7	11.14	6.67	38	12.08	4.44
PANSS negative	7	12.57	5.77	38	9.95	7.04
PANSS general	7	19.57	8.70	38	19.37	8.66
PANSS total	7	43.29	13.52	38	41.39	16.68

Note. *PANSS* = Positive and Negative Symptom Scale.

Table S.5

Baseline cognitive performance for both groups using >20% PANSS reduction criteria

	Non-responder			Responder		
BACS measure	N	Mean	SD	N	Mean	SD
Verbal Memory	13	37.54	8.27	20	36.65	12.73
Digit Sequencing	12	19.25	4.20	19	17.68	3.97
Verbal Fluency	14	27.36	6.44	20	29.45	7.75
Token Motor	13	63.23	10.94	18	66.39	11.24
Symbol Coding	12	44.83	9.47	20	39.35	12.75
Tower of London	12	14.17	5.11	17	15.41	3.79
tscore composite	10	26.30	13.35	17	26.77	12.28
zscore composite	10	-2.36	1.31	17	-2.34	1.22

Note. BACS = Brief Assessment of Cognition in Schizophrenia; *PANSS* = Positive and Negative Symptom Scale.

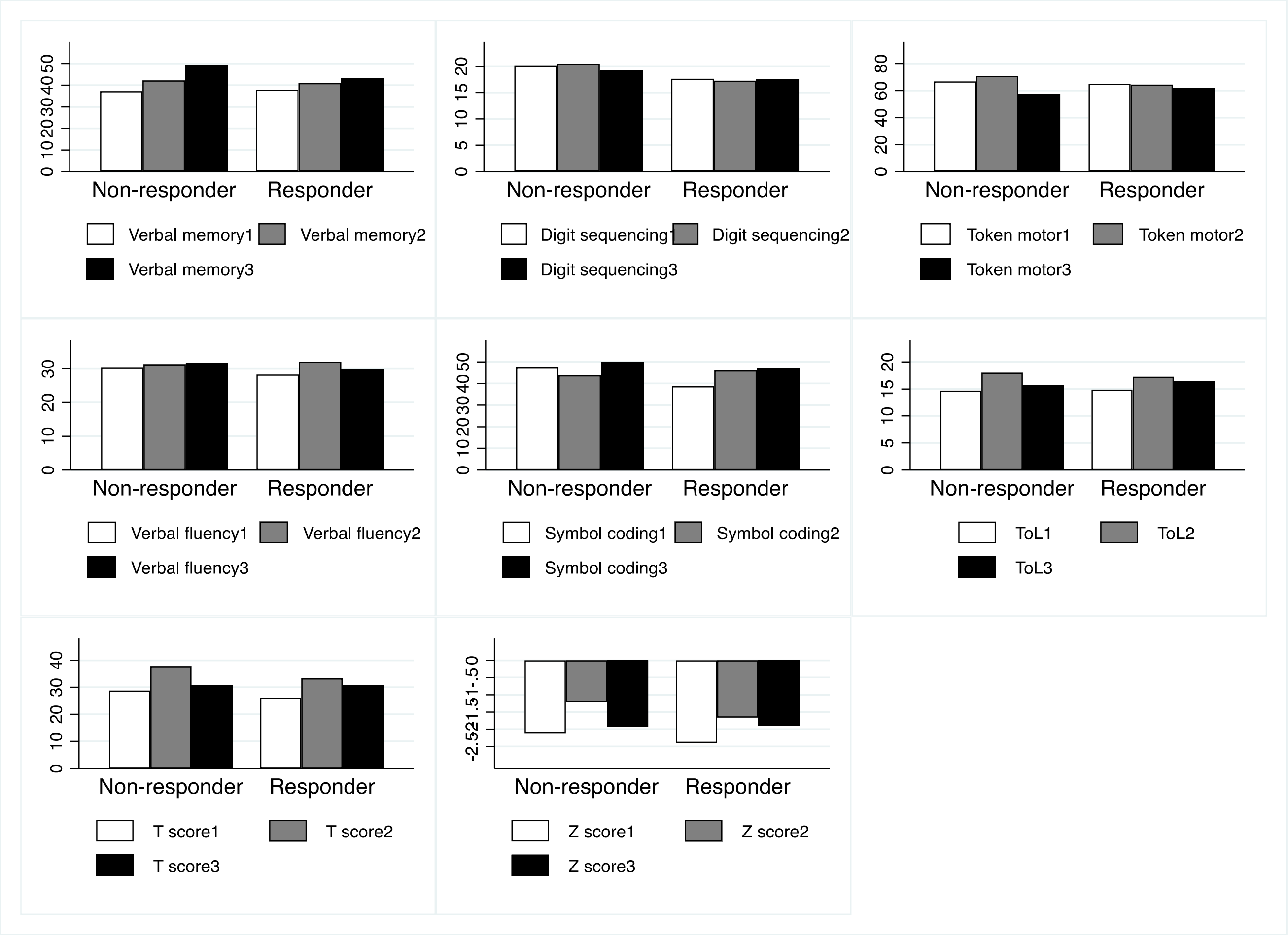

Figure S.1

Bar graphs comparing mean performance on BACS measures between trajectory groups (non-responder vs. responder) at each visit (*white* = baseline*, grey* = 2-week, *black* = 6-week)

Note. *BACS* = Brief Assessment of Cognition in Schizophrenia; *ToL* = Tower of London.

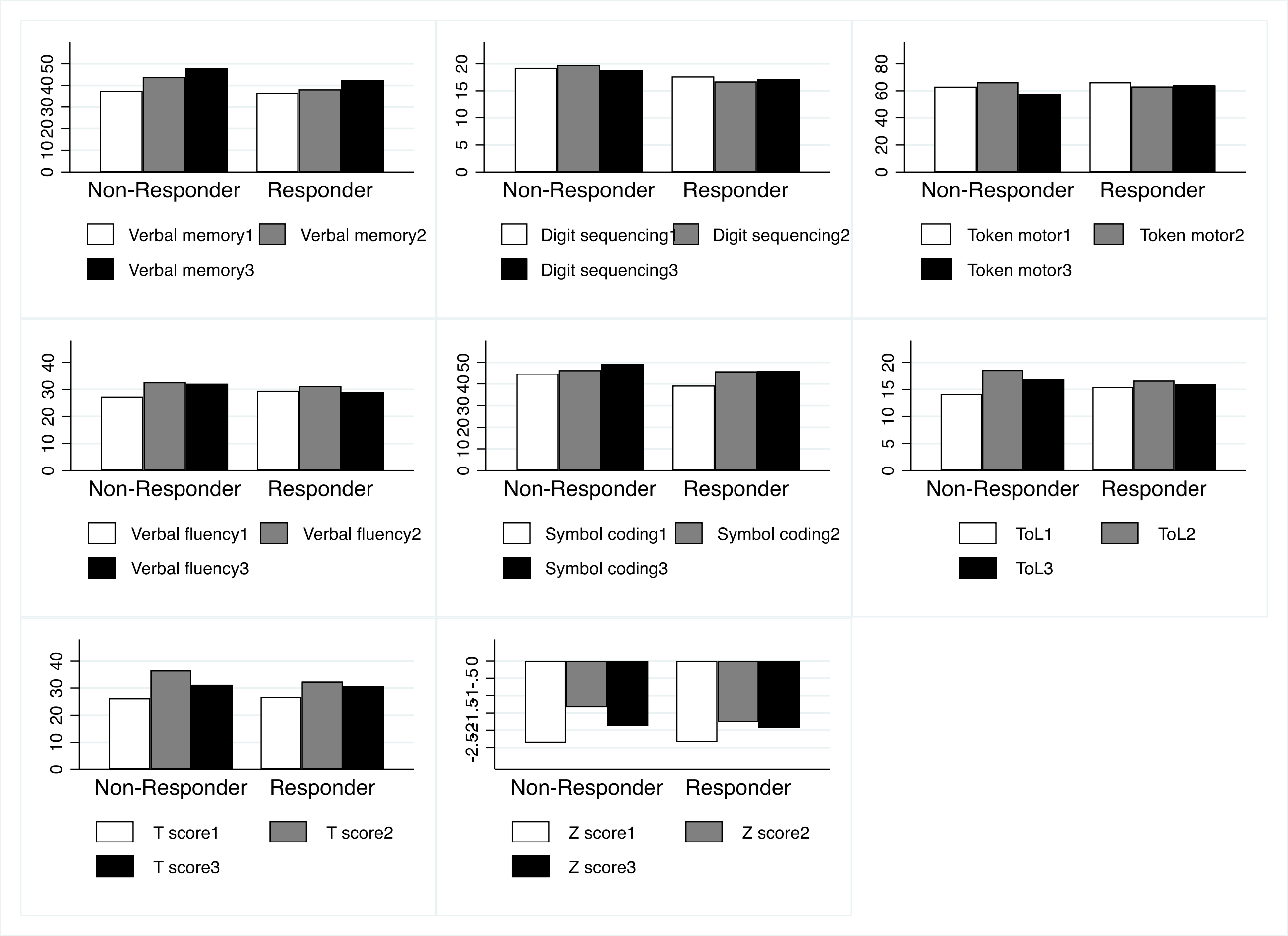

Figure S.2

Bar graphs comparing mean performance on BACS measures using >20% PANSS reduction criteria (non-responder vs. responder) at each visit (*white* = baseline, *grey* = 2-week, *black* = 6-week)

Note. *BACS* = Brief Assessment of Cognition in Schizophrenia; *ToL* = Tower of London.
