# Supplementary material for: Cognitive function and treatment response trajectories in first-episode schizophrenia: evidence from a prospective cohort study
Source: BMJ Open. 2022 Nov 21;12(11):e062570. doi: 10.1136/bmjopen-2022-062570 (PMC9680154; doi:10.1136/bmjopen-2022-062570)
Supplement: Author's manuscript [file bmjopen-2022-062570.draft_revisions.pdf]

BMJ Open

BMJ Open is committed to open peer review. As part of this commitment we make the peer review history of every article we publish publicly available.

When an article is published we post the peer reviewers' comments and the authors' responses online. We also post the versions of the paper that were used during peer review. These are the versions that the peer review comments apply to.

The versions of the paper that follow are the versions that were submitted during the peer review process. They are not the versions of record or the final published versions. They should not be cited or distributed as the published version of this manuscript.

BMJ Open is an open access journal and the full, final, typeset and author-corrected version of record of the manuscript is available on our site with no access controls, subscription charges or pay-per-view fees (http://bmjopen.bmj.com).

If you have any questions on BMJ Open's open peer review process please email info.bmjopen@bmj.com

BMJ Open

Cognitive function and treatment response trajectories in first episode schizophrenia: evidence from a prospective cohort study.

Journal:	*BMJ Open*
Manuscript ID	bmjopen-2022-062570
Article Type:	Original research
Date Submitted by the Author:	04-Mar-2022
Complete List of Authors:	Millgate, Edward; King's College London Institute of Psychiatry Psychology and Neuroscience, Department of Psychosis Studies<br>Griffiths, Kira; King's College London Institute of Psychiatry Psychology and Neuroscience, Department of Psychosis Studies<br>Egerton, Alice; King's College London Institute of Psychiatry Psychology and Neuroscience, Department of Psychosis Studies; South London and Maudsley NHS Foundation Trust, NIHR Biomedical Research Centre<br>Kravariti, Eugenia; King's College London Institute of Psychiatry Psychology and Neuroscience, Department of Psychosis Studies; South London and Maudsley NHS Foundation Trust, NIHR Biomedical Research Centre<br>Casetta, Cecilia; King's College London Institute of Psychiatry Psychology and Neuroscience, Department of Psychosis Studies; South London and Maudsley Mental Health NHS Trust, National Psychosis Service<br>Deakin, JFW; University of Manchester, Faculty of Medicine, Biology and Health; Greater Manchester Mental Health NHS Foundation Trust<br>Drake, Richard; University of Manchester, Division of Psychology & Mental Health; Greater Manchester Mental Health NHS Foundation Trust<br>Howes, Oliver; King's College London Institute of Psychiatry Psychology and Neuroscience, Department of Psychosis Studies; South London and Maudsley NHS Foundation Trust, NIHR Biomedical Research Centre<br>Kassoumeri, Laura; King's College London Institute of Psychiatry Psychology and Neuroscience, Department of Psychosis Studies<br>Khan , Sobia; University of Manchester, Faculty of Medicine, Biology and Health<br>Lankshear, Steve; Greater Manchester Mental Health NHS Foundation Trust<br>Lees, Jane; Greater Manchester Mental Health NHS Foundation Trust; University of Manchester, Faculty of Medicine, Biology and Health<br>Lewis, Shon; University of Manchester; Greater Manchester Mental Health NHS Foundation Trust<br>Mikulskaya, Elena ; University of Manchester, Faculty of Medicine, Biology and Health<br>Oloyede, Ebenezer; King's College London Institute of Psychiatry Psychology and Neuroscience, Department of Psychosis Studies; South London and Maudsley NHS Foundation Trust, Pharmacy Department<br>Owens, Rebecca; University of Manchester, Faculty of Medicine, Biology and Health<br>Pollard, Rebecca; King's College London Institute of Psychiatry

Psychology and Neuroscience, Department of Psychosis Studies
Rich, Nathalie; University College London, Department of Epidemiology & Applied Clinical Research
Smart, Sophie; Cardiff University, Division of Psychological Medicine and Clinical Neurosciences
Segev , Aviv; King's College London Institute of Psychiatry Psychology and Neuroscience, Department of Psychosis Studies; Shalvata Mental Health Center, Sackler Faculty of Medicine
Verena Sendt , Kyra; King's College London Institute of Psychiatry Psychology and Neuroscience, Department of Psychosis Studies
MacCabe, James; Institute of Psychiatry Psychology and Neuroscience Division of Academic Psychiatry, Department of Psychosis Studies; South London and Maudsley NHS Foundation Trust, National Psychosis Service

Keywords: Schizophrenia & psychotic disorders < PSYCHIATRY, Adult psychiatry < PSYCHIATRY, MENTAL HEALTH

SCHOLARONE™
Manuscripts

I, the Submitting Author has the right to grant and does grant on behalf of all authors of the Work (as defined in the below author licence), an exclusive licence and/or a non-exclusive licence for contributions from authors who are: i) UK Crown employees; ii) where BMJ has agreed a CC-BY licence shall apply, and/or iii) in accordance with the terms applicable for US Federal Government officers or employees acting as part of their official duties; on a worldwide, perpetual, irrevocable, royalty-free basis to BMJ Publishing Group Ltd ("BMJ") its licensees and where the relevant Journal is co-owned by BMJ to the co-owners of the Journal, to publish the Work in this journal and any other BMJ products and to exploit all rights, as set out in our licence.

The Submitting Author accepts and understands that any supply made under these terms is made by BMJ to the Submitting Author unless you are acting as an employee on behalf of your employer or a postgraduate student of an affiliated institution which is paying any applicable article publishing charge ("APC") for Open Access articles. Where the Submitting Author wishes to make the Work available on an Open Access basis (and intends to pay the relevant APC), the terms of reuse of such Open Access shall be governed by a Creative Commons licence – details of these licences and which Creative Commons licence will apply to this Work are set out in our licence referred to above.

Other than as permitted in any relevant BMJ Author's Self Archiving Policies, I confirm this Work has not been accepted for publication elsewhere, is not being considered for publication elsewhere and does not duplicate material already published. I confirm all authors consent to publication of this Work and authorise the granting of this licence.

For peer review only

Journal: BMJ Open

Abstract word count: 295

Word count: 3582

Submission date: 4th March 2022

Cognitive function and treatment response trajectories in first episode schizophrenia: evidence from a prospective cohort study.

Edward Millgate[1], Kira Griffiths[1], Alice Egerton[1,2], Eugenia Kravariti[1,2], Cecilia Casetta[1,3], Bill Deakin[4,5], Richard J Drake[4,5,6], Oliver D Howes[1,2], Laura Kassoumeri[1], Sobia Khan[4], Steve Lankshear[5], Jane Lees[4,5,6], Shôn Lewis[4,5,6], Elena Mikulskaya[4], Ebenezer Oloyede[1,7], Rebecca Owens[4], Rebecca Pollard[1], Nathalie Rich[8], Sophie E Smart[9], Aviv Segev[1,10,11], Kyra-Verena Sendt[1], The STRATA Consortium, James H MacCabe[1,3]*

[1] Department of Psychosis Studies, Institute of Psychiatry, Psychology and Neuroscience, King's College London, UK

[2] NIHR Biomedical Research Centre at South London and Maudsley NHS Foundation Trust, London, UK

[3] National Psychosis Service, South London and Maudsley NHS Foundation Trust, London, UK

[4] Division of Psychology and Mental Health, School of Biological Sciences, Faculty of Biology, Medicine and Health, University of Manchester, Manchester, UK

[5] Greater Manchester Mental Health NHS Foundation Trust, Manchester, UK

[6] Manchester Academic Health Science Centre, Manchester, UK

[7] Pharmacy Department, South London and Maudsley NHS Foundation Trust, UK

[8] Department of Epidemiology & Applied Clinical Research, University College London, UK

[9] MRC Centre for Neuropsychiatric Genetics and Genomics, Division of Psychological Medicine and Clinical Neurosciences, Cardiff University, Cardiff, UK

[10] Shalvata Mental Health Centre, Israel

[11] Sackler Faculty of Medicine, Tel Aviv University, Israel

*Corresponding author: Edward.millgate@kcl.ac.uk

For peer review only

Department of Psychosis Studies,

Institute of Psychiatry, Psychology & Neuroscience,

16 de Crespigny Park,

Denmark Hill, London,

United Kingdom, SE5 8AF

For peer review only

Abstract

Objectives: This prospective cohort study tested for associations between baseline cognitive performance in individuals early within their first episode and antipsychotic treatment of psychosis. We hypothesised that poorer cognitive functioning at the initial assessment would be associated with poorer antipsychotic response following the subsequent six weeks.

Setting: NHS service users with a first episode schizophrenia diagnosis, recently starting antipsychotic medication, recruited from two UK sites (King's College London, UK & University of Manchester, UK). Participants attended three study visits following screening.

Participants: Eighty-nine participants were recruited, with 46 included in the main analysis. Participants required to be within the first two years of illness onset, had received minimal antipsychotic treatment, have the capacity to provide consent, and be able to read and write in English. Participants were excluded if they met remission criteria or showed mild to no symptoms.

Primary and secondary outcomes: Antipsychotic response was determined at 6-weeks using the Positive and Negative Syndrome Scale (PANSS), with cognitive performance assessed at each visit using the Brief Assessment of Cognition in Schizophrenia (BACS). The groups identified (responders and non-responders) from trajectory analyses, as well as from >20% PANSS criteria, were compared on baseline BACS performance.

Results: Trajectory analyses identified 84.78% of the sample as treatment responsive, and the remaining 15.22% as treatment non-responsive. Unadjusted and adjusted logistic regressions observed no significant relationship between baseline cognitive performance and antipsychotic response.

Conclusions: This investigation identified two clear trajectories of treatment response in the first six weeks of antipsychotic treatment. Responder and non-responder groups did not significantly differ on performance on the BACS, suggesting that larger samples may be required or that brief cognitive batteries for schizophrenia may not be a useful predictor of response in the first two-years of illness onset.

Trial registration number: REC: 17/NI/0209

Keywords: cognition, antipsychotic response, BACS, trajectories, first episode schizophrenia

For peer review only

Article summary

Strengths and limitations of this study

- The study examined baseline cognitive performance in a relatively large sample of first-episode schizophrenia, with patients recently starting antipsychotic treatment.
- Trajectory analyses used identified two clear patterns of antipsychotic response at 6-weeks after baseline assessment.
- The lack of significant group differences in baseline cognitive performance between antipsychotic responders and non-responders may be a result of under-sampled comparisons or that brief cognitive batteries for schizophrenia may not be a useful predictor of response in the first two-years of illness onset.

For peer review only

Introduction

Prompt intervention with pharmacological therapy for individuals with schizophrenia has been extensively recommended in the literature[1,2] and is reported to be associated with better functional outcomes[3,4,5]. As observed by Carbon & Correll[5], a lack of early response and improvement to antipsychotic medication is a strong predictor of later non-response. A recent diagnostic test review has even argued that non/minimal response to antipsychotic medication in the first 2 weeks of treatment may be a sufficient indication to switch antipsychotic[6]. Early and accurate detection of treatment non-responders at first episode is also more likely to result in timely treatment with clozapine, which may be associated with better outcomes[7]. Indeed, Yoshimura et al[8] found that response to clozapine was ~80% in treatment resistant patients who were commenced on clozapine early in their illness course, with this depreciating to ~30% when clozapine initiation was delayed by more than 2.8years[7,8].

Early cognitive deficits may be predictive of subsequent antipsychotic response. Cognitive dysfunction in schizophrenia is observable prior to illness onset[9,10] and is strongly associated with poorer functional outcomes[11,12,13]. A recent meta-analysis comparing cognitive performance in known cases of antipsychotic treatment resistance and response[14] observed worse performance in treatment resistant samples across cognitive domains, with the strongest effect in measures of verbal memory and learning and language functions. However, it is possible that illness chronicity and exposure to long-term antipsychotic treatment may have influenced these findings.

This prospective cohort study tested for associations between baseline cognitive performance, assessed at the initiation of antipsychotic treatment, in individuals early within their first episode of psychosis and their subsequent response to antipsychotic treatment. We hypothesised that poorer cognitive functioning at the initial assessment would be associated with poorer response over the subsequent six weeks of antipsychotic treatment.

Methods

For peer review only

Design

The study used a prospective cohort design with a sample of patients with first-episode schizophrenia. Participants were assessed over a period of 6-weeks, with two follow-up visits following baseline and screening assessments.

Setting

The study was part of the 'Schizophrenia: Treatment Resistance and Therapeutic Advances' (STRATA) consortium which included two UK sites in this study; King's College London (London, UK) and University of Manchester (Manchester, UK). The aim of the STRATA consortium is to identify neurobiological, cognitive, and genetic biomarkers of antipsychotic treatment resistance and non-response within schizophrenia and other related psychotic disorders.

Patient and Public Involvement

In the early development and design of the study consultations with the NIHR Maudsley Biomedical Research Centre (BRC) Service User Advisory Group (SUAG) took place to determine the feasibility of the study and its' assessments for service users. The NIHR Maudsley BRC Feasibility and Acceptability Support Team for Researchers (FAST-R) service was also used in order to receive feedback on consent forms, information sheets and protocols, as well as advice for recruitment strategies for service users.

Participants

89 participants aged between 18 – 65 years with a DSM-5 diagnosis of schizophrenia, schizoaffective, schizophreniform disorder, or psychosis (non-specified) (ICD-10: F20-F29) were recruited across two UK sites (King's College London and University of Manchester). Inclusion required that participants were within the first two years of illness onset, defined using the date of first initial contact with services and clinical records. Inclusion also required that participants had received minimal antipsychotic medication, which was defined as having received antipsychotic treatment for no longer than 4 weeks prior to the baseline visit, after a period of being either antipsychotic naïve or antipsychotic-free for at least 14 days. Participants also were required to have the capacity to provide consent and the ability to read and write in English. Participants were excluded if they met modified Andreasen remission criteria[15], having mild or less scores on all of the following Structured Clinical Interview-Positive and Negative Syndrome Scale (SCI-PANSS)[23] items: delusions (P1),

For peer review only

conceptual disorganisation (P2), hallucinatory behaviour (P3), blunted affect (N1), social withdrawal (N4), lack of spontaneity (N6), mannerisms/posturing (G5), unusual thought content (G9) on the day of assessment, as this would suggest that their symptoms were in remission.

Participants were assessed at baseline (± 7days), 2-weeks (±7days) and 6-weeks (±7 days), with the maximum cut-off for 6-week follow up being 78 days after baseline assessment. Participants were reimbursed for their time and expenses for participation in the study. Twenty-six participants were withdrawn after providing consent, an additional 11 were withdrawn following baseline, and another 6 participants withdrawn following 2-week assessment. Participants were withdrawn if they were unable to attend the study visit, their symptoms were in remission (as per Andreasen remission criteria[15]), or if they no longer wanted to take part in the study and requested to have their data removed. 46 participants were eligible for inclusion in the analysis. All participants gave informed consent prior to enrolment. This study was approved by the Health and Social Care Research Ethics Committee A; REC: 17/NI/0209. All participants provided informed consent prior to participation.

Definitions for treatment response status

Treatment response groups were modelled through trajectory analyses using the t*raj* command in STATA[16]. This tool estimates group-based trajectories over a specified time interval, clustering individuals who follow similar trajectories through a censored normal model. Akaike information criterion (AIC) and Bayesian information criterion (BIC) values were used to select the trajectory model with the lowest AIC and BIC values. Linear trajectories of up to five classes (1 to 5 trajectories) were assessed for eligibility. Rescaled PANSS scores[17] for percentage change at weeks 2 and 6 were used in the model. The results generated using this trajectory grouping were also compared to a more standard definition of treatment response which uses a >20% reduction in rescaled PANSS total scores from initial to final assessment[18,19]. Here patients not reaching a 20% reduction in rescaled PANSS total scores at the 6-week visits were categorised as non-responsive. These results are reported in the supplementary material.

Materials

Clinical and demographic measures

At baseline, participants completed the Kemp Clinician Rating Scale (CRS)[20,21], Mini-international neuropsychiatric interview (MINI)[22] (M-Psychotic Disorders; A-Major Depressive Episode; D-Manic/Hypomanic/Bipolar), Structured Clinical Interview- Positive and Negative Syndrome Scale (SCI-PANSS)[23], Clinician Rating Scale for Schizophrenia (CGI-SCH)[24] and provided demographic data. At each subsequent study visit the CRS, SCI-PANSS and CGI-SCH were repeated.

Neuropsychological assessment

Participants completed Version A of the Brief Assessment of Cognition in Schizophrenia (BACS)[25] at each study visit. The BACS was originally developed to assess cognitive functioning in schizophrenia, while also being an easily administrable and brief test battery[25]. The battery includes tasks pertaining to executive functions, working memory, motor/processing speed, verbal memory, verbal fluency, and attention cognitive domains. Version A includes the following tasks: i) list learning task (verbal memory); ii) digit sequencing task (working memory); iii) token motor task (motor speed); iv) category instances task (verbal fluency); v) symbol coding task (attention and speed of information processing) and vi) tower of London (executive function). All tasks on the BACS are scored such that higher scores represent better performance. Composite t and z scores for the BACS were generated using scores from published normative data[26].

Data analysis

All analyses were conducted in STATA 15/SE[27]. Independent t-tests were used to compare cognitive performance and symptom severity in the whole sample between visits (i.e. baseline assessment to 2-week, 2-week to 6-week, and baseline to 6-week). Baseline cognitive performance on the BACS was compared between trajectory groups using multivariable logistic regressions on the BACS composite and subscale scores. All models were adjusted for age, gender, and days from 1st psychotic symptom to baseline antipsychotic medication (i.e. duration of untreated psychosis; DUP). Results were then compared to

For peer review only

groupings based on >20% reduction in rescaled PANSS total scores[18,19] from baseline assessment to 6-week follow-up (supplementary material Table S.2).

Finally, growth curve models were executed using the *xtmixed* command[28] to compare cognitive performance over time between trajectory groups to estimate any changes in cognitive performance over the study period (supplementary material Table S.3). These results were again compared to >20% PANSS reduction criteria for treatment response (supplementary material Table S.4). .

Results

Table 1 reports the demographic descriptive of the whole sample included in analysis (N = 46) at baseline, with PANSS symptom severity scores and BACS performance for each study visit illustrated in Table 2. Data regarding antipsychotic medication was provided by all participants at baseline, all of which were treated with 2nd generation antipsychotics. At baseline 45 participants provided PANSS symptom severity ratings, with 41 providing at least one baseline measure of the BACS (Table 2).

Between study visits, a significant improvement in PANSS positive symptoms scores was observed in the whole sample between baseline and 2-week visits, as well as baseline and 6-week assessments (Table 2). A significant improvement in PANSS total scores was observed between baseline and 6-week visits.

Table 1

Descriptive statistics of the whole sample demographics at consent (age) and baseline assessments

Variable	N	M	SD	Min	Max
Age (at consent)	46	27.30	8.17	18	50
Gender (*male*)	33 (71.74%)	-	-	-	-
Gender (*female*)	13 (28.26%)	-	-	-	-

For peer review only

Age of illness onset (years)	46	26.53	8.45	18	49
Duration from 1st Psychotic symptom (days) to baseline antipsychotic (DUP)	46	248.30	245.06	0	726
Duration from 1st contact with mental health services (days) to baseline antipsychotic	46	346.57	600.37	6	2358
Chlorpromazine equivalents (mg/day)	46	176.89	121.29	10	800
Number of hospitalisations	46	0.89	0.64	0	3
Years of education	42	13.62	2.82	5	20
CGI-SCH baseline score	56	Minimally ill = 1<br>Mildly ill = 4<br>Moderately ill = 12<br>Markedly ill = 15<br>Severely ill = 12<br>Among the most severely ill = 1	-	-	-
Antipsychotic medication	51	Amisulpride = 1<br>Aripiprazole = 19<br>Olanzapine = 16<br>Paliperidone = 1<br>Quetiapine = 4<br>Risperidone = 5	-	-	-

Note. *DUP* = Duration of untreated psychosis; *CGI-SCH* = Clinical Rating Scale for Schizophrenia.

For peer review only

Table 2

Mean symptom severity as rated by the PANSS and BACS performance for the whole sample for each study visit

	Baseline			2-week follow-up			6-week follow-up			Baseline vs 2-week	2 -week vs 6-week	Baseline vs 6-week
Variable	N	M	SD	N	M	SD	N	M	SD	T-test	T-test	T-test
PANSS positive	45	11.93	4.77	36	9.17	5.36	38	7.26	5.13	t(79) = 2.45, p = .016, 95CIs = 0.52 ; 5.01	t(72) = 1.56, p = .123, 95CIs = -0.53 ; 4.34	t(81) = 4.29, p < .001, 95CIs = 2.50 ; 6.84
PANSS negative	45	10.36	6.87	36	10.31	7.15	38	9.58	7.78	t(79) = 0.03, p = .975, 95CIs = -3.06 ; 3.16	t(72) = 0.42, p = .677, 95CIs = -2.74 ; 4.19	t(81) = 0.48, p = .630, 95CIs = -2.42 ; 3.98
PANSS general	45	19.40	8.57	36	16.64	10.60	38	15.74	10.11	t(79) = 1.30, p = .198, 95CIs = -1.48 ; 7.00	t(72) = 0.38, p = .709, 95CIs = -3.90 ; 5.70	t(81) = 1.79, p = .078, 95CIs = -0.42 ; 7.74
PANSS total	45	41.69	16.11	36	36.11	20.03	38	32.58	20.04	t(79) = 1.39, p = .169, 95CIs = -2.41 ; 13.57	t(72) = 0.76, p = .451, 95CIs = -5.76 ; 12.82	t(81) = 2.30, p = .024, 95CIs = 1.22 ; 17.00
BACS Verbal memory	41	37.83	14.02	32	41.16	13.50	36	44.56	15.02	t(71) = -1.02, p = .310, 95CIs = -9.82 ; 3.16	t(66) = -0.98, p = .332, 95CIs = -10.35 ; 3.55	t(75) = -2.03, p = .046, 95CIs = -12.32 ; -0.13
BACS Digit sequencing	38	18.03	4.06	32	17.84	4.33	34	17.85	4.69	t(68) = 0.18, p = .856, 95CIs = -1.82 ; 2.19	t(64) = -0.01, p = .993, 95CIs = -2.23 ; 2.21	t(70) = 0.17, p = .867, 95CIs = -1.88 ; 2.23
BACS Verbal fluency	42	28.60	7.85	31	31.87	9.47	35	30.20	8.38	t(71) = -1.61, p = .111, 95CIs = -7.32 ; 0.77	t(64) = 0.76, p = .450, 95CIs = -2.72 ; 6.06	t(75) = -0.87, p = .389, 95CIs = -5.30 ; 2.09

For peer review only

BACS Token motor	39	65.36	10.83	32	65.56	17.81	34	61.38	20.94	t(69) = -0.06, p = .953, 95CIs = -7.05 ; 6.64	t(64) = 0.87, p = .387, 95CIs = -5.41 ; 13.77	t(71) = 1.04, p = .303, 95CIs = -3.66 ; 11.62
BACS Symbol coding	39	40.15	13.13	32	45.69	12.56	32	47.25	11.96	t(69) = -1.80, p = .076, 95CIs = -11.66 ; 0.59	t(62) = -0.51, p = .612, 95CIs = -7.69 ; 4.57	t(69) = -2.36, p = .011, 95CIs = -13.10 ; -1.09
BACS ToL	37	14.84	4.48	29	17.38	3.29	32	16.28	4.13	t(64) = -2.56, p = .013, 95CIs = -4.53 ; -0.56	t(59) = 1.14, p = .259, 95CIs = -0.83 ; 3.02	t(67) = -1.38, p = .171, 95CIs = -3.53 ; 0.64
BACS t-score	33	26.67	11.98	28	34.14	11.68	30	30.87	14.95	t(59) = -2.46, p = .017, 95CIs = -13.57 ; -1.39	t(56) = 0.93, p = .359, 95CIs = -3.81 ; 10.37	t(61) = -1.24, p = .221, 95CIs = -11.00 ; 2.60
BACS z-score	33	-2.34	1.19	28	-1.59	1.17	-1.91	1.50		t(59) = -2.50, p = .015, 95CIs = -1.26 ; -0.15	t(56) = 0.93, p = .359, 95CIs = -0.38 ; 1.04	t(61) = -1.27, p = .211, 95CIs = -1.11 ; 0.25

Note. *PANSS* = Positive and Negative Symptom Scale; *BACS* =Brief Assessment of Cognition in Schizophrenia; *ToL* = Tower of London.

For peer review only

Trajectories of symptom change

BIC and AIC values were generated for five classes of trajectory models (Table 3). Of these both indices indicate that the two-trajectory group is best fitted to the data. This model estimated 73.7% of the sampled population to be from one linear trajectory, with the remaining 26.3% in another.

Table 3
Selecting a trajectory model using BIC and AIC estimates

No. of classes	1	2	3	4	5
BIC	-522.21	-512.13	-517.87	-520.14	-525.88
AIC	-519.46	-506.64	-509.64	-509.17	-512.17
%. in each class	100%	73.7% ; 26.3%	73.7% ; 26.3%; 0%	60.7% ; 23.7%; 15.6%; 0%	60.7% ; 23.7%; 15.6%; 0%; 0%

Note. *BIC* = Bayesian Information Criteria; *AIC* = Akaike's Information Criteria.

The trajectories identified by the *traj* procedure are shown in Figure 1. The trajectories that emerged clearly represented responders versus non-responders. 39 participants (84.78%) of the sample were classified as antipsychotic treatment responsive and 7 as treatment non-responsive (15.22%). For responders, PANSS total score percentage change at 6 weeks was on average -32.89% symptom improvement. For non-responders this was 21.03% indicating a minimal and, in some cases, worsening in symptom severity. Shape estimates and standard errors of antipsychotic response are shown in Table 4. Treatment responders significantly improved over the 6-week period. Descriptive statistics of clinical and demographic variables between both trajectory groups (non-responder; responder) are presented in the supplementary material (Table S.1.) In comparison to those responsive to antipsychotic medication, non-responders were on average older, had a longer duration of time from first contact with mental health services to baseline antipsychotic medication, had marginally more hospitalisations, attained more years of education, and were treated at higher chlorpromazine equivalents (supplementary material Table S.1).

For peer review only

[Figure 1]

Table 4

Parameter estimates and standard errors for both trajectories of antipsychotic response

Parameters	Trajectories	
	Non-responder (N = 7)	Responder (N = 39)
Intercept	2.54	-3.71
Linear change	0.10	-0.54
Standard error	0.06	0.09
T statistic	1.61, p = .111	-6.06, p < .001

Cognitive performance

There was a significant improvement in BACS verbal memory and symbol coding performance between baseline and 6-week assessments across the whole sample, with a significant improvement in Tower of London and BACS z and t composite scores between baseline and 2-week visits (Table 2). At baseline assessment, there was no difference in the BACS subscale or composite scores between antipsychotic responders and non-responders identified in the trajectory analysis. (Table 5; Table 6). Growth curve models observed no significant change in cognitive performance over follow-up visits between trajectory groups (supplementary material Figure S.1, Table S.3). A similar pattern in results was observed when >20% PANSS reduction criteria was applied (supplementary material Figure S.2, Table S.4, Table S.5).

For peer review only

Table 5

Baseline cognitive performance for both trajectory groups

	Non-responder			Responder		
BACS measure	N	Mean	SD	N	Mean	SD
Verbal Memory	7	37.29	9.48	34	37.94	14.89
Digit Sequencing	6	20.17	5.38	32	17.63	3.74
Verbal Fluency	7	30.29	7.30	35	28.26	8.01
Token Motor	7	66.86	8.93	32	65.03	11.30
Symbol Coding	6	47.50	6.35	33	38.82	13.66
Tower of London	7	14.71	3.77	30	14.87	4.69
tscore composite	6	28.83	14.36	27	26.19	11.65
zscore composite	6	-2.12	1.41	27	-2.39	1.16

Note. *BACS* =Brief Assessment of Cognition in Schizophrenia.

Multivariable linear regression

Univariable and multivariable logistic regression models adjusting for age and gender and duration of untreated psychosis (DUP) found no significant associations between BACS performance at baseline and response trajectory over 6 weeks (Table 6), with no association of any demographic or clinical variables in multivariable models. This was also observed when utilising the >20% reduction in PANSS total criteria (supplementary material Table S.2).

For peer review only

Table 6

Results from univariable and multivariable logistic regression models for response status and baseline BACS performance

	Unadjusted					Adjusted for age, gender, and DUP				
BACS task	β	SE	95%CI	OR	P-value	β	SE	95%CI	OR	P-value
Verbal Memory	<0.01	0.03	-0.06 ; 0.06	1.00	.909	<-0.01	0.03	-0.07 ; 0.06	1.00	.918
Digit Sequencing	-0.17	0.12	-0.41 ; 0.07	0.84	.168	-0.18	0.13	-0.44 ; 0.07	0.83	.151
Verbal Fluency	-0.03	0.05	-0.14 ; 0.07	0.97	.530	-0.05	0.06	-0.17 ; 0.07	0.95	.417
Token Motor	-0.02	0.04	-0.09 ; 0.06	0.98	.683	-.0.02	0.05	-0.11 ; 0.08	0.99	.737
Symbol Coding	-0.06	0.04	-0.14 ; 0.02	0.94	.145	-0.07	0.05	-0.16 ; 0.02	0.93	.114
Tower of London	0.08	0.09	-0.18 ; 0.19	1.01	.935	-0.01	0.10	-0.20 ; 0.19	0.99	.947
t score composite	-0.02	0.04	-0.10 ; 0.06	0.98	.620	-0.02	0.04	-0.10 ; 0.06	0.98	.594
z score composite	-0.21	0.40	-0.99 ;0.58	0.81	.603	-0.23	0.41	-1.02 ;0.57	0.80	.573

Note. BACS = Brief Assessment of Cognition in Schizophrenia; *CIs* = confidence intervals; *DUP* = duration of untreated psychosis.

For peer review only

For peer review only

Discussion

This prospective study investigated the relationship between baseline cognitive performance and subsequent antipsychotic response over a 6-week treatment period. Across the whole sample, participants showed an overall reduction in symptom severity as well as an improvement in cognitive performance on the majority of BACS tasks. Trajectory analyses estimated two trajectory groups (73.7%, 26.3%) based on PANSS total % change from baseline; this was reflected as 84.78% of the sample being grouped as treatment responsive, and the remaining 15.22% as treatment non-responsive. Contrary to our hypothesis, baseline cognitive performance did not significantly differ between those identified as treatment responders or non-responders following 6 weeks of antipsychotic treatment. This finding remained the same when treatment response was defined as at least a 20% reduction in PANSS total scores.

Across the two- and six-week follow-up visits, an improvement in cognitive performance was observed for the whole sample verbal memory, symbol coding and Tower of London tasks, as well as the BACS composite scores. Most of these changes occurred between baseline and 2-week assessment (Table 2), with small decreases in performance on measures of verbal fluency, token motor and Tower of London tasks between 2-week and 6-week assessments, as well as for composite z and t scores. In contrast, there was a decline in performance in the token motor task across the follow-up period, and minimal changes in performance in the digit sequencing task.

The observed improvement in cognitive performance may reflect a beneficial outcome of antipsychotic treatment. 1st generation antipsychotics, introduced in the 1950s, target the positive symptoms observed in schizophrenia by acting as an antagonist at dopamine D2 receptors. Treatment with this group of antipsychotic drugs has been associated with motor and cognitive deficits in patients[29,30]. In contrast, 2nd generation antipsychotics are reported to have fewer extrapyramidal side effects[31], with these drugs also acting as an antagonist at the serotonin 5HT2A receptor, in addition to D2 dopamine receptors. Research suggests that in comparison to 1st generation, 2nd generation antipsychotics can provide some improvement in cognitive performance (e.g. clozapine[32]). Guilera et al (2009)[33] found in their meta-analysis of 18 randomised controlled trials that 2nd generation drugs provided a slight improvement in performance for global cognition, as well as slight but significant

For peer review only - http://bmjopen.bmj.com/site/about/guidelines.xhtml

improvements in measures of procedural learning, language and verbal comprehension, verbal learning and memory and visual learning and memory.

As the whole study sample was treated with 2nd generation antipsychotic drugs at baseline assessment (Amisulpride = 1, Aripiprazole = 19, Olanzapine = 16, Paliperidone = 1, Quetiapine = 4 and Risperidone = 5), it is possible that the improvement in cognitive performance observed in our sample may be a result of 2nd antipsychotic treatment effects, although 1st generation antipsychotic use could not be compared. However it has also been argued that improvements in cognitive performance over longitudinal designs may instead reflect practice effects (e.g. familiarity and procedural learning[34]), meaning that improvement in cognitive performance in our sample could also be attributable to practice effects between study visits. Lees et al[35] estimated the magnitude of these effects using both the MATRICS Consensus Cognitive Battery (MCCB)[36] and the Cog State Schizophrenia Battery[37], finding strong test-retest correlations between repeated baseline visits across cognitive batteries, with potential learning effects in social-emotional cognition. However, the authors also observed that participants may have failed to complete the initial baseline assessment due to difficulty in understanding the task, with the suggestion that future investigations using either battery would benefit from adopting initial practice sessions to reduce practice effects. Therefore an initial practice session with the BACS may have reduced the size of improvement observed in cognitive performance from baseline performance.

Despite all of the sample being treated with 2nd generation antipsychotics, it is also possible that some anticholinergic effects, which differ between 2nd generation antipsychotic drugs[38], may have affected cognitive performance. Long term exposure to antipsychotic medications of high anticholinergic activity have been previously reported to impact cognitive performance in patient samples[39,40,41]. Using low and high anticholinergic activity criteria from a recent review comparing medication effects (from Stroup & Gray[42]; refer to Table 1, pg. 342), our sample had 44% (N = 20) treated with a high anticholinergic antipsychotic, meaning that the absence of significant differences between groups may have been a result of heterogeneity in medication effects. Therefore, future investigations should consider the role of antipsychotic treatment effects on cognitive outcomes within schizophrenia.

For peer review only - http://bmjopen.bmj.com/site/about/guidelines.xhtml

Trajectory analyses identified two clearly defined trajectories of treatment response, both of which are consistent across both timepoints: one trajectory showing good response, and one of little to no response (Figure 1). Confidence intervals (Figure 1) show some overlap between trajectories in the first ~20 days since baseline assessment, with these becoming independent following this period, meaning that separation between trajectory groups was apparent at around 3 weeks. This supports the findings from Samara et al[6] who found poor/minimal response to antipsychotic treatment at 2-weeks to be predictive of future treatment non-response. In previous investigations using first episode samples, 4 or more trajectories have been identified[43,44]. However, both these investigations used longer periods of follow-up as well as raw un-adjusted PANSS scores in their analyses: as the minimum raw score of the PANSS is 30, it is recommended to rescale the scores by subtracting 30 from total scores prior to producing percentages and ratios[45]. Therefore building trajectory models using raw scores may not be appropriate to use as ratio operations (e.g. calculating proportions) require a natural zero point[45].

Growth curve models which were used to quantify change in cognitive performance between trajectory groups observed no significant changes in performance between visits. It is possible that this may be due to under sampled groups, as significant improvements for verbal memory, symbol coding, Tower of London and composite scores were observed in the whole sample. When comparing our findings to a >20% reduction in rescaled PANSS total scores criteria[18,19] there were no changes in the pattern of results to growth curve models or logistic regression outcomes. Using this criterion for treatment response resulted in a more even distribution of the total sample to groups (responder = 17 ; non-responder = 21), providing more power to comparative analyses. However despite this there was no change in the pattern of results, meaning that this criterion provided no added benefit to this analysis over trajectory-based groupings. The lack of significant difference in baseline cognition between those classified as treatment responders or non-responders after 6 weeks of treatment in our study contrasts previous research conducted which observed impaired cognitive performance in the poor response trajectory at week 4, with good performance at baseline being predictive of a good response trajectory at week 4[44]. Likewise, longitudinal research using the MCCB[36] with first-episode schizophrenia patients assessed at baseline and at a 12-week follow up identified tasks of executive function and planning and reasoning ability as potential indices of antipsychotic response[46], with similar findings observed when cognitive performance is correlated with symptom severity measures[47].

For peer review only

Limitations

Previous investigations included sample sizes several magnitudes higher than in our study (Levine & Rabinowitz[44]; N = 491; Trampush et al[26]; N = 175) and it is likely that our sample size limited our ability to observe a significant relationship between cognitive performance and antipsychotic response. Using our sample's mean values for the BACS t composite score, a power calculation found that a total sample size of 31,304 samples would be required to detect a significant difference between trajectory groups at 90% power. When using the >20% PANSS reduction criteria this was N = 6,118, suggesting that both analyses were underpowered due to under sampling.

It is also possible that premorbid histories of the sample may have resulted in a less consistent picture of cognitive performance between groups. For example, prior cannabis use, particularly during adolescence, has been found to improve cognitive performance on the BACS in comparison to those who haven't ever used cannabis[48]. In this investigation comparing performance on the BACS between patients with a schizophrenia diagnosis with and without adolescent cannabis use (ACU), those with ACU reported significantly higher composite scores, as well significant improvement in working memory and verbal memory tasks (Hanna et al., 2016). In our sample, 68% (N = 30) had previous experience of using cannabis, with the majority of this use occurring between ages 12-19 (N = 23). Therefore, it is possible that premorbid histories may have also blurred the cognitive differences between groups.

Conclusions

In this prospective cohort study, patients with a first episode diagnosis were assessed three times over a period of 6 weeks. Trajectory analyses using percentage change in PANSS total symptom scores identified two groups reflecting a good and poor response to antipsychotic medication. Baseline cognitive performance of these two groups did not predict response status at 6-weeks. This lack of discrimination between groups is likely due to underpowered analyses as a result of small sample sizes. Overall this suggests that brief cognitive batteries for schizophrenia may not be a useful predictor of response in the first two-years of illness onset.

For peer review only

Funding

STRATA is funded by a grant from the Medical Research Council (MRC) to JHM, grant reference MR/L011794. EM's PhD is funded by the MRC-doctoral training partnership studentship in Biomedical Sciences at King's College London. J.H.M., E.K., A.E., O.D.H. are part funded by the National Institute for Health Research (NIHR) Biomedical Research Centre at South London and Maudsley NHS Foundation Trust and King's College London. The views expressed are those of the authors and not necessarily those of the NHS, the MRC, the NIHR or the Department of Health.

Competing interest

The remaining authors report no conflicts of interest.

Acknowledgments

For more information on STRATA please visit; https://gtr.ukri.org/project/7F7F0378-8FD7-4A33-91AD-277A49EF4908). STRATA was funded by a grant from the Medical Research Council (MRC) to Prof James MacCabe. Data were collected between June 2018 and December 2019. The remaining authors report no conflicts of interest.

Data availability

At the time of submission, the data governance frameworks are being put in place to make a fully anonymized version of the data available to the wider research community via the TranSMART data sharing platform: https://transmartfoundation.org/. To apply for access to the data, please contact J.H.M. at james.maccabe@kcl.ac.uk.

Author contributions

J.H.M., A.E., B.D., R.J.D., O.D.H., L.K., C.C., A.S., R.O., S.Le., J.L., S.La., S.K., and E.Mik contributed to the design and implementation of the study. E.O., E.M., R.P., N.R., K.G., C.C., S.E.S., and K.V.S. aided in data collection. E.M. completed analyses and wrote the manuscript with the assistance of J.H.M. K.G., N.R., J.H.M., A.E., E.K., R.J.D., A.S., C.C., B.D., provided comments on the manuscript.

For peer review only

References

1 McGlashan TH, Johannessen, JO. Early detection and intervention with schizophrenia: rationale. *Schizophrenia bulletin* 1996;22(2),201-222.

2 Birchwood M, McGorry P, Jackson H. Early intervention in schizophrenia. *The British Journal of Psychiatry* 1997;170(1),2-5.

3 Bond GR, Drake RE, Luciano A. Employment and educational outcomes in early intervention programmes for early psychosis: a systematic review. *Epidemiology and psychiatric sciences* 2015; 24(5),446-457.

4 Bozzatello P, Bellino S, Rocca P. Predictive factors of treatment resistance in first episode of psychosis: A systematic review. *Frontiers in psychiatry* 2019;10, 67.

5 Carbon, M, Correll CU. Clinical predictors of therapeutic response to antipsychotics in schizophrenia. *Dialogues in clinical neuroscience* 2014;16(4), 505.

6 Samara, MT, Leucht C, Leeflang M, et al. Early improvement as a predictor of later response to antipsychotics in schizophrenia: a diagnostic test review. *American journal of psychiatry* 2015;172(7),617-629.

7 Griffiths K, Millgate E, Egerton A, et al. Demographic and clinical variables associated with response to clozapine in schizophrenia: a systematic review and meta-analysis. *Psychological Medicine* 2021;1-11.

8 Yoshimura B, Yada Y, So R, et al. The critical treatment window of clozapine in treatment-resistant schizophrenia: secondary analysis of an observational study. *Psychiatry research* 2017;250,65-70.

9 Reichenberg A, Caspi A, Harrington H, et al. Static and dynamic cognitive deficits in childhood preceding adult schizophrenia: a 30-year study. *American Journal of Psychiatry* 2010;167(2),160-169.

10 MacCabe JH, Wicks S, Löfving S, et al. Decline in cognitive performance between ages 13 and 18 years and the risk for psychosis in adulthood: a Swedish longitudinal cohort study in males. *Jama Psychiatry* 2013;70(3),261-270.

11 Green MF, Kern RS, Heaton RK. Longitudinal studies of cognition and functional outcome in schizophrenia: implications for MATRICS. *Schizophrenia research* 2004;72(1),41-51.

12 Carrión RE, Goldberg TE, McLaughlin D, et al. Impact of neurocognition on social and role functioning in individuals at clinical high risk for psychosis. *American Journal of Psychiatry* 2011;168(8),806-813.

For peer review only - http://bmjopen.bmj.com/site/about/guidelines.xhtml

13 Horan WP, Green MF, DeGroot M, et al. Social cognition in schizophrenia, part 2: 12-month stability and prediction of functional outcome in first-episode patients. *Schizophrenia bulletin* 2012;38(4),865-872.

14 Millgate E, Hide O, Lawrie SM, et al. Neuropsychological differences between treatment-resistant and treatment-responsive schizophrenia: a meta-analysis. *Psychological medicine* 2022;52(1),1-13.

15 Andreasen NC, Carpenter JrWT, Kane JM, et al. Remission in schizophrenia: proposed criteria and rationale for consensus. *American Journal of Psychiatry* 2005;162(3),441-449.

16 Jones BL, Nagin DS. A note on a Stata plugin for estimating group-based trajectory models. *Sociological Methods & Research* 2013;42(4),608-613.

17 Leucht S, Kissling W, Davis JM. The PANSS should be rescaled. *Schizophrenia bulletin* 2010;36(3),461-462.

18 Leucht S, Kane JM, Kissling W, et al. What does the PANSS mean?. *Schizophrenia research* 2005;79(2-3),231-238.

19 Leucht S, Davis JM, Engel RR, et al. Definitions of response and remission in schizophrenia: recommendations for their use and their presentation. *Acta Psychiatrica Scandinavica* 2009;119,7-14.

20 Kemp R, Hayward P, Applewhaite G, et al. Compliance therapy in psychotic patients: randomised controlled trial. *Bmj* 1996;312(7027),345-349.

21 Kemp R, Kirov G, Everitt B, et al. Randomised controlled trial of compliance therapy: 18-month follow-up. *The British Journal of Psychiatry* 1998;172(5),413-419.

22 Sheehan DV, Lecrubier Y, Sheehan KH, et al. The Mini-International Neuropsychiatric Interview (MINI): the development and validation of a structured diagnostic psychiatric interview for DSM-IV and ICD-10. *The Journal of clinical psychiatry* 1998.

23 Kay SR, Fiszbein A, Opler LA. The positive and negative syndrome scale (PANSS) for schizophrenia. *Schizophrenia bulletin* 1987;13(2),261-276.

24 Guy W. ECDEU assessment manual for psychopharmacology. *US Department of Health, Education, and Welfare, Public Health Service, Alcohol, Drug Abuse, and Mental Health Administration, National Institute of Mental Health, Psychopharmacology Research Branch, Division of Extramural Research Programs* 1976.

25 Keefe RS, Goldberg TE, Harvey PD, et al. The Brief Assessment of Cognition in Schizophrenia: reliability, sensitivity, and comparison with a standard neurocognitive battery. *Schizophrenia research* 2004;68(2-3),283-297.

For peer review only

26 Keefe RS, Harvey PD, Goldberg TE, et al. Norms and standardization of the Brief Assessment of Cognition in Schizophrenia (BACS). *Schizophrenia research* 2008;102(1-3),108-115.

27 StataCorp. Stata Statistical Software: Release 15. 2017. College Station, TX: StataCorp LLC.

28 StataCorp, LP. Stata multilevel mixed-effects reference manual. 2013. College Station, TX: StataCorp LP, 9(10).

29 Kapur S, Zipursky R, Jones C, et al. Relationship between dopamine D2 occupancy, clinical response, and side effects: a double-blind PET study of first-episode schizophrenia. *American Journal of Psychiatry* 2000;157(4),514-520.

30 Reilly JL, Harris MS, Keshavan MS, et al. Adverse effects of risperidone on spatial working memory in first-episode schizophrenia. *Archives of general psychiatry* 2006;63(11),1189-1197.

31 Hill SK, Bishop JR, Palumbo D, et al. Effect of second-generation antipsychotics on cognition: current issues and future challenges. *Expert review of neurotherapeutics* 2010;10(1),43-57.

32 Kane J, Honigfeld G, Singer J, et al. Clozapine for the treatment-resistant schizophrenic: a double-blind comparison with chlorpromazine. *Archives of general psychiatry* 1988;45(9),789-796.

33 Guilera G, Pino O, Gómez-Benito J, et al. Antipsychotic effects on cognition in schizophrenia: a meta-analysis of randomised controlled trials. *The European Journal of Psychiatry* 2009;23(2),77-89.

34 Goldberg TE, Goldman RS, Burdick KE, et al. Cognitive improvement after treatment with second-generation antipsychotic medications in first-episode schizophrenia: is it a practice effect?. *Archives of general psychiatry* 2007;64(10),1115-1122.

35 Lees J, Applegate E, Emsley R, et al. Calibration and cross-validation of MCCB and CogState in schizophrenia. *Psychopharmacology* 2015;232(21),3873-3882.

36 Nuechterlein KH, Green MF, Kern RS, et al. The MATRICS Consensus Cognitive Battery, part 1: test selection, reliability, and validity. *American Journal of Psychiatry* 2008;165(2),203-213.

37 Maruff P, Thomas E, Cysique L, et al. Validity of the CogState brief battery: relationship to standardized tests and sensitivity to cognitive impairment in mild traumatic brain injury, schizophrenia, and AIDS dementia complex. *Archives of Clinical Neuropsychology* 2009;24(2),165-178.

38 Lieberman JA. Managing anticholinergic side effects. *Primary care companion to the Journal of clinical psychiatry* 2004;6(suppl2),20.

39 Strauss ME, Reynolds KS, Jayaram G, et al. Effects of anticholinergic medication on memory in schizophrenia. *Schizophrenia Research* 1990;3(2),127-129.

40 Campbell N, Boustani M, Limbil T, et al. The cognitive impact of anticholinergics: a clinical review. *Clinical interventions in aging* 2009;4,225.

41 Joshi YB, Thomas ML, Braff DL, et al. Anticholinergic Medication Burden–Associated Cognitive Impairment in Schizophrenia. *American Journal of Psychiatry* 2021;appi-ajp.

42 Stroup TS, Gray N. Management of common adverse effects of antipsychotic medications. *World Psychiatry* 2018;17(3),341-356.

43 Abdin E, Chong SA, Vaingankar JA, et al. Trajectories of positive, negative and general psychopathology symptoms in first episode psychosis and their relationship with functioning over a 2-year follow-up period. *PloS one* 2017;12(11),e0187141.

44 Levine SZ, Rabinowitz J. Trajectories and antecedents of treatment response over time in early-episode psychosis. *Schizophrenia bulletin* 2010;36(3),624-632.

45 Obermeier M, Mayr A, Schennach-Wolff R, et al. Should the PANSS be rescaled?. *Schizophrenia bulletin* 2010;36(3),455-460.

46 Trampush JW, Lencz T, DeRosse P, et al. Relationship of cognition to clinical response in first-episode schizophrenia spectrum disorders. *Schizophrenia bulletin* 2015;41(6),1237-1247.

47 Bilder RM, Goldman RS, Robinson D, et al. Neuropsychology of first-episode schizophrenia: initial characterization and clinical correlates. *American Journal of Psychiatry* 2000;157(4),549-559.

48 Hanna RC, Shalvoy A, Cullum CM, et al. Cognitive function in individuals with psychosis: moderation by adolescent cannabis use. Schizophrenia bulletin 2016;42(6),1496-1503.

For peer review only

Figure legend

Figure 1. Trajectory model of total PANSS score percentage change from baseline modelled over days since baseline assessment. The dotted linear trajectory reflects treatment non-responders and the complete line treatment responders. The grey dotted lines around each trajectory reflect the confidence intervals for trajectory each group. Percentages reflect the estimated amount of the sampled population included in each trajectory.

For peer review only

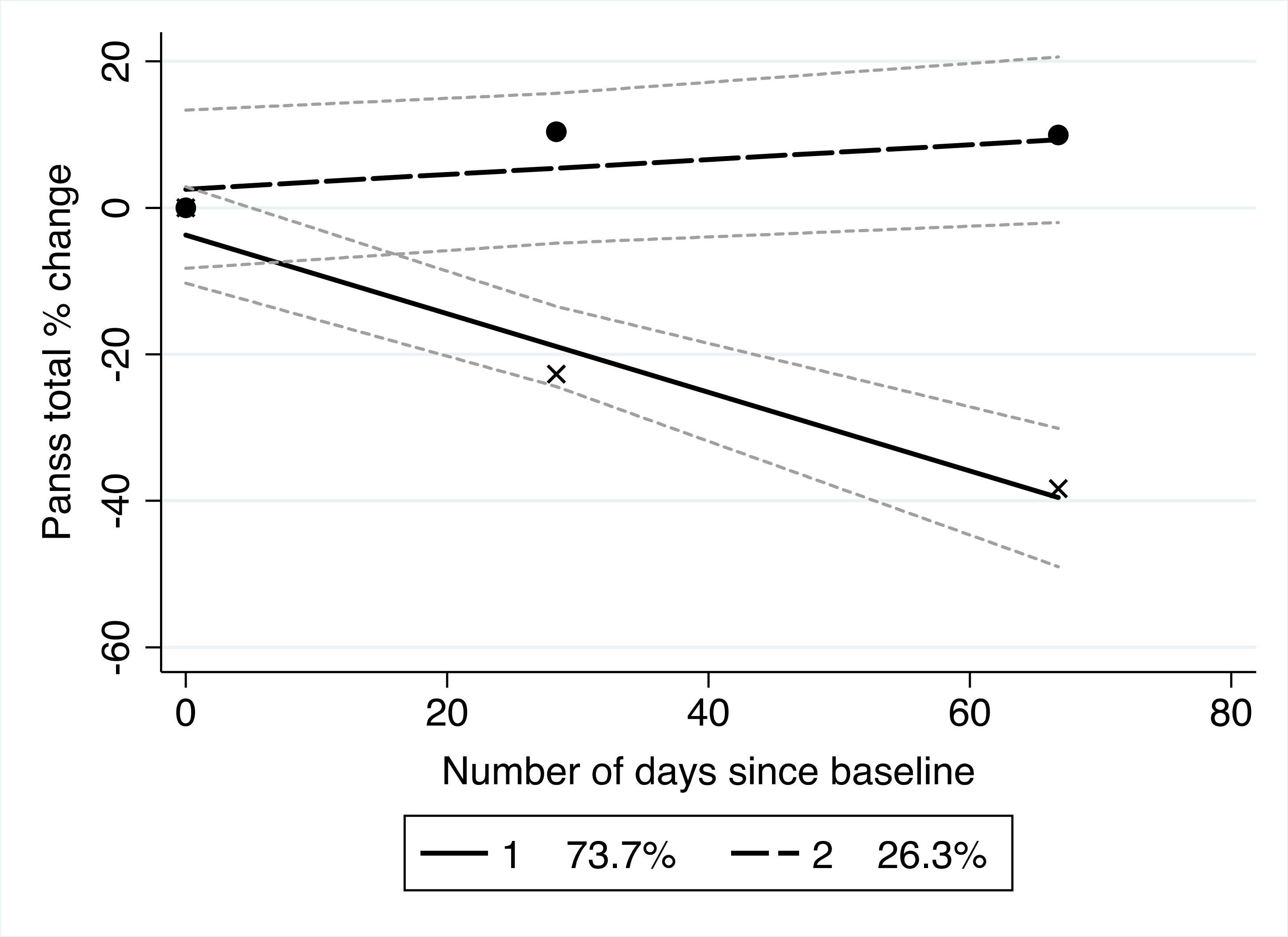

For peer review only

Supplementary material

Table S.1

Descriptive statistics of clinical and demographic variables for each trajectory group at baseline assessments

	Non-responder			Responder		
Variable	N	M	SD	N	M	SD
Age (at consent)	6	29.57	6.70	39	26.90	8.41
Gender (*male*)	6	-	-	27	-	-
Gender (*female*)	1	-	-	12	-	-
Age of illness onset (years)	7	27.54	8.09	39	26.34	8.60
Duration from 1st Psychotic symptom (days) to baseline antipsychotic (DUP)	7	177.09	207.93	39	261.08	251.38
Duration from 1st contact with mental health services (days) to baseline antipsychotic	7	461.89	801.88	39	325.88	567.82
Chlorpromazine equivalents (mg/day)	7	271.43	249.76	39	159.92	75.02
Number of hospitalisations	7	100	1.00	39	0.87	0.57
Years of education	6	17.00	2.53	36	13.06	2.47
PANSS positive	7	11.14	6.67	38	12.08	4.44
PANSS negative	7	12.57	5.77	38	9.95	7.04
PANSS general	7	19.57	8.70	38	19.37	8.66
PANSS total	7	43.29	13.52	38	41.39	16.68

Note. PANSS = Positive and Negative Symptom Scale.

For peer review only

Table S.2

Results from univariable and multivariable logistic regression models for response status using PANSS total >20% reduction criteria and baseline BACS performance

	Unadjusted					Adjusted for age, gender and DUP				
BACS task	β	SE	95%CI	OR	P-value	β	SE	95%CI	OR	P-value
Verbal Memory	-0.01	0.03	-0.07 ; 0.06	0.99	.819	<-0.01	0.04	-0.08 ; 0.07	1.00	.920
Digit Sequencing	-0.10	0.10	-0.29 ; 0.09	0.90	.297	-0.06	0.11	-0.27 ; 0.16	0.95	.604
Verbal Fluency	0.04	0.05	-0.06 ; 0.14	1.04	.403	0.07	0.06	-0.05 ; 0.18	1.07	.259
Token Motor	0.03	0.04	-0.04 ; 0.10	1.03	.432	0.06	0.05	-0.03 ; 0.15	1.06	.218
Symbol Coding	-0.04	0.03	-0.11 ; 0.02	0.96	.206	-0.05	0.04	-0.12 ; 0.03	0.96	.237
Tower of London	0.07	0.09	-0.11 ; 0.24	1.07	.445	0.07	0.09	-0.12 ; 0.25	1.07	.465
t score composite	<0.01	0.03	-0.06 ; 0.07	1.00	.924	0.01	0.03	-0.06 ; 0.07	1.00	.892
z score composite	0.02	0.33	-0.63 ; 0.66	1.02	.960	0.03	0.34	-0.63 ; 0.69	1.03	.931

Note. *BACS* = Brief Assessment of Cognition in Schizophrenia; *DUP* = duration of untreated psychosis; *CIs* = confidence intervals

For peer review only

Table S.3

Results from unadjusted and adjusted growth curve models comparing trajectory groups on BACS performance across all visits

	Unadjusted				Adjusted for age, gender and DUP			
BACS task	β	SE	95%CI	P-value	β	SE	95%CI	P-value
Verbal Memory	-2.15	45.79	-13.<br>49 ; 9.19	.710	-2.67	5.88	-14.20 ;8.86	.650
Digit Sequencing	-2.25	1.63	-5.45 ; 1<br>0.95	.167	-2.03	1.62	-5.19 ; 1.14	.210
Verbal Fluency	-0.90	3.29	-7.36 ; 5.55	.784	-1.31	2.99	-7.17 ; 4.54	.660
Token Motor	-2.00	6.39	-14.52 ;<br>10.53	.755	-0.45	5.89	11.99 ; 11.10	.939
Symbol Coding	-3.81	5.56	-14.71 ; 7.08	.493	-4.25	5.42	-14.87 ; 6.37	.433
Tower of London	0.18	1.56	-2.87 ; 3.24	.906	0.17	1.57	-2.90 ; 3.25	.912
t score composite	-2.49	5.70	-13.66 ; 8.68	.662	-2.43	5.78	-13.76 ; 8.89	.674
z score composite	-0.25	0.57	-1.37 ; 0.87	.658	-0.25	0.58	-1.38 ; 0.88	.665

Note. BACS = Brief Assessment of Cognition in Schizophrenia; *DUP* = duration of untreated psychosis; *CIs* = confidence intervals

For peer review only

Table S.4

Results from unadjusted and adjusted growth curve models comparing PANSS >20% reduction groups on BACS performance across all visits

	Unadjusted				Adjusted for age, gender and DUP			
BACS task	β	SE	95%CI	P-value	β	SE	95%CI	P-value
Verbal Memory	-4.18	4.21	-12.44 ; 4.08	.321	3.27	4.34	-11.78 ; 5.23	.451
Digit Sequencing	-2.36	1.35	-5.01 ; 0.29	.081	-1.78	1.34	-4.41 ; 0.86	.186
Verbal Fluency	-0.97	2.55	-5.97 ; 4.02	.703	-0.54	2.49	-5.42 ; 4.35	.830
Token Motor	1.81	5.52	-9.01 ; 12.62	.744	5.60	5.07	-4.33 ; 15.53	.269
Symbol Coding	-4.23	3.92	-11.92 ; 3.46	.281	-4.13	4.03	-12.03 ; 3.78	.306
Tower of London	-0.77	1.25	-3.22 ; 1.68	.538	-0.56	1.30	-3.10 ; 1.98	.667
t score composite	-2.39	4.75	-11.71 ; 6.93	.615	-1.56	4.84	-11.03 ; 7.92	.747
z score composite	-0.25	0.48	-1.18 ; 0.68	.600	-0.17	0.48	-1.12 ; 0.78	.728

Note. BACS = Brief Assessment of Cognition in Schizophrenia; *DUP* = duration of untreated psychosis; *CIs* = confidence intervals

For peer review only

Table S.5

Baseline cognitive performance for both groups using >20% PANSS reduction criteria

	Non-responder			Responder		
BACS measure	N	Mean	SD	N	Mean	SD
Verbal Memory	13	37.54	8.27	20	36.65	12.73
Digit Sequencing	12	19.25	4.20	19	17.68	3.97
Verbal Fluency	14	27.36	6.44	20	29.45	7.75
Token Motor	13	63.23	10.94	18	66.39	11.24
Symbol Coding	12	44.83	9.47	20	39.35	12.75
Tower of London	12	14.17	5.11	17	15.41	3.79
tscore composite	10	26.30	13.35	17	26.77	12.28
zscore composite	10	-2.36	1.31	17	-2.34	1.22

Note. *BACS* = Brief Assessment of Cognition in Schizophrenia; *PANSS* = Positive and Negative Symptom Scale.

For peer review only

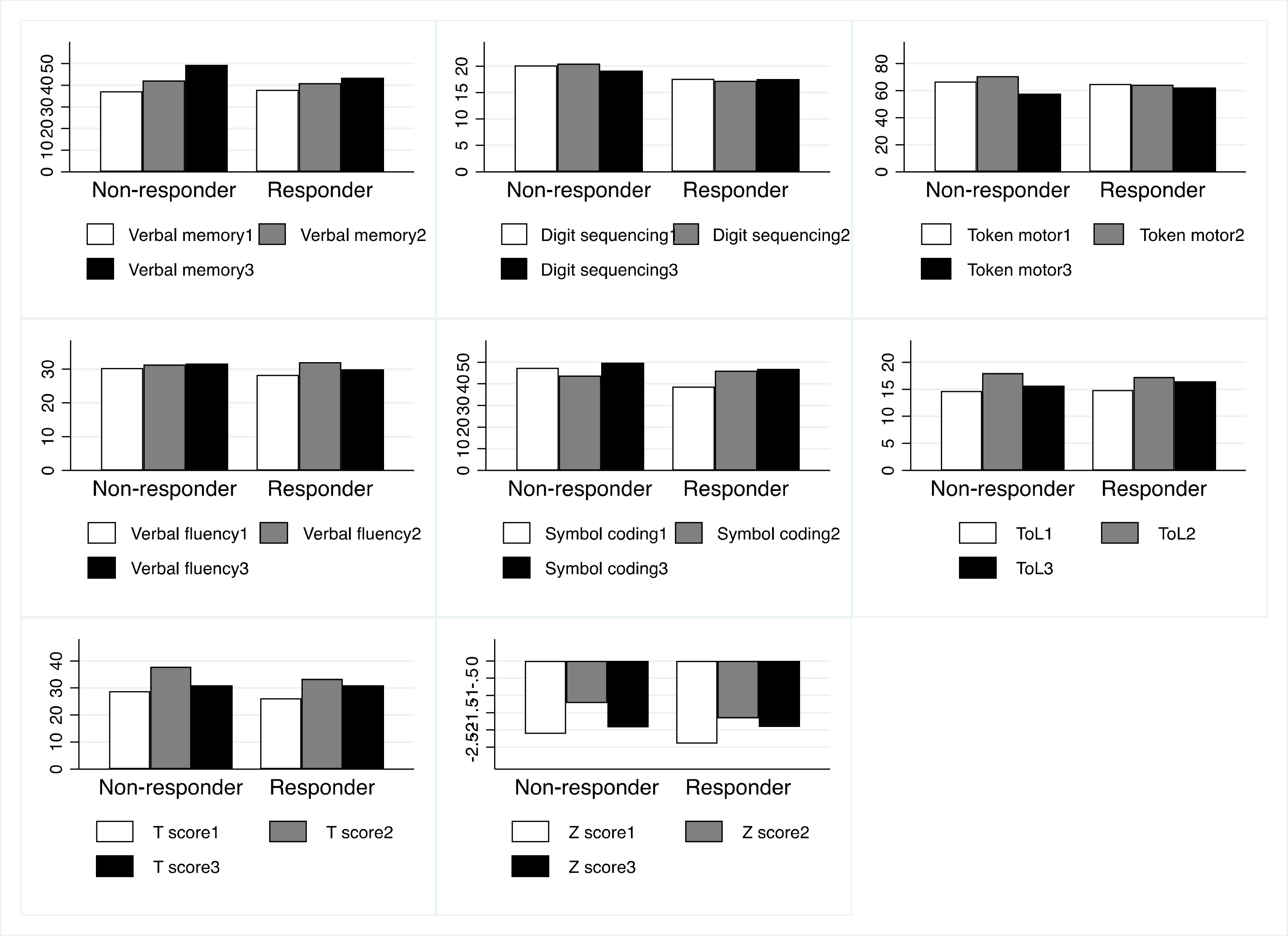

Figure S.1

Bar graphs comparing mean performance on BACS measures between trajectory groups (non-responder vs. responder) at each visit (*white* = baseline, *grey* = 2-week, *black* = 6-week)

Note. *BACS* = Brief Assessment of Cognition in Schizophrenia; *ToL* = Tower of London.

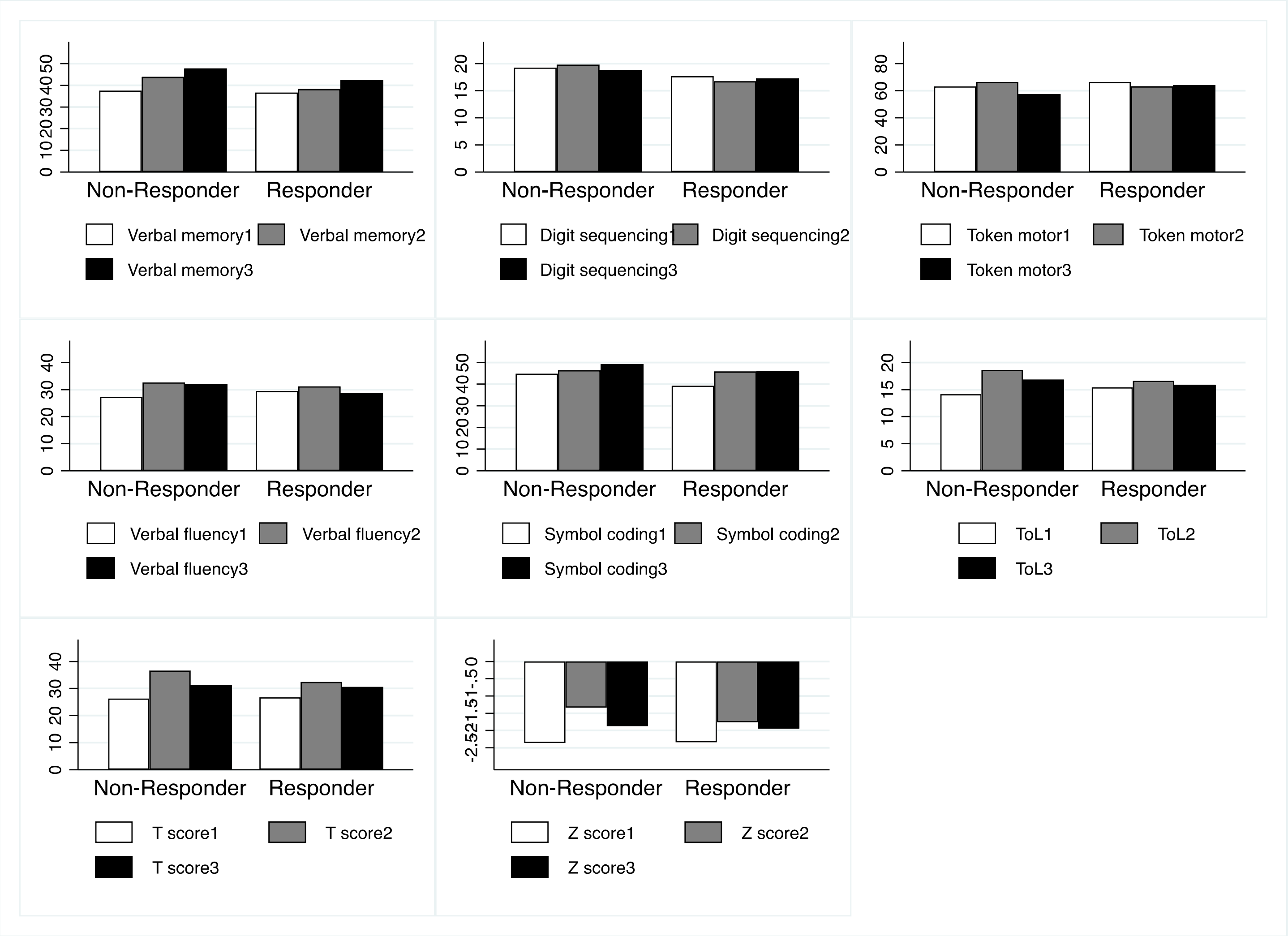

Figure S.2
Bar graphs comparing mean performance on BACS measures using >20% PANSS reduction criteria (non-responder vs. responder) at each visit (*white* = baseline, *grey* = 2-week, *black* = 6-week)
Note. *BACS* = Brief Assessment of Cognition in Schizophrenia; *ToL* = Tower of London.

STROBE Statement—checklist of items that should be included in reports of observational studies

	Item No	Recommendation	Noted on pg.
**Title and abstract**	1	(*a*) Indicate the study's design with a commonly used term in the title or the abstract	1,2,3
		(*b*) Provide in the abstract an informative and balanced summary of what was done and what was found	1,2,3
**Introduction**			
Background/rationale	2	Explain the scientific background and rationale for the investigation being reported	5
Objectives	3	State specific objectives, including any prespecified hypotheses	5
**Methods**			
Study design	4	Present key elements of study design early in the paper	6
Setting	5	Describe the setting, locations, and relevant dates, including periods of recruitment, exposure, follow-up, and data collection	6
Participants	6	(*a*) *Cohort study*—Give the eligibility criteria, and the sources and methods of selection of participants. Describe methods of follow-up<br>*Case-control study*—Give the eligibility criteria, and the sources and methods of case ascertainment and control selection. Give the rationale for the choice of cases and controls<br>*Cross-sectional study*—Give the eligibility criteria, and the sources and methods of selection of participants	6,7
		(*b*) *Cohort study*—For matched studies, give matching criteria and number of exposed and unexposed<br>*Case-control study*—For matched studies, give matching criteria and the number of controls per case	N/A
Variables	7	Clearly define all outcomes, exposures, predictors, potential confounders, and effect modifiers. Give diagnostic criteria, if applicable	7,8
Data sources/ measurement	8*	For each variable of interest, give sources of data and details of methods of assessment (measurement). Describe comparability of assessment methods if there is more than one group	7,8
Bias	9	Describe any efforts to address potential sources of bias	9
Study size	10	Explain how the study size was arrived at	N/A
Quantitative variables	11	Explain how quantitative variables were handled in the analyses. If applicable, describe which groupings were chosen and why	8,9
Statistical methods	12	(*a*) Describe all statistical methods, including those used to control for confounding	8,9
		(*b*) Describe any methods used to examine subgroups and interactions	8,9
		(*c*) Explain how missing data were addressed	N/A
		(*d*) *Cohort study*—If applicable, explain how loss to follow-up was addressed<br>*Case-control study*—If applicable, explain how matching of cases and controls was addressed<br>*Cross-sectional study*—If applicable, describe analytical methods taking account of sampling strategy	N/A
		(*e*) Describe any sensitivity analyses	8,9

For peer review only - http://bmjopen.bmj.com/site/about/guidelines.xhtml

Continued on next page

For peer review only

**Results**			
Participants	13*	(a) Report numbers of individuals at each stage of study—eg numbers potentially eligible, examined for eligibility, confirmed eligible, included in the study, completing follow-up, and analysed	6,7
		(b) Give reasons for non-participation at each stage	7
		(c) Consider use of a flow diagram	N/A
Descriptive data	14*	(a) Give characteristics of study participants (eg demographic, clinical, social) and information on exposures and potential confounders	9,10
		(b) Indicate number of participants with missing data for each variable of interest	N/A
		(c) *Cohort study*—Summarise follow-up time (eg, average and total amount)	N/A
Outcome data	15*	*Cohort study*—Report numbers of outcome events or summary measures over time	11,supplem.
		Case-control study—Report numbers in each exposure category, or summary measures of exposure	N/A
		Cross-sectional study—Report numbers of outcome events or summary measures	N/A
Main results	16	(*a*) Give unadjusted estimates and, if applicable, confounder-adjusted estimates and their precision (eg, 95% confidence interval). Make clear which confounders were adjusted for and why they were included	17, supplem.
		(*b*) Report category boundaries when continuous variables were categorized	N/A
		(*c*) If relevant, consider translating estimates of relative risk into absolute risk for a meaningful time period	17, supplem.
Other analyses	17	Report other analyses done—eg analyses of subgroups and interactions, and sensitivity analyses	13-15, Supplem.
**Discussion**			
Key results	18	Summarise key results with reference to study objectives	18,19
Limitations	19	Discuss limitations of the study, taking into account sources of potential bias or imprecision. Discuss both direction and magnitude of any potential bias	21,22
Interpretation	20	Give a cautious overall interpretation of results considering objectives, limitations, multiplicity of analyses, results from similar studies, and other relevant evidence	20,21
Generalisability	21	Discuss the generalisability (external validity) of the study results	20,21
**Other information**			
Funding	22	Give the source of funding and the role of the funders for the present study and, if applicable, for the original study on which the present article is based	22

*Give information separately for cases and controls in case-control studies and, if applicable, for exposed and unexposed groups in cohort and cross-sectional studies.

Note: An Explanation and Elaboration article discusses each checklist item and gives methodological background and published examples of transparent reporting. The STROBE checklist is best used in conjunction with this article (freely available on the Web sites of PLoS Medicine at http://www.plosmedicine.org/, Annals of Internal Medicine at http://www.annals.org/, and Epidemiology at http://www.epidem.com/). Information on the STROBE Initiative is available at www.strobe-statement.org.

For peer review only

BMJ Open

Cognitive function and treatment response trajectories in first episode schizophrenia: evidence from a prospective cohort study.

Journal:	*BMJ Open*
Manuscript ID	bmjopen-2022-062570.R1
Article Type:	Original research
Date Submitted by the Author:	29-Jul-2022
Complete List of Authors:	Millgate, Edward; King's College London Institute of Psychiatry Psychology and Neuroscience, Department of Psychosis Studies<br>Griffiths, Kira; King's College London Institute of Psychiatry Psychology and Neuroscience, Department of Psychosis Studies<br>Egerton, Alice; King's College London Institute of Psychiatry Psychology and Neuroscience, Department of Psychosis Studies; South London and Maudsley NHS Foundation Trust, NIHR Biomedical Research Centre<br>Kravariti, Eugenia; King's College London Institute of Psychiatry Psychology and Neuroscience, Department of Psychosis Studies; South London and Maudsley NHS Foundation Trust, NIHR Biomedical Research Centre<br>Casetta, Cecilia; King's College London Institute of Psychiatry Psychology and Neuroscience, Department of Psychosis Studies; South London and Maudsley Mental Health NHS Trust, National Psychosis Service<br>Deakin, Bill; University of Manchester, Faculty of Medicine, Biology and Health<br>Drake, Richard; University of Manchester, Division of Psychology & Mental Health; Greater Manchester Mental Health NHS Foundation Trust<br>Howes, Oliver; King's College London Institute of Psychiatry Psychology and Neuroscience, Department of Psychosis Studies; South London and Maudsley NHS Foundation Trust, NIHR Biomedical Research Centre<br>Kassoumeri, Laura; King's College London Institute of Psychiatry Psychology and Neuroscience, Department of Psychosis Studies<br>Khan , Sobia; University of Manchester, Faculty of Medicine, Biology and Health<br>Lankshear, Steve; Greater Manchester Mental Health NHS Foundation Trust<br>Lees, Jane; Greater Manchester Mental Health NHS Foundation Trust; University of Manchester, Faculty of Medicine, Biology and Health<br>Lewis, Shon; University of Manchester; Greater Manchester Mental Health NHS Foundation Trust<br>Mikulskaya, Elena ; University of Manchester, Faculty of Medicine, Biology and Health<br>Oloyede, Ebenezer; King's College London Institute of Psychiatry Psychology and Neuroscience, Department of Psychosis Studies; South London and Maudsley NHS Foundation Trust, Pharmacy Department<br>Owens, Rebecca; University of Manchester, Faculty of Medicine, Biology and Health<br>Pollard, Rebecca; King's College London Institute of Psychiatry

	Psychology and Neuroscience, Department of Psychosis Studies<br>Rich, Nathalie; University College London, Department of Epidemiology & Applied Clinical Research<br>Smart, Sophie; Cardiff University, Division of Psychological Medicine and Clinical Neurosciences<br>Segev , Aviv; King's College London Institute of Psychiatry Psychology and Neuroscience, Department of Psychosis Studies; Shalvata Mental Health Center, Sackler Faculty of Medicine<br>Verena Sendt , Kyra; King's College London Institute of Psychiatry Psychology and Neuroscience, Department of Psychosis Studies<br>MacCabe, James; Institute of Psychiatry Psychology and Neuroscience Division of Academic Psychiatry, Department of Psychosis Studies; South London and Maudsley NHS Foundation Trust, National Psychosis Service
**Primary Subject Heading**:	Mental health
Secondary Subject Heading:	Public health, Patient-centred medicine, Mental health
Keywords:	Schizophrenia & psychotic disorders < PSYCHIATRY, Adult psychiatry < PSYCHIATRY, MENTAL HEALTH

I, the Submitting Author has the right to grant and does grant on behalf of all authors of the Work (as defined in the below author licence), an exclusive licence and/or a non-exclusive licence for contributions from authors who are: i) UK Crown employees; ii) where BMJ has agreed a CC-BY licence shall apply, and/or iii) in accordance with the terms applicable for US Federal Government officers or employees acting as part of their official duties; on a worldwide, perpetual, irrevocable, royalty-free basis to BMJ Publishing Group Ltd ("BMJ") its licensees and where the relevant Journal is co-owned by BMJ to the co-owners of the Journal, to publish the Work in this journal and any other BMJ products and to exploit all rights, as set out in our licence.

The Submitting Author accepts and understands that any supply made under these terms is made by BMJ to the Submitting Author unless you are acting as an employee on behalf of your employer or a postgraduate student of an affiliated institution which is paying any applicable article publishing charge ("APC") for Open Access articles. Where the Submitting Author wishes to make the Work available on an Open Access basis (and intends to pay the relevant APC), the terms of reuse of such Open Access shall be governed by a Creative Commons licence – details of these licences and which Creative Commons licence will apply to this Work are set out in our licence referred to above.

Other than as permitted in any relevant BMJ Author's Self Archiving Policies, I confirm this Work has not been accepted for publication elsewhere, is not being considered for publication elsewhere and does not duplicate material already published. I confirm all authors consent to publication of this Work and authorise the granting of this licence.

For peer review only

Journal: BMJ Open

Abstract word count: 310 (288 without headings)

Word count: 4345

Original submission date: 4th March 2022

Submission date following corrections: 18th July 2022

Cognitive function and treatment response trajectories in first episode schizophrenia: evidence from a prospective cohort study.

Edward Millgate[1], Kira Griffiths[1], Alice Egerton[1,2], Eugenia Kravariti[1,2], Cecilia Casetta[1,3], JFW Deakin[4,5], Richard J Drake[4,5,6], Oliver D Howes[1,2], Laura Kassoumeri[1], Sobia Khan[4], Steve Lankshear[5], Jane Lees[4,5,6], Shôn Lewis[4,5,6], Elena Mikulskaya[4], Ebenezer Oloyede[1,7], Rebecca Owens[4], Rebecca Pollard[1], Nathalie Rich[8], Sophie E Smart[9], Aviv Segev[1,10,11], Kyra-Verena Sendt[1], The STRATA Consortium, James H MacCabe[1,3]*

[1] Department of Psychosis Studies, Institute of Psychiatry, Psychology and Neuroscience, King's College London, UK

[2] NIHR Biomedical Research Centre at South London and Maudsley NHS Foundation Trust, London, UK

[3] National Psychosis Service, South London and Maudsley NHS Foundation Trust, London, UK

[4] Division of Psychology and Mental Health, School of Biological Sciences, Faculty of Biology, Medicine and Health, University of Manchester, Manchester, UK

[5] Greater Manchester Mental Health NHS Foundation Trust, Manchester, UK

[6] Manchester Academic Health Science Centre, Manchester, UK

[7] Pharmacy Department, South London and Maudsley NHS Foundation Trust, UK

[8] Department of Epidemiology & Applied Clinical Research, University College London, UK

[9] MRC Centre for Neuropsychiatric Genetics and Genomics, Division of Psychological Medicine and Clinical Neurosciences, Cardiff University, Cardiff, UK

[10] Shalvata Mental Health Centre, Israel

[11] Sackler Faculty of Medicine, Tel Aviv University, Israel

*Corresponding author: Edward.millgate@kcl.ac.uk

For peer review only

Department of Psychosis Studies,

Institute of Psychiatry, Psychology & Neuroscience,

16 de Crespigny Park,

Denmark Hill, London,

United Kingdom, SE5 8AF

For peer review only

Abstract

Objectives: This prospective cohort study tested for associations between baseline cognitive performance in individuals early within their first episode and antipsychotic treatment of psychosis. We hypothesised that poorer cognitive functioning at the initial assessment would be associated with poorer antipsychotic response following the subsequent six weeks.

Setting: NHS service users with a first episode schizophrenia diagnosis, recently starting antipsychotic medication, recruited from two UK sites (King's College London, UK & University of Manchester, UK). Participants attended three study visits following screening.

Participants: Eighty-nine participants were recruited, with 46 included in the main analysis. Participants required to be within the first two years of illness onset, had received minimal antipsychotic treatment, have the capacity to provide consent, and be able to read and write in English. Participants were excluded if they met remission criteria or showed mild to no symptoms.

Primary and secondary outcomes: Antipsychotic response was determined at 6-weeks using the Positive and Negative Syndrome Scale (PANSS), with cognitive performance assessed at each visit using the Brief Assessment of Cognition in Schizophrenia (BACS). The groups identified (responders and non-responders) from trajectory analyses, as well as from >20% PANSS criteria, were compared on baseline BACS performance.

Results: Trajectory analyses identified 84.78% of the sample as treatment responsive, and the remaining 15.22% as treatment non-responsive. Unadjusted and adjusted logistic regressions observed no significant relationship between baseline BACS on subscale and total performance (BACS t-score: OR = 0.98, $p = .620$, Cohen's d = .218) and antipsychotic response at 6-weeks.

Conclusions: This investigation identified two clear trajectories of treatment response in the first six weeks of antipsychotic treatment. Responder and non-responder groups did not significantly differ on performance on the BACS, suggesting that larger samples may be required or that an association between cognitive performance and antipsychotic response is not observable in the first two-years of illness onset.

Trial registration number: REC: 17/NI/0209

Keywords: cognition, antipsychotic response, BACS, trajectories, first episode schizophrenia

For peer review only

Article summary

Strengths and limitations of this study

- The study examined baseline cognitive performance in a sample of first-episode schizophrenia, with patients recently starting antipsychotic treatment.
- Trajectory analyses identified two clear patterns of antipsychotic response at 6-weeks after baseline assessment.
- The lack of significant group differences in baseline cognitive performance between antipsychotic responders and non-responders may be a result of under-sampled comparisons or that brief cognitive batteries for schizophrenia may not be a useful predictor of response in the first two-years of illness onset.

For peer review only

Introduction

Prompt intervention with pharmacological therapy for individuals with schizophrenia has been extensively recommended in the literature[1,2] and is reported to be associated with better functional outcomes[3,4,5]. As observed by Carbon & Correll[5], a lack of early response and improvement to antipsychotic medication is a strong predictor of later non-response. A recent diagnostic test review has even argued that non/minimal response to antipsychotic medication in the first 2 weeks of treatment may be a sufficient indication to switch antipsychotic[6]. Early and accurate detection of treatment non-responders at first episode is also more likely to result in timely treatment with clozapine, which may be associated with better outcomes[7]. Indeed, Yoshimura et al[8] found that response to clozapine was ~80% in treatment resistant patients who were commenced on clozapine early in their illness course, with this depreciating to ~30% when clozapine initiation was delayed by more than 2.8years[7,8].

Individuals who do not respond to antipsychotic medication are reported to have higher rates of smoking (56%), substance and alcohol abuse (51%) and suicidal ideation (44%), with annual treatment costs being 3 to 11 times larger than those who respond to antipsychotic medication[9]. In 2007, it was estimated that schizophrenia accounted for 30% of the total expenditure for adult mental health and social care services[10], with additional economic and societal costs due to unemployment or absence from work. These total service costs, which were estimated at £2.2 billion in 2007, have the potential to reach £3.7 billion by 2026[11]. However, it has been suggested that early intervention programmes could aid in reducing these costs substantially if adequately introduced in first episode psychosis[12], as earlier onset schizophrenia is associated with greater expected costs[11].

Early cognitive deficits may be predictive of subsequent antipsychotic response in the first episode of illness and could aid in delivering fast, early, intervention. Cognitive

For peer review only

dysfunction in schizophrenia is observable prior to illness onset[13,14] and is strongly associated with poorer functional outcomes[15,16,17]. A recent meta-analysis comparing cognitive performance in known cases of antipsychotic treatment resistance and response[18] observed worse performance in treatment resistant samples across cognitive domains, with the strongest effect in measures of verbal memory and learning and language functions. However, it is possible that illness chronicity and exposure to long-term antipsychotic treatment may have influenced these findings.

Based on the current existing literature it is plausible to argue that there may be quantifiable cognitive differences between individuals who respond to antipsychotic medication and those who do not in the early stages of the illness; seeing as deficits in cognition are observable prior to illness onset[14,15] and poor early non-response to medication being predictive of future non-response[5]. Therefore if differences are observed between groups of differing response to medication (i.e. responders and non-responders), early in their illness and treatment, this will broaden our understanding of the relationship between cognition, schizophrenia, and antipsychotic response, as well as aid clinical utility by using brief cognitive measures as a screening for potential non-response in the first episode of schizophrenia. The American Psychological Association's Working Group on Screening and Assessment have provided guidelines for determining the appropriateness of a neuropsychological measure for cognitive screening within a clinical setting[19]. The guidelines are as follows: i. provide identification for those at high risk for impairment, ii. sensitive enough to identify those who need further review, iii. brief and narrow in scope, iv. can be administered at routine visits, v. can be administered by support staff or clinicians electronically and vi. can be used to monitor progress and outcomes[20]. In high-income countries, the use of brief assessment batteries such as the BACS have been found to meet these criteria put forward by the APA working group[21].

For peer review only

Therefore, this prospective cohort study tested for associations between baseline cognitive performance using a brief cognitive battery, assessed at the initiation of antipsychotic treatment, in individuals early within their first episode of psychosis and their subsequent response to antipsychotic treatment. We hypothesised that poorer cognitive functioning at the initial assessment would be associated with poorer response over the subsequent six weeks of antipsychotic treatment.

Methods

Design

The study used a prospective cohort design with a sample of patients with first-episode schizophrenia. Participants were assessed over a period of 6-weeks, with two follow-up visits following baseline and screening assessments.

Setting

The study was part of the 'Schizophrenia: Treatment Resistance and Therapeutic Advances' (STRATA) consortium which included two UK sites in this study; King's College London (London, UK) and University of Manchester (Manchester, UK). The aim of the STRATA consortium is to identify neurobiological, cognitive, and genetic biomarkers of antipsychotic treatment resistance and non-response within schizophrenia and other related psychotic disorders.

Patient and Public Involvement

In the early development and design of the study consultations with the NIHR Maudsley Biomedical Research Centre (BRC) Service User Advisory Group (SUAG) took place to determine the feasibility of the study and its' assessments for service users. The NIHR Maudsley BRC Feasibility and Acceptability Support Team for Researchers (FAST-R) service was also used in order to receive feedback on consent forms, information sheets and protocols, as well as advice for recruitment strategies for service users.

Participants

For peer review only

89 participants aged between 18 – 65 years with a DSM-5 diagnosis of schizophrenia, schizoaffective, schizophreniform disorder, or psychosis (non-specified) (ICD-10: F20-F29) were recruited across two UK sites (King's College London and University of Manchester). Inclusion required that participants were within the first two years of illness onset, defined using the date of first initial contact with services and clinical records. Inclusion also required that participants had received minimal antipsychotic medication, which was defined as having received antipsychotic treatment for no longer than 4 weeks prior to the baseline visit, after a period of being either antipsychotic naïve or antipsychotic-free for at least 14 days. Participants were assessed at baseline within the first 2 weeks of antipsychotic medication initiation. Participants also were required to have the capacity to provide consent and the ability to read and write in English. Participants were excluded if they met modified Andreasen remission criteria[22], having mild or less scores on all of the following Structured Clinical Interview-Positive and Negative Syndrome Scale (SCI-PANSS)[23] items: delusions (P1), conceptual disorganisation (P2), hallucinatory behaviour (P3), blunted affect (N1), social withdrawal (N4), lack of spontaneity (N6), mannerisms/posturing (G5), unusual thought content (G9) on the day of assessment, as this would suggest that their symptoms were in remission. Participants were also required to show adherence to medication, as evidence by a Kemp Clinician Rating Scale (CRS)[24,25] of equal to or greater than 3 (*"Accepts only because compulsory, or very reluctant / requires persuasion, or questions the need for medication often*").

Participants were assessed within the first 14 days of starting antipsychotic medication at baseline, 2-weeks from baseline assessment (±7days of date) and 6-weeks from baseline assessment (±7 days of date), with the maximum cut-off for 6-week follow up being 56 days after baseline assessment (i.e. if an individual was assessed at the maximum follow-up periods at 2-week and 6-week visits; 8-weeks total). Participants were reimbursed for their time and expenses for participation in the study. Fourteen participants were withdrawn after providing consent, an additional 20 were withdrawn following baseline, and another 9 participants withdrawn following 2-week assessment. Participants were withdrawn if they were unable to attend the study visit, their symptoms were in remission (as per Andreasen

For peer review only

remission criteria[22]), or if they no longer wanted to take part in the study and requested to have their data removed (see Figure 1). 46 participants were eligible for inclusion in the analysis. All participants gave informed consent prior to enrolment. This study was approved by the Health and Social Care Research Ethics Committee A; REC: 17/NI/0209. All participants provided informed consent prior to participation.

[Figure 1; CONSORT]

Definitions for treatment response status

Treatment response groups were modelled through trajectory analyses using the *traj* command in STATA[26]. This tool estimates group-based trajectories over a specified time interval, clustering individuals who follow similar trajectories through a censored normal model. Akaike information criterion (AIC) and Bayesian information criterion (BIC) values were used to select the trajectory model with the lowest AIC and BIC values. Linear trajectories of up to five classes (1 to 5 trajectories) were assessed for eligibility. Rescaled PANSS scores[27] were calculated by subtracting 30 from total scores prior to producing estimates for percentage change at weeks 2 and 6 were used in the model. The results generated using this trajectory grouping were also compared to a more standard definition of treatment response which uses a >20% reduction in rescaled PANSS total scores from initial to final assessment[28,29]. Here patients not reaching a 20% reduction in rescaled PANSS total scores at the 6-week visits were categorised as non-responsive. These results are reported in the supplementary material.

For peer review only

Materials

Clinical and demographic measures

At baseline, participants completed the Kemp Clinician Rating Scale (CRS)[24,25], Mini-international neuropsychiatric interview (MINI)[30] (M-Psychotic Disorders; A-Major Depressive Episode; D-Manic/Hypomanic/Bipolar), Structured Clinical Interview- Positive and Negative Syndrome Scale (SCI-PANSS)[23], Clinician Rating Scale for Schizophrenia (CGI-SCH)[31] and provided demographic data. At each subsequent study visit the CRS, SCI-PANSS and CGI-SCH were repeated.

Neuropsychological assessment

Participants completed Version A of the Brief Assessment of Cognition in Schizophrenia (BACS)[32] at each study visit. The BACS was originally developed to assess cognitive functioning in schizophrenia, while also being an easily administrable and brief test battery[32]. The battery includes tasks pertaining to executive functions, working memory, motor/processing speed, verbal memory, verbal fluency, and attention cognitive domains. Version A includes the following tasks: i) list learning task (verbal memory); ii) digit sequencing task (working memory); iii) token motor task (motor speed); iv) category instances task (verbal fluency); v) symbol coding task (attention and speed of information processing) and vi) tower of London (executive function). All tasks on the BACS are scored such that higher scores represent better performance. Composite t and z scores for the BACS were generated using scores from published normative data[33].

Data analysis

All analyses were conducted in STATA 15/SE[34]. Wilcoxon signed rank tests were used to compare cognitive performance and symptom severity in the whole sample between visits (i.e. baseline assessment to 2-week, 2-week to 6-week, and baseline to 6-week) as not all symptom severity and cognitive variables were normally distributed. Baseline cognitive performance on the BACS was compared between trajectory groups using multivariable logistic regressions on the BACS composite and subscale scores. All models were adjusted for age, gender, and days from 1st psychotic symptom to baseline antipsychotic medication (i.e. duration of untreated psychosis; DUP). Results were then compared to groupings based on >20% reduction in rescaled PANSS total scores[27,28] from baseline assessment to 6-week follow-up (supplementary material Table S.1).

Finally, growth curve models were executed using the *xtmixed* command[35] to compare cognitive performance over time between trajectory groups to estimate any changes in cognitive performance over the study period (supplementary material Table S.2). These results were again compared to >20% PANSS reduction criteria for treatment response (supplementary material Table S.3).

Results

Table 1 reports the demographic descriptive of the whole sample included in analysis (N = 46) at baseline, with PANSS symptom severity scores and BACS performance for each study visit illustrated in Table 2. Data regarding antipsychotic medication was provided by all participants at baseline, all of which were treated with 2nd generation antipsychotics. At baseline 45 participants provided PANSS symptom severity ratings, with 41 providing at least one baseline measure of the BACS (Table 2). Between baseline and 2-week assessment the average follow-up was 18.19 days (SD = 6.6) and between 2-week and 6-week this was 26.69 days (SD = 9.6). Between baseline and 6-week visit, the study trial lasted 43.86 days (SD = 7.2).

For peer review only

Between study visits, a significant improvement in PANSS positive symptoms scores was observed in the whole sample between baseline and 2-week visits, 2-week and 6-week visits, as well as baseline and 6-week assessments (Table 2). A significant improvement in PANSS total scores was observed between baseline and 2-week and baseline and 6-week visits. No significant differences in symptom severity were observed between visits for negative symptoms (Table 2). In the whole sample, cognitive performance on the BACS verbal memory significantly improved between baseline and 2-week visits, 2-week and 6-week visits, as well as baseline and 6-week assessments (Table 2). Verbal fluency significantly improved between baseline and 2-week visits. Symbol coding, Tower of London, and overall (tscore and zscore) performance improved significantly between baseline and 2-week visits and baseline and 6-week visits (Table 2).

Table 1

Descriptive statistics of the whole sample demographics at consent (age) and baseline assessments

Variable	N	M	SD	Min	Max
Age (at consent)	46	27.30	8.17	18	50
Gender (*male*)	33 (71.74%)	-	-	-	-
Gender (*female*)	13	-	-	-	-

	(28.26%)				
Age of illness onset (years)	46	26.53	8.45	18	49
Duration from 1st Psychotic symptom (days) to baseline antipsychotic (DUP)	46	248.30	245.06	0	726
Duration from 1st contact with mental health services (days) to baseline antipsychotic	46	346.57	600.37	6	2358
Chlorpromazine equivalents (mg/day)	46	176.89	121.29	10	800
Number of hospitalisations	46	0.89	0.64	0	3
Years of education	42	13.62	2.82	5	20
CGI-SCH baseline score	56	Minimally ill = 1<br>Mildly ill = 4<br>Moderately ill = 12<br>Markedly ill = 15<br>Severely ill = 12<br>Among the most severely ill = 1	-	-	-
Antipsychotic medication	51	Amisulpride = 1<br>Aripiprazole = 19<br>Olanzapine = 16<br>Paliperidone = 1<br>Quetiapine = 4<br>Risperidone = 5	-	-	-

Note. *DUP* = Duration of untreated psychosis; *CGI-SCH* = Clinician Rating Scale for Schizophrenia.

For peer review only

Table 2

Mean symptom severity as rated by the PANSS and BACS performance for the whole sample for each study visit

	Baseline			2-week follow-up			6-week follow-up			Baseline vs 2-week	2 -week vs 6-week	Baseline vs 6-week
Variable	N	M	SD	N	M	SD	N	M	SD	Wilcoxon signed-rank	Wilcoxon signed-rank	Wilcoxon signed-rank
PANSS positive	45	11.93	4.77	36	9.17	5.36	38	7.26	5.13	Z = 2.76, *p* = .006*	Z = 2.67, *p* = .008*	Z = 4.50, *p* <.001*
PANSS negative	45	10.36	6.87	36	10.31	7.15	38	9.58	7.78	Z = 0.78, *p* = .435	Z = 0.62, *p* = .535	Z = 1.17, *p* = .242
PANSS general	45	19.40	8.57	36	16.64	10.60	38	15.74	10.11	Z = 3.21, *p* = .001*	Z = 2.48, *p* = .013*	Z = 0.64, *p* = .524
PANSS total	45	41.69	16.11	36	36.11	20.03	38	32.58	20.04	Z = 3.10, *p* = .002*	Z = 1.46, *p* = .144	Z = 3.35, *p* < .001*
BACS Verbal memory	41	37.83	14.02	32	41.16	13.50	36	44.56	15.02	Z = -3.14, *p* = .002*	Z = -3.15, *p* = .002*	Z = -3.88, *p* <.001*
BACS Digit sequencing	38	18.03	4.06	32	17.84	4.33	34	17.85	4.69	Z = -0.78, *p* = .433	Z = -0.40, *p* = .688	Z = 0.40, *p* = .687
BACS Verbal fluency	42	28.60	7.85	31	31.87	9.47	35	30.20	8.38	Z = -1.96, *p* = .050*	Z = 0.83, *p* = .405	Z = -1.62, *p* = .105
BACS Token motor	39	65.36	10.83	32	65.56	17.81	34	61.38	20.94	Z = -1.30, *p* = .193	Z = -0.55, *p* = .583	Z = -0.24, *p* = .812
BACS Symbol coding	39	40.15	13.13	32	45.69	12.56	32	47.25	11.96	Z = -2.25, *p* = .025*	Z = -1.07, *p* = .284	Z = -3.29, *p* = .001*

For peer review only

BACS ToL	37	14.84	4.48	29	17.38	3.29	32	16.28	4.13	Z = -3.24, *p* = .001*	Z = 1.45, *p* = .148	Z = -2.42, *p* = .016*
BACS t-score	33	26.67	11.98	28	34.14	11.68	30	30.87	14.95	Z = -3.79, *p* < .001*	Z = -0.29, *p* = .769	Z = -3.66, *p* <.001*
BACS z-score	33	-2.34	1.19	28	-1.59	1.17	30	-1.91	1.50	Z = -3.85, *p* <.001*	Z = -0.23, *p* = .820	Z = -3.67, *p* <.001*

Note. *PANSS* = Positive and Negative Symptom Scale; *BACS* =Brief Assessment of Cognition in Schizophrenia; *ToL* = Tower of London.
* = significant at *p* = .05 level.

For peer review only

Trajectories of symptom change

BIC and AIC values were generated for five classes of trajectory models (Table 3). Of these both indices indicate that the two-trajectory group is best fitted to the data. This model estimated 73.7% of the sampled population to be from one linear trajectory, with the remaining 26.3% in another.

Table 3
Selecting a trajectory model using BIC and AIC estimates

No. of classes	1	2	3	4	5
BIC	-522.21	-512.13	-517.87	-520.14	-525.88
AIC	-519.46	-506.64	-509.64	-509.17	-512.17
%. in each class	100%	73.7% ; 26.3%	73.7% ; 26.3%; 0%	60.7% ; 23.7%; 15.6%; 0%	60.7% ; 23.7%; 15.6%; 0%; 0%

Note. *BIC* = Bayesian Information Criteria; *AIC* = Akaike's Information Criteria.

The trajectories identified by the *traj* procedure are shown in Figure 2. The trajectories that emerged clearly represented responders versus non-responders. 39 participants (84.78%) of the sample were classified as antipsychotic treatment responsive and 7 as treatment non-responsive (15.22%). For responders, PANSS total score percentage change at 6 weeks was on average 32.89% (SD = 27.5) symptom improvement. For non-responders this was -21.03% (SD = 16.1) indicating a minimal and, in some cases, worsening in symptom severity. Shape estimates and standard errors of antipsychotic response are shown in Table 4. Treatment responders significantly improved over the 6-week period. Descriptive statistics of clinical and demographic variables between both trajectory groups (non-responder; responder) are presented in the supplementary material (Table S.4.) In comparison to those responsive to antipsychotic medication, non-responders were on average older, had a longer duration of time from first contact with mental health services to baseline antipsychotic medication, had marginally more hospitalisations, attained more years of education, and were treated at higher chlorpromazine equivalents (supplementary material Table S.4).

For peer review only

[Figure 2]

Table 4

Parameter estimates and standard errors for both trajectories of antipsychotic response

Parameters	Trajectories	
	Non-responder (N = 7)	Responder (N = 39)
Intercept	2.54	-3.71
Linear change	0.10	-0.54
Standard error	0.06	0.09
T statistic	1.61, $p = .111$	-6.06, $p < .001$

Cognitive performance

There was a significant improvement in BACS verbal memory and symbol coding performance between baseline and 6-week assessments across the whole sample, with a significant improvement in Tower of London and BACS z and t composite scores between baseline and 2-week visits (Table 2). At baseline assessment, there was no difference in the BACS subscale or composite scores between antipsychotic responders and non-responders identified in the trajectory analysis (Table 5; Table 6). Growth curve models observed no significant change in cognitive performance over follow-up visits between trajectory groups (supplementary material Figure S.1, Table S.2). A similar pattern in results was observed when >20% PANSS reduction criteria was applied (supplementary material Figure S.2, Table S.3, Table S.5).

For peer review only

Table 5

Baseline cognitive performance for both trajectory groups

	Non-responder			Responder		
BACS measure	N	Mean	SD	N	Mean	SD
Verbal Memory	7	37.29	9.48	34	37.94	14.89
Digit Sequencing	6	20.17	5.38	32	17.63	3.74
Verbal Fluency	7	30.29	7.30	35	28.26	8.01
Token Motor	7	66.86	8.93	32	65.03	11.30
Symbol Coding	6	47.50	6.35	33	38.82	13.66
Tower of London	7	14.71	3.77	30	14.87	4.69
tscore composite	6	28.83	14.36	27	26.19	11.65
zscore composite	6	-2.12	1.41	27	-2.39	1.16

Note. *BACS* =Brief Assessment of Cognition in Schizophrenia.

Multivariable linear regression

Univariable and multivariable logistic regression models adjusting for age and gender and duration of untreated psychosis (DUP) found no significant associations between BACS performance at baseline and response trajectory over 6 weeks (Table 6), with no association of any demographic or clinical variables in multivariable models. This was also observed when utilising the >20% reduction in PANSS total criteria (supplementary material Table S.4).

For peer review only

Table 6

Results from univariable and multivariable logistic regression models for response status and baseline BACS performance

	Unadjusted					Adjusted for age, gender, and DUP				
BACS task	β	SE	95%CI	OR	P-value	β	SE	95%CI	OR	P-value
Verbal Memory	<0.01	0.03	-0.06 ; 0.06	1.00	.909	<-0.01	0.03	-0.07 ; 0.06	1.00	.918
Digit Sequencing	-0.17	0.12	-0.41 ; 0.07	0.84	.168	-0.18	0.13	-0.44 ; 0.07	0.83	.151
Verbal Fluency	-0.03	0.05	-0.14 ; 0.07	0.97	.530	-0.05	0.06	-0.17 ; 0.07	0.95	.417
Token Motor	-0.02	0.04	-0.09 ; 0.06	0.98	.683	-.0.02	0.05	-0.11 ; 0.08	0.99	.737
Symbol Coding	-0.06	0.04	-0.14 ; 0.02	0.94	.145	-0.07	0.05	-0.16 ; 0.02	0.93	.114
Tower of London	0.08	0.09	-0.18 ; 0.19	1.01	.935	-0.01	0.10	-0.20 ; 0.19	0.99	.947
t score composite	-0.02	0.04	-0.10 ; 0.06	0.98	.620	-0.02	0.04	-0.10 ; 0.06	0.98	.594
z score composite	-0.21	0.40	-0.99 ;0.58	0.81	.603	-0.23	0.41	-1.02 ;0.57	0.80	.573

Note. BACS = Brief Assessment of Cognition in Schizophrenia; *CIs* = confidence intervals; *DUP* = duration of untreated psychosis.

For peer review only

For peer review only

Discussion

This prospective study investigated the relationship between baseline cognitive performance and subsequent antipsychotic response over a 6-week treatment period. Across the whole sample, participants showed an overall reduction in symptom severity as well as an improvement in cognitive performance on the majority of BACS tasks. Trajectory analyses estimated two trajectory groups (73.7%, 26.3%) based on PANSS total % change from baseline; this was reflected as 84.78% of the sample being grouped as treatment responsive, and the remaining 15.22% as treatment non-responsive. Contrary to our hypothesis, baseline cognitive performance did not significantly differ between those identified as treatment responders or non-responders following 6 weeks of antipsychotic treatment. This finding remained the same when treatment response was defined as at least a 20% reduction in PANSS total scores, suggesting that there is no association between cognitive performance and antipsychotic response in first episode schizophrenia.

Across the 2-week and 6-week follow-up visits, an improvement in cognitive performance was observed for the whole sample on BACS measures of verbal memory, verbal fluency, symbol coding and Tower of London tasks, as well as the BACS composite scores. Most of these changes occurred between baseline and 2-week assessment (Table 2), with small decreases in performance on measures of verbal fluency, token motor and Tower of London tasks between 2-week and 6-week assessments, as well as for composite z and t scores. In contrast, there was a decline in performance in the token motor task across the follow-up period, and minimal changes in performance in the digit sequencing task.

The observed improvement in cognitive performance may reflect a beneficial outcome of antipsychotic treatment. 1st generation antipsychotics, introduced in the 1950s, target the positive symptoms observed in schizophrenia by acting as an antagonist at dopamine D2 receptors. Treatment with this group of antipsychotic drugs has been associated with motor and cognitive deficits in patients[36,37]. In contrast, 2nd generation antipsychotics are reported to have fewer extrapyramidal side effects[38], with these drugs also acting as an antagonist at the serotonin 5HT2A receptor, in addition to D2 dopamine receptors. Research suggests that in comparison to 1st generation, 2nd generation antipsychotics can provide some improvement in cognitive performance (e.g. clozapine[39]). Guilera et al (2009)[40] found in their meta-analysis of 18 randomised controlled trials that 2nd generation drugs provided a

For peer review only

slight improvement in performance for global cognition, as well as slight but significant improvements in measures of procedural learning, language and verbal comprehension, verbal learning and memory and visual learning and memory.

As the whole study sample was treated with 2nd generation antipsychotic drugs at baseline assessment (Amisulpride = 1, Aripiprazole = 19, Olanzapine = 16, Paliperidone = 1, Quetiapine = 4 and Risperidone = 5), it is possible that the improvement in cognitive performance observed in our sample may be a result of 2nd antipsychotic treatment effects, although 1st generation antipsychotic use could not be compared. However it has also been argued that improvements in cognitive performance over longitudinal designs may instead reflect practice effects (e.g. familiarity and procedural learning[41]), meaning that improvement in cognitive performance in our sample could also be attributable to practice effects between study visits. Lees et al[42] estimated the magnitude of these effects using both the MATRICS Consensus Cognitive Battery (MCCB)[43] and the Cog State Schizophrenia Battery[44], finding strong test-retest correlations between repeated baseline visits across cognitive batteries, with potential learning effects in social-emotional cognition. However, the authors also observed that participants may have failed to complete the initial baseline assessment due to difficulty in understanding the task, with the suggestion that future investigations using either battery would benefit from adopting initial practice sessions to reduce practice effects. Therefore an initial practice session with the BACS may have reduced the size of improvement observed in cognitive performance from baseline performance. Another way to determine the extent of practice effects in our sample would be to have a control group who is already stable on antipsychotic medication to see if similar outcomes are observed between groups.

Despite all of the sample being treated with 2nd generation antipsychotics, it is also possible that some anticholinergic effects, which differ between 2nd generation antipsychotic drugs[45], may have affected cognitive performance. Long term exposure to antipsychotic medications of high anticholinergic activity have been previously reported to impact cognitive performance in patient samples[46,47,48]. Using low and high anticholinergic activity criteria from a recent review comparing medication effects (from Stroup & Gray[49]; refer to Table 1, pg. 342), our sample had 44% (N = 20) treated with a high anticholinergic antipsychotic, meaning that the absence of significant differences between groups may have been a result of heterogeneity in medication effects. Therefore, future investigations should

For peer review only - http://bmjopen.bmj.com/site/about/guidelines.xhtml

consider the role of antipsychotic treatment effects on cognitive outcomes within schizophrenia.

Trajectory analyses identified two clearly defined trajectories of treatment response, both of which are consistent across both timepoints: one trajectory showing good response, and one of little to no response (Figure 2). Confidence intervals (Figure 2) show some overlap between trajectories in the first ~20 days since baseline assessment, with these becoming independent following this period, meaning that separation between trajectory groups was apparent at around 3 weeks. This supports the findings from Samara et al[6] who found poor/minimal response to antipsychotic treatment at 2-weeks to be predictive of future treatment non-response. In previous investigations using first episode samples, 4 or more trajectories have been identified[50,51]. However, both these investigations used longer periods of follow-up as well as raw un-adjusted PANSS scores in their analyses: as the minimum raw score of the PANSS is 30, it is recommended to rescale the scores by subtracting 30 from total scores prior to producing percentages and ratios[52]. Therefore building trajectory models using raw scores may not be appropriate to use as ratio operations (e.g. calculating proportions and percentages) require a natural zero point[52].

Growth curve models which were used to quantify change in cognitive performance between trajectory groups observed no significant changes in performance between visits. It is possible that this may be due to under sampled groups, as significant improvements for verbal memory, symbol coding, Tower of London and composite scores were observed in the whole sample. When comparing our findings to a >20% reduction in rescaled PANSS total scores criteria[24,25] there were no changes in the pattern of results to growth curve models or logistic regression outcomes. Using this criterion for treatment response resulted in a more even distribution of the total sample to groups (responder = 17 ; non-responder = 21), providing more power to comparative analyses. However despite this there was no change in the pattern of results, meaning that this criterion provided no added benefit to this analysis over trajectory-based groupings. The lack of significant difference in baseline cognition between those classified as treatment responders or non-responders after 6 weeks of treatment in our study contrasts previous research conducted which observed impaired cognitive performance in the poor response trajectory at week 4, with good performance at baseline being predictive of a good response trajectory at week 4[51]. Likewise, longitudinal research using the MCCB[43] with first-episode schizophrenia patients assessed at baseline

For peer review only

and at a 12-week follow up identified tasks of executive function and planning and reasoning ability as potential indices of antipsychotic response[53], with similar findings observed when cognitive performance is correlated with symptom severity measures[54].

Limitations

Previous investigations included sample sizes several magnitudes higher than in our study (Levine & Rabinowitz[51]; N = 491; Trampush et al[53]; N = 175) and it is likely that our sample size limited our ability to observe a significant relationship between cognitive performance and antipsychotic response. Using our sample's mean values for the BACS t composite score, a power calculation found that a total sample size of 31,304 samples would be required to detect a significant difference between trajectory groups at 90% power. When using the >20% PANSS reduction criteria this was N = 6,118, suggesting that both analyses were underpowered due to under sampling.

Another considerable limitation of the conclusions from this investigation is the expectation of detecting meaningful change in both clinical response to medication and cognition in such a short duration of follow-up. Previous longitudinal investigations into cognitive change have noted that even a period of 1 to 3 years may not be substantial to detect changes in cognitive performance[55] , questioning the additional analyses in this study comparing performance between baseline and 2-week and 6-week study visits. Likewise, Emsley et al's[56] investigation with 522 participants with first episode schizophrenia found 11.2% of their sample to not achieve clinical response (determined by a 20% improvement in PANSS total scores) until after 8 weeks, with the authors concluding that antipsychotic response is greatly varied and that longer investigations are needed to capture the large variability in clinical response[56]. Therefore it is also possible that there are participants within the sample who may have later responded to medication if the follow-up was at a longer duration, which may also partially support the lack of significant differences between groups in this study. Likewise, adopting secondary criteria for treatment response and non-response based off criteria from the TRIPP Working Group[57] would also help in seeing whether the groupings identified by trajectory analyses correspond to standardised guidelines, aiding in comparison between investigations.

Due to the issues with small sample sizes, it was not possible to adjust for additional variables which may be associated with cognitive performance. Negative symptoms have

routinely been associated with cognitive performance[58,59], including performance on the BACS[60]. Medication effects such as higher antipsychotic doses[61,62] and high anticholinergic antipsychotics[46,47,48] , have also been associated with deficits in cognitive performance. Future research should measure and adjust for these variables in order to determine the true association between cognition and treatment response without potential confounders.

It is also possible that premorbid histories of the sample may have resulted in a less consistent picture of cognitive performance between groups. For example, prior cannabis use, particularly during adolescence, has been found to improve cognitive performance on the BACS in comparison to those who haven't ever used cannabis[63]. In this investigation comparing performance on the BACS between patients with a schizophrenia diagnosis with and without adolescent cannabis use (ACU), those with ACU reported significantly higher composite scores, as well significant improvement in working memory and verbal memory tasks[63]. In our sample, 68% (N = 30) had previous experience of using cannabis, with the majority of this use occurring between ages 12-19 (N = 23). Therefore, it is possible that premorbid histories may have also blurred the cognitive differences between groups.

Conclusions

In this prospective cohort study, patients with a first episode diagnosis were assessed three times over a period of 6 weeks. Trajectory analyses using percentage change in PANSS total symptom scores identified two groups reflecting a good and poor response to antipsychotic medication. Baseline cognitive performance of these two groups did not predict response status at 6-weeks. This lack of discrimination between groups is potentially attributable to underpowered analyses as a result of small sample sizes but may also evidence that an association between cognition and treatment response is not observable in the first episode of schizophrenia. Overall this suggests that brief cognitive batteries for schizophrenia may not be a useful predictor of antipsychotic response in the first two-years of illness onset.

For peer review only

Contributorship:

J.H.M., A.E., J.F.W.D., R.J.D., O.D.H., L.K., C.C., A.S., R.O., S.Le., J.L., S.La., S.K., and E.Mik contributed to the design and implementation of the study. E.O., E.M., R.P., N.R., K.G., C.C., S.E.S., and K.V.S. aided in data collection. E.M. completed analyses and wrote the manuscript with the assistance of J.H.M. K.G., N.R., J.H.M., A.E., E.K., R.J.D., A.S., C.C., B.D., provided comments on the manuscript.

Funding statement:

STRATA is funded by a grant from the Medical Research Council (MRC) to JHM, grant reference MR/L011794. EM's PhD is funded by the MRC-doctoral training partnership studentship in Biomedical Sciences at King's College London. J.H.M., E.K., A.E., O.D.H. are part funded by the National Institute for Health Research (NIHR) Biomedical Research Centre at South London and Maudsley NHS Foundation Trust and King's College London. The views expressed are those of the authors and not necessarily those of the NHS, the MRC, the NIHR or the Department of Health.

Competing of Interests:

The remaining authors report no conflicts of interest.

Ethics approval:

This study was approved by the Health and Social Care Research Ethics Committee A; REC: 17/NI/0209.

Data sharing:

At the time of submission, the data governance frameworks are being put in place to make a fully anonymized version of the data available to the wider research community via the TranSMART data sharing platform: https://transmartfoundation.org/. To apply for access to the data, please contact J.H.M. at james.maccabe@kcl.ac.uk.

For peer review only

References

[1] McGlashan TH, Johannessen, JO. Early detection and intervention with schizophrenia: rationale. *Schizophrenia bulletin* 1996;22(2),201-222.

[2] Birchwood M, McGorry P, Jackson H. Early intervention in schizophrenia. *The British Journal of Psychiatry* 1997;170(1),2-5.

[3] Bond GR, Drake RE, Luciano A. Employment and educational outcomes in early intervention programmes for early psychosis: a systematic review. *Epidemiology and psychiatric sciences* 2015; 24(5),446-457.

[4] Bozzatello P, Bellino S, Rocca P. Predictive factors of treatment resistance in first episode of psychosis: A systematic review. *Frontiers in psychiatry* 2019;10, 67.

[5] Carbon, M, Correll CU. Clinical predictors of therapeutic response to antipsychotics in schizophrenia. *Dialogues in clinical neuroscience* 2014;16(4), 505.

[6] Samara, MT, Leucht C, Leeflang M, et al. Early improvement as a predictor of later response to antipsychotics in schizophrenia: a diagnostic test review. *American journal of psychiatry* 2015;172(7),617-629.

[7] Griffiths K, Millgate E, Egerton A, et al. Demographic and clinical variables associated with response to clozapine in schizophrenia: a systematic review and meta-analysis. *Psychological Medicine* 2021;1-11.

[8] Yoshimura B, Yada Y, So R, et al. The critical treatment window of clozapine in treatment-resistant schizophrenia: secondary analysis of an observational study. *Psychiatry research* 2017;250,65-70.

[9] Kennedy JL, Altar CA, Taylor DL, et al. The social and economic burden of treatment-resistant schizophrenia: a systematic literature review. *International clinical psychopharmacology* 2014;1;29(2):63-76.

[10] McCrone P, Dhanasiri S, Patel A, et al. The cost of mental health care in England to 2026. *The King's Fund* 2008;1-65.

[11] National Institute for Health and Care Excellence. Psychosis and schizophrenia in adults: prevention and management. *NICE*; 2014.

[12] Tsiachristas A, Thomas T, Leal J, et al. Economic impact of early intervention in psychosis services: results from a longitudinal retrospective controlled study in England. *BMJ open* 2016;1;6(10):e012611.

For peer review only - http://bmjopen.bmj.com/site/about/guidelines.xhtml

[13] Reichenberg A, Caspi A, Harrington H, et al. Static and dynamic cognitive deficits in childhood preceding adult schizophrenia: a 30-year study. *American Journal of Psychiatry* 2010;167(2),160-169.

[14] MacCabe JH, Wicks S, Löfving S, et al. Decline in cognitive performance between ages 13 and 18 years and the risk for psychosis in adulthood: a Swedish longitudinal cohort study in males. *Jama Psychiatry* 2013;70(3),261-270.

[15] Green MF, Kern RS, Heaton RK. Longitudinal studies of cognition and functional outcome in schizophrenia: implications for MATRICS. *Schizophrenia research* 2004;72(1),41-51.

[16] Carrión RE, Goldberg TE, McLaughlin D, et al. Impact of neurocognition on social and role functioning in individuals at clinical high risk for psychosis. *American Journal of Psychiatry* 2011;168(8),806-813.

[17] Horan WP, Green MF, DeGroot M, et al. Social cognition in schizophrenia, part 2: 12-month stability and prediction of functional outcome in first-episode patients. *Schizophrenia bulletin* 2012;38(4),865-872.

[18] Millgate E, Hide O, Lawrie SM, et al. Neuropsychological differences between treatment-resistant and treatment-responsive schizophrenia: a meta-analysis. *Psychological medicine* 2022;52(1),1-13.

[19] American Psychological Association. Distinguishing between screening and assessment for mental and behavioral health problems. 2014.

[20] Roebuck-Spencer TM, Glen T, Puente AE, et al. Cognitive screening tests versus comprehensive neuropsychological test batteries: a national academy of neuropsychology education paper. *Archives of Clinical Neuropsychology* 2017 Jun 1;32(4):491-8.

[21] Hurford IM, Marder SR, Keefe RS, et al. A brief cognitive assessment tool for schizophrenia: construction of a tool for clinicians. *Schizophrenia Bulletin* 2011 May 1;37(3):538-45.

[22] Andreasen NC, Carpenter JrWT, Kane JM, et al. Remission in schizophrenia: proposed criteria and rationale for consensus. *American Journal of Psychiatry* 2005;162(3),441-449.

[23] Kay SR, Fiszbein A, Opler LA. The positive and negative syndrome scale (PANSS) for schizophrenia. *Schizophrenia bulletin* 1987;13(2),261-276.

[24] Kemp R, Hayward P, Applewhaite G, et al. Compliance therapy in psychotic patients: randomised controlled trial. *Bmj* 1996;312(7027),345-349.

[25] Kemp R, Kirov G, Everitt B, et al. Randomised controlled trial of compliance therapy: 18-month follow-up. *The British Journal of Psychiatry* 1998;172(5),413-419.

For peer review only

[26] Jones BL, Nagin DS. A note on a Stata plugin for estimating group-based trajectory models. *Sociological Methods & Research* 2013;42(4),608-613.

[27] Leucht S, Kissling W, Davis JM. The PANSS should be rescaled. *Schizophrenia bulletin* 2010;36(3),461-462.

[28] Leucht S, Kane JM, Kissling W, et al. What does the PANSS mean?. *Schizophrenia research* 2005;79(2-3),231-238.

[29] Leucht S, Davis JM, Engel RR, et al. Definitions of response and remission in schizophrenia: recommendations for their use and their presentation. *Acta Psychiatrica Scandinavica* 2009;119,7-14.

[30] Sheehan DV, Lecrubier Y, Sheehan KH, et al. The Mini-International Neuropsychiatric Interview (MINI): the development and validation of a structured diagnostic psychiatric interview for DSM-IV and ICD-10. *The Journal of clinical psychiatry* 1998.

[31] Guy W. ECDEU assessment manual for psychopharmacology. *US Department of Health, Education, and Welfare, Public Health Service, Alcohol, Drug Abuse, and Mental Health Administration, National Institute of Mental Health, Psychopharmacology Research Branch, Division of Extramural Research Programs* 1976.

[32] Keefe RS, Goldberg TE, Harvey PD, et al. The Brief Assessment of Cognition in Schizophrenia: reliability, sensitivity, and comparison with a standard neurocognitive battery. *Schizophrenia research* 2004;68(2-3),283-297.

[33] Keefe RS, Harvey PD, Goldberg TE, et al. Norms and standardization of the Brief Assessment of Cognition in Schizophrenia (BACS). *Schizophrenia research* 2008;102(1-3),108-115.

[34] StataCorp. Stata Statistical Software: Release 15. 2017. College Station, TX: StataCorp LLC.

[35] StataCorp, LP. Stata multilevel mixed-effects reference manual. 2013. College Station, TX: StataCorp LP, 9(10).

[36] Kapur S, Zipursky R, Jones C, et al. Relationship between dopamine D2 occupancy, clinical response, and side effects: a double-blind PET study of first-episode schizophrenia. *American Journal of Psychiatry* 2000;157(4),514-520.

[37] Reilly JL, Harris MS, Keshavan MS, et al. Adverse effects of risperidone on spatial working memory in first-episode schizophrenia. *Archives of general psychiatry* 2006;63(11),1189-1197.

[38] Hill SK, Bishop JR, Palumbo D, et al. Effect of second-generation antipsychotics on cognition: current issues and future challenges. *Expert review of neurotherapeutics* 2010;10(1),43-57.

For peer review only

[39] Kane J, Honigfeld G, Singer J, et al. Clozapine for the treatment-resistant schizophrenic: a double-blind comparison with chlorpromazine. *Archives of general psychiatry* 1988;45(9),789-796.

[40] Guilera G, Pino O, Gómez-Benito J, et al. Antipsychotic effects on cognition in schizophrenia: a meta-analysis of randomised controlled trials. *The European Journal of Psychiatry* 2009;23(2),77-89.

[41] Goldberg TE, Goldman RS, Burdick KE, et al. Cognitive improvement after treatment with second-generation antipsychotic medications in first-episode schizophrenia: is it a practice effect?. *Archives of general psychiatry* 2007;64(10),1115-1122.

[42] Lees J, Applegate E, Emsley R, et al. Calibration and cross-validation of MCCB and CogState in schizophrenia. *Psychopharmacology* 2015;232(21),3873-3882.

[43] Nuechterlein KH, Green MF, Kern RS, et al. The MATRICS Consensus Cognitive Battery, part 1: test selection, reliability, and validity. *American Journal of Psychiatry* 2008;165(2),203-213.

[44] Maruff P, Thomas E, Cysique L, et al. Validity of the CogState brief battery: relationship to standardized tests and sensitivity to cognitive impairment in mild traumatic brain injury, schizophrenia, and AIDS dementia complex. *Archives of Clinical Neuropsychology* 2009;24(2),165-178.

[45] Lieberman JA. Managing anticholinergic side effects. *Primary care companion to the Journal of clinical psychiatry* 2004;6(suppl2),20.

[46] Strauss ME, Reynolds KS, Jayaram G, et al. Effects of anticholinergic medication on memory in schizophrenia. *Schizophrenia Research* 1990;3(2),127-129.

[47] Campbell N, Boustani M, Limbil T, et al. The cognitive impact of anticholinergics: a clinical review. *Clinical interventions in aging* 2009;4,225.

[48] Joshi YB, Thomas ML, Braff DL, et al. Anticholinergic Medication Burden–Associated Cognitive Impairment in Schizophrenia. *American Journal of Psychiatry* 2021;appi-ajp.

[49] Stroup TS, Gray N. Management of common adverse effects of antipsychotic medications. *World Psychiatry* 2018;17(3),341-356.

[50] Abdin E, Chong SA, Vaingankar JA, et al. Trajectories of positive, negative and general psychopathology symptoms in first episode psychosis and their relationship with functioning over a 2-year follow-up period. *PloS one* 2017;12(11),e0187141.

[51] Levine SZ, Rabinowitz J. Trajectories and antecedents of treatment response over time in early-episode psychosis. *Schizophrenia bulletin* 2010;36(3),624-632.

For peer review only

[52] Obermeier M, Mayr A, Schennach-Wolff R, et al. Should the PANSS be rescaled?. *Schizophrenia bulletin* 2010;36(3),455-460.

[53] Trampush JW, Lencz T, DeRosse P, et al. Relationship of cognition to clinical response in first-episode schizophrenia spectrum disorders. *Schizophrenia bulletin* 2015;41(6),1237-1247.

[54] Bilder RM, Goldman RS, Robinson D, et al. Neuropsychology of first-episode schizophrenia: initial characterization and clinical correlates. *American Journal of Psychiatry* 2000;157(4),549-559.

[55] Zanelli J, Mollon J, Sandin S, et al. Cognitive change in schizophrenia and other psychoses in the decade following the first episode. *American Journal of Psychiatry* 2019 Oct 1;176(10):811-9.

[56] Emsley R, Rabinowitz J, Medori R. Time course for antipsychotic treatment response in first-episode schizophrenia. *American Journal of Psychiatry* 2006 Apr;163(4):743-5.

[57] Howes OD, McCutcheon R, Agid O, et al. Treatment-resistant schizophrenia: treatment response and resistance in psychosis (TRRIP) working group consensus guidelines on diagnosis and terminology. *American Journal of Psychiatry* 2017 Mar 1;174(3):216-29.

[58] Addington J, Addington D, Maticka-Tyndale E. Cognitive functioning and positive and negative symptoms in schizophrenia. *Schizophrenia research* 1991 Sep 1;5(2):123-34.

[59] Hoffman RE, Rakfeldt J. Cognition, negative symptoms, and diagnosis: a comparison of schizophrenic, bipolar, and control samples. *Neurosciences* 1997;9(1):81-9.

[60] Tanaka T, Tomotake M, Ueoka Y, et al. Clinical correlates associated with cognitive dysfunction in people with schizophrenia. *Psychiatry and clinical neurosciences* 2012 Oct;66(6):491-8.

[61] Elie D, Poirier M, Chianetta JM, et al. Cognitive effects of antipsychotic dosage and polypharmacy: a study with the BACS in patients with schizophrenia and schizoaffective disorder. *Journal of Psychopharmacology* 2010 Jul;24(7):1037-44.

[62] Ballesteros A, Sánchez-Torres AM, López-Ilundain JM, et al. Is cognitive impairment associated with antipsychotic dose and anticholinergic equivalent loads in first-episode psychosis?. *Psychological medicine* 2018 Oct;48(13):2247-56.

[63] Hanna RC, Shalvoy A, Cullum CM, et al. Cognitive function in individuals with psychosis: moderation by adolescent cannabis use. Schizophrenia bulletin 2016;42(6),1496-1503.

For peer review only

Figure legends

Figure 1. A CONSORT based flowchart illustrating the number of participants and exclusions at each stage of the study trial.

Figure 2. Trajectory model of total PANSS score percentage change from baseline modelled over days since baseline assessment. The dotted linear trajectory reflects treatment non-responders and the complete line treatment responders. The grey dotted lines around each trajectory reflect the confidence intervals for trajectory each group. Percentages reflect the estimated amount of the sampled population included in each trajectory.

For peer review only

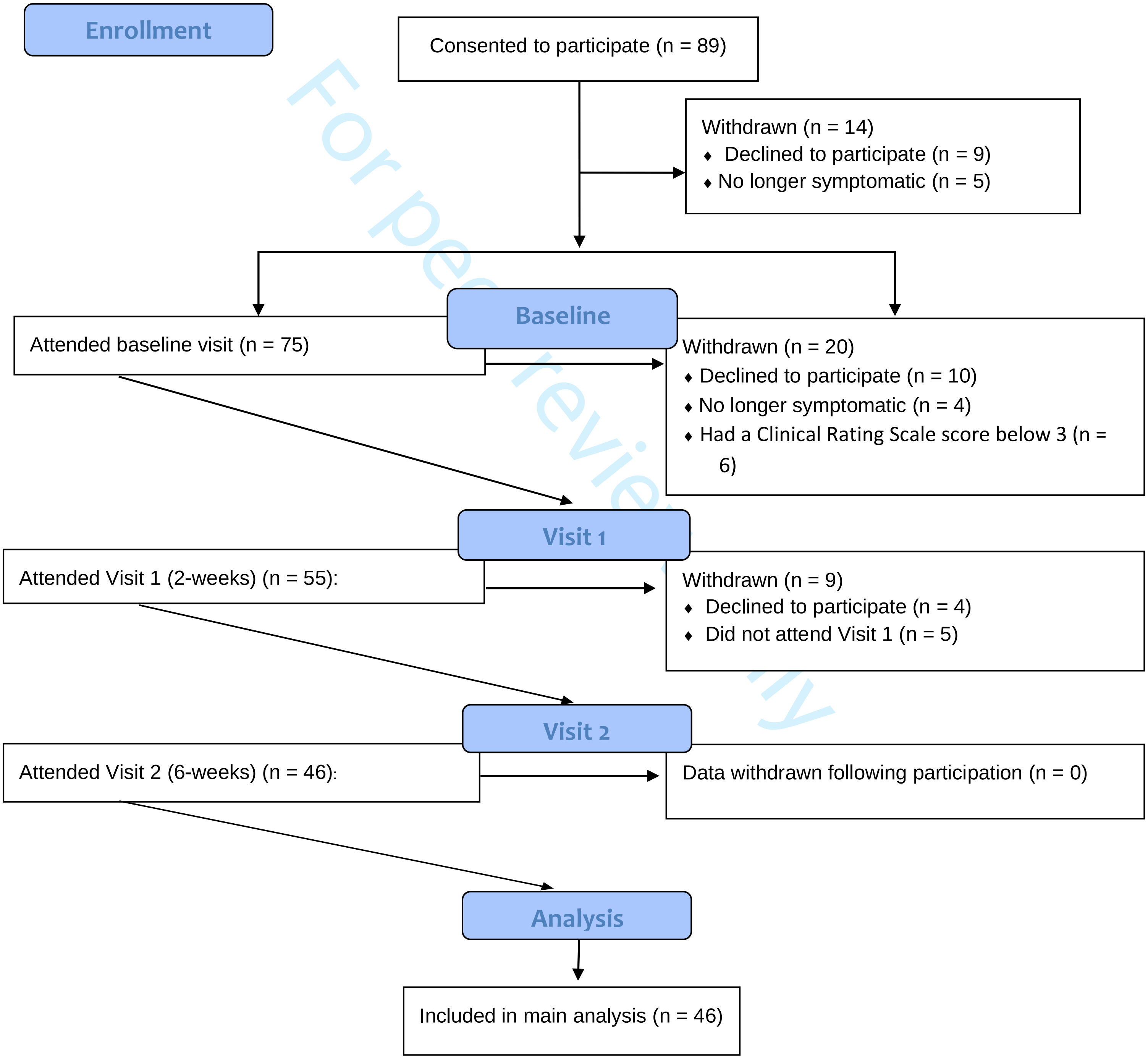

For peer review only

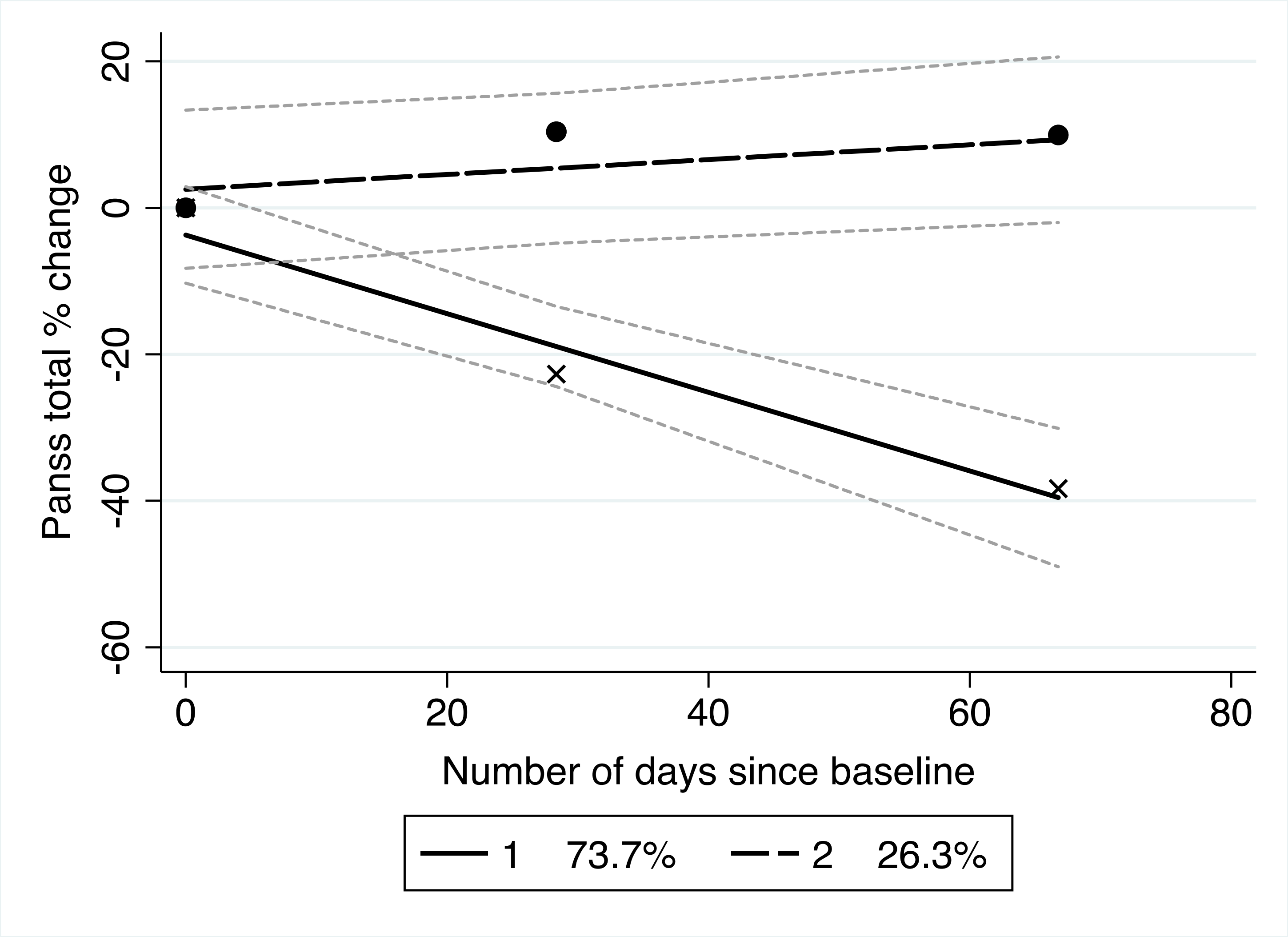
Panss total % change
20
0
-20
-40
-60
0
20
40
60
80
Number of days since baseline
1 73.7%
2 26.3%

Supplementary material

Table S.1

Results from univariable and multivariable logistic regression models for response status using PANSS total >20% reduction criteria and baseline BACS performance

	Unadjusted					Adjusted for age, gender and DUP				
BACS task	β	SE	95%CI	OR	P-value	β	SE	95%CI	OR	P-value
Verbal Memory	-0.01	0.03	-0.07 ; 0.06	0.99	.819	<-0.01	0.04	-0.08 ; 0.07	1.00	.920
Digit Sequencing	-0.10	0.10	-0.29 ; 0.09	0.90	.297	-0.06	0.11	-0.27 ; 0.16	0.95	.604
Verbal Fluency	0.04	0.05	-0.06 ; 0.14	1.04	.403	0.07	0.06	-0.05 ; 0.18	1.07	.259
Token Motor	0.03	0.04	-0.04 ; 0.10	1.03	.432	0.06	0.05	-0.03 ; 0.15	1.06	.218
Symbol Coding	-0.04	0.03	-0.11 ; 0.02	0.96	.206	-0.05	0.04	-0.12 ; 0.03	0.96	.237
Tower of London	0.07	0.09	-0.11 ; 0.24	1.07	.445	0.07	0.09	-0.12 ; 0.25	1.07	.465
t score composite	<0.01	0.03	-0.06 ; 0.07	1.00	.924	0.01	0.03	-0.06 ; 0.07	1.00	.892
z score composite	0.02	0.33	-0.63 ; 0.66	1.02	.960	0.03	0.34	-0.63 ; 0.69	1.03	.931

Note. *BACS* = Brief Assessment of Cognition in Schizophrenia; *DUP* = duration of untreated psychosis; *CIs* = confidence intervals.

For peer review only

Table S.2

Results from unadjusted and adjusted growth curve models comparing trajectory groups on BACS performance across all visits

	Unadjusted				Adjusted for age, gender and DUP			
BACS task	β	SE	95%CI	P-value	β	SE	95%CI	P-value
Verbal Memory	-2.15	45.79	-13. 49 ; 9.19	.710	-2.67	5.88	-14.20 ;8.86	.650
Digit Sequencing	-2.25	1.63	-5.45 ; 1 0.95	.167	-2.03	1.62	-5.19 ; 1.14	.210
Verbal Fluency	-0.90	3.29	-7.36 ; 5.55	.784	-1.31	2.99	-7.17 ; 4.54	.660
Token Motor	-2.00	6.39	-14.52 ; 10.53	.755	-0.45	5.89	11.99 ; 11.10	.939
Symbol Coding	-3.81	5.56	-14.71 ; 7.08	.493	-4.25	5.42	-14.87 ; 6.37	.433
Tower of London	0.18	1.56	-2.87 ; 3.24	.906	0.17	1.57	-2.90 ; 3.25	.912
t score composite	-2.49	5.70	-13.66 ; 8.68	.662	-2.43	5.78	-13.76 ; 8.89	.674
z score composite	-0.25	0.57	-1.37 ; 0.87	.658	-0.25	0.58	-1.38 ; 0.88	.665

Note. *BACS* = Brief Assessment of Cognition in Schizophrenia; *DUP* = duration of untreated psychosis; *CIs* = confidence intervals

For peer review only

Table S.3

Results from unadjusted and adjusted growth curve models comparing PANSS >20% reduction groups on BACS performance across all visits

	Unadjusted				Adjusted for age, gender and DUP			
BACS task	β	SE	95%CI	P-value	β	SE	95%CI	P-value
Verbal Memory	-4.18	4.21	-12.44 ; 4.08	.321	3.27	4.34	-11.78 ; 5.23	.451
Digit Sequencing	-2.36	1.35	-5.01 ; 0.29	.081	-1.78	1.34	-4.41 ; 0.86	.186
Verbal Fluency	-0.97	2.55	-5.97 ; 4.02	.703	-0.54	2.49	-5.42 ; 4.35	.830
Token Motor	1.81	5.52	-9.01 ; 12.62	.744	5.60	5.07	-4.33 ; 15.53	.269
Symbol Coding	-4.23	3.92	-11.92 ; 3.46	.281	-4.13	4.03	-12.03 ; 3.78	.306
Tower of London	-0.77	1.25	-3.22 ; 1.68	.538	-0.56	1.30	-3.10 ; 1.98	.667
t score composite	-2.39	4.75	-11.71 ; 6.93	.615	-1.56	4.84	-11.03 ; 7.92	.747
z score composite	-0.25	0.48	-1.18 ; 0.68	.600	-0.17	0.48	-1.12 ; 0.78	.728

Note. *BACS* = Brief Assessment of Cognition in Schizophrenia; *DUP* = duration of untreated psychosis; *CIs* = confidence intervals

For peer review only

Table S.4

Descriptive statistics of clinical and demographic variables for each trajectory group at baseline assessments

	Non-responder			Responder		
Variable	N	M	SD	N	M	SD
Age (at consent)	6	29.57	6.70	39	26.90	8.41
Gender (*male*)	6	-	-	27	-	-
Gender (*female*)	1	-	-	12	-	-
Age of illness onset (years)	7	27.54	8.09	39	26.34	8.60
Duration from 1st Psychotic symptom (days) to baseline antipsychotic (DUP)	7	177.09	207.93	39	261.08	251.38
Duration from 1st contact with mental health services (days) to baseline antipsychotic	7	461.89	801.88	39	325.88	567.82
Chlorpromazine equivalents (mg/day)	7	271.43	249.76	39	159.92	75.02
Number of hospitalisations	7	1.00	1.00	39	0.87	0.57
Years of education	6	17.00	2.53	36	13.06	2.47
PANSS positive	7	11.14	6.67	38	12.08	4.44
PANSS negative	7	12.57	5.77	38	9.95	7.04
PANSS general	7	19.57	8.70	38	19.37	8.66
PANSS total	7	43.29	13.52	38	41.39	16.68

Note. PANSS = Positive and Negative Symptom Scale.

For peer review only

Table S.5

Baseline cognitive performance for both groups using >20% PANSS reduction criteria

	Non-responder			Responder		
BACS measure	N	Mean	SD	N	Mean	SD
Verbal Memory	13	37.54	8.27	20	36.65	12.73
Digit Sequencing	12	19.25	4.20	19	17.68	3.97
Verbal Fluency	14	27.36	6.44	20	29.45	7.75
Token Motor	13	63.23	10.94	18	66.39	11.24
Symbol Coding	12	44.83	9.47	20	39.35	12.75
Tower of London	12	14.17	5.11	17	15.41	3.79
tscore composite	10	26.30	13.35	17	26.77	12.28
zscore composite	10	-2.36	1.31	17	-2.34	1.22

Note. *BACS* = Brief Assessment of Cognition in Schizophrenia; *PANSS* = Positive and Negative Symptom Scale.

For peer review only

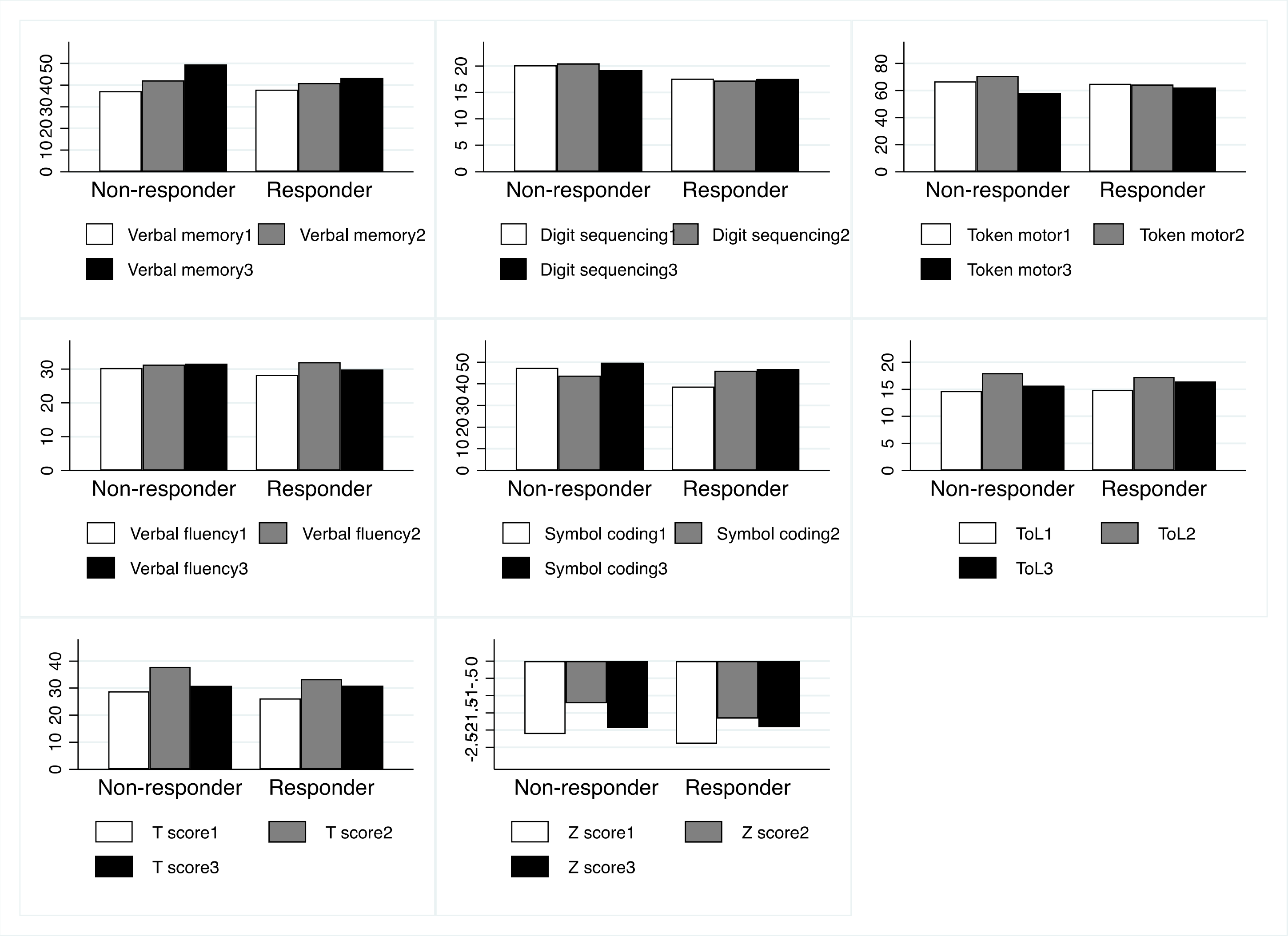

Figure S.1

Bar graphs comparing mean performance on BACS measures between trajectory groups (non-responder vs. responder) at each visit (*white* = baseline, *grey* = 2-week, *black* = 6-week)

Note. *BACS* = Brief Assessment of Cognition in Schizophrenia; *ToL* = Tower of London.

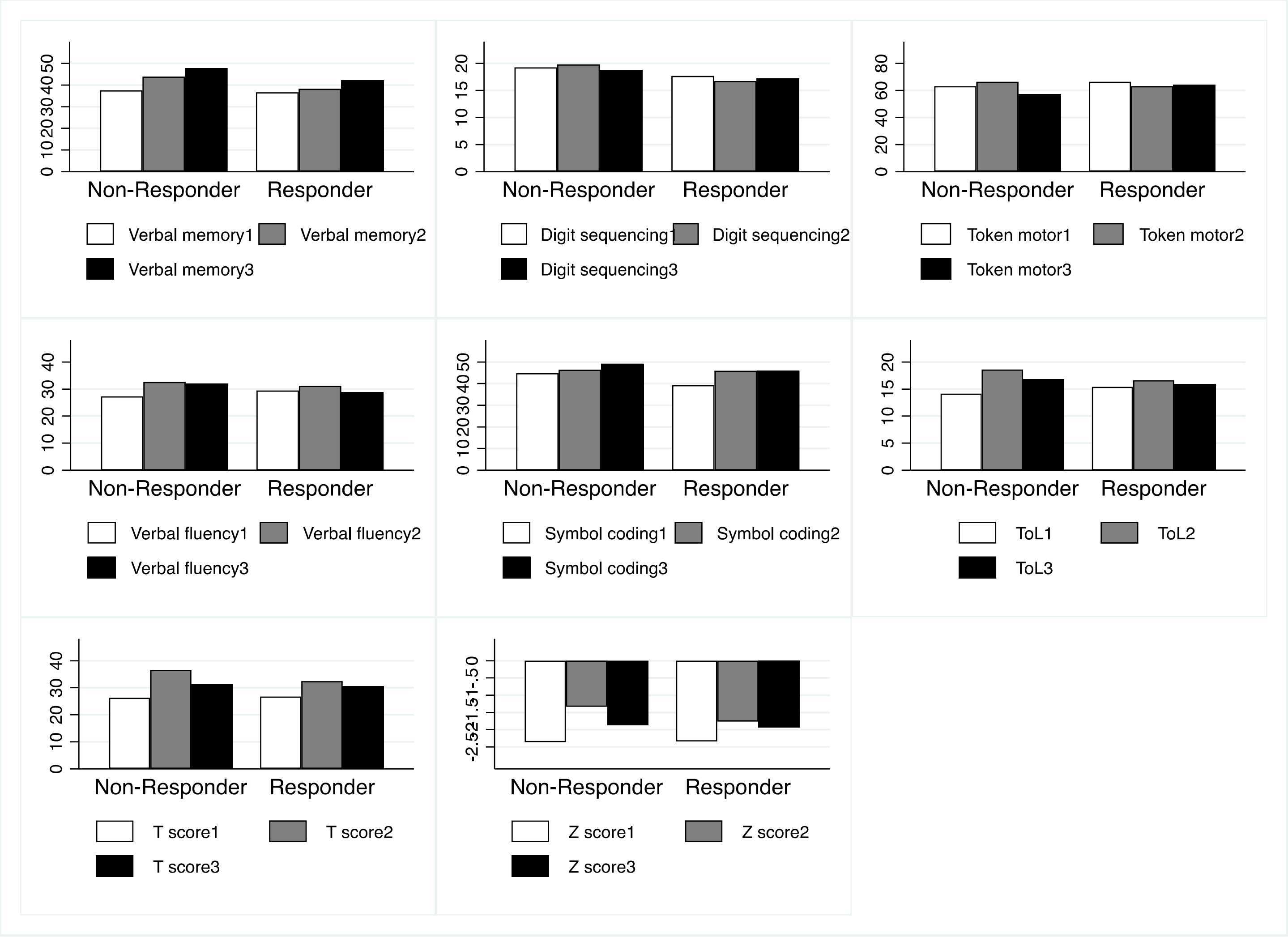

Figure S.2

Bar graphs comparing mean performance on BACS measures using >20% PANSS reduction criteria (non-responder vs. responder) at each visit (*white* = baseline, *grey* = 2-week, *black* = 6-week)

Note. *BACS* = Brief Assessment of Cognition in Schizophrenia; *ToL* = Tower of London.

For peer review only

STROBE Statement—checklist of items that should be included in reports of observational studies

	Item No	Recommendation	Noted on pg.
**Title and abstract**	1	(*a*) Indicate the study's design with a commonly used term in the title or the abstract	1,2,3
		(*b*) Provide in the abstract an informative and balanced summary of what was done and what was found	1,2,3
**Introduction**			
Background/rationale	2	Explain the scientific background and rationale for the investigation being reported	5,6
Objectives	3	State specific objectives, including any prespecified hypotheses	6
**Methods**			
Study design	4	Present key elements of study design early in the paper	7
Setting	5	Describe the setting, locations, and relevant dates, including periods of recruitment, exposure, follow-up, and data collection	7
Participants	6	(*a*) *Cohort study*—Give the eligibility criteria, and the sources and methods of selection of participants. Describe methods of follow-up<br>*Case-control study*—Give the eligibility criteria, and the sources and methods of case ascertainment and control selection. Give the rationale for the choice of cases and controls<br>*Cross-sectional study*—Give the eligibility criteria, and the sources and methods of selection of participants	7,8
		(*b*) *Cohort study*—For matched studies, give matching criteria and number of exposed and unexposed<br>*Case-control study*—For matched studies, give matching criteria and the number of controls per case	N/A
Variables	7	Clearly define all outcomes, exposures, predictors, potential confounders, and effect modifiers. Give diagnostic criteria, if applicable	7,8
Data sources/ measurement	8*	For each variable of interest, give sources of data and details of methods of assessment (measurement). Describe comparability of assessment methods if there is more than one group	7,8
Bias	9	Describe any efforts to address potential sources of bias	9
Study size	10	Explain how the study size was arrived at	N/A
Quantitative variables	11	Explain how quantitative variables were handled in the analyses. If applicable, describe which groupings were chosen and why	8,9
Statistical methods	12	(*a*) Describe all statistical methods, including those used to control for confounding	8,9
		(*b*) Describe any methods used to examine subgroups and interactions	8,9
		(*c*) Explain how missing data were addressed	N/A
		(*d*) *Cohort study*—If applicable, explain how loss to follow-up was addressed<br>*Case-control study*—If applicable, explain how matching of cases and controls was addressed<br>*Cross-sectional study*—If applicable, describe analytical methods taking account of sampling strategy	N/A
		(*e*) Describe any sensitivity analyses	8,9

For peer review only - http://bmjopen.bmj.com/site/about/guidelines.xhtml

Continued on next page

For peer review only

For peer review only - http://bmjopen.bmj.com/site/about/guidelines.xhtml

**Results**			
Participants	13*	(a) Report numbers of individuals at each stage of study—eg numbers potentially eligible, examined for eligibility, confirmed eligible, included in the study, completing follow-up, and analysed	6,7
		(b) Give reasons for non-participation at each stage	7,8
		(c) Consider use of a flow diagram	N/A
Descriptive data	14*	(a) Give characteristics of study participants (eg demographic, clinical, social) and information on exposures and potential confounders	9,10
		(b) Indicate number of participants with missing data for each variable of interest	N/A
		(c) *Cohort study*—Summarise follow-up time (eg, average and total amount)	N/A
Outcome data	15*	*Cohort study*—Report numbers of outcome events or summary measures over time	10,11,supplem.
		Case-control study—Report numbers in each exposure category, or summary measures of exposure	N/A
		Cross-sectional study—Report numbers of outcome events or summary measures	N/A
Main results	16	(*a*) Give unadjusted estimates and, if applicable, confounder-adjusted estimates and their precision (eg, 95% confidence interval). Make clear which confounders were adjusted for and why they were included	17, supplem.
		(*b*) Report category boundaries when continuous variables were categorized	N/A
		(*c*) If relevant, consider translating estimates of relative risk into absolute risk for a meaningful time period	17, supplem.
Other analyses	17	Report other analyses done—eg analyses of subgroups and interactions, and sensitivity analyses	13-15, Supplem.
**Discussion**			
Key results	18	Summarise key results with reference to study objectives	20,21
Limitations	19	Discuss limitations of the study, taking into account sources of potential bias or imprecision. Discuss both direction and magnitude of any potential bias	23,24
Interpretation	20	Give a cautious overall interpretation of results considering objectives, limitations, multiplicity of analyses, results from similar studies, and other relevant evidence	20,21,23
Generalisability	21	Discuss the generalisability (external validity) of the study results	20,21,23
**Other information**			
Funding	22	Give the source of funding and the role of the funders for the present study and, if applicable, for the original study on which the present article is based	25

*Give information separately for cases and controls in case-control studies and, if applicable, for exposed and unexposed groups in cohort and cross-sectional studies.

Note: An Explanation and Elaboration article discusses each checklist item and gives methodological background and published examples of transparent reporting. The STROBE checklist is best used in conjunction with this article (freely available on the Web sites of PLoS Medicine at http://www.plosmedicine.org/, Annals of Internal Medicine at http://www.annals.org/, and Epidemiology at http://www.epidem.com/). Information on the STROBE Initiative is available at www.strobe-statement.org.

For peer review only

BMJ Open

Cognitive function and treatment response trajectories in first episode schizophrenia: evidence from a prospective cohort study.

Journal:	*BMJ Open*
Manuscript ID	bmjopen-2022-062570.R2
Article Type:	Original research
Date Submitted by the Author:	29-Sep-2022
Complete List of Authors:	Millgate, Edward; King's College London Institute of Psychiatry Psychology and Neuroscience, Department of Psychosis Studies<br>Griffiths, Kira; King's College London Institute of Psychiatry Psychology and Neuroscience, Department of Psychosis Studies<br>Egerton, Alice; King's College London Institute of Psychiatry Psychology and Neuroscience, Department of Psychosis Studies; South London and Maudsley NHS Foundation Trust, NIHR Biomedical Research Centre<br>Kravariti, Eugenia; King's College London Institute of Psychiatry Psychology and Neuroscience, Department of Psychosis Studies; South London and Maudsley NHS Foundation Trust, NIHR Biomedical Research Centre<br>Casetta, Cecilia; King's College London Institute of Psychiatry Psychology and Neuroscience, Department of Psychosis Studies; South London and Maudsley Mental Health NHS Trust, National Psychosis Service<br>Deakin, Bill; University of Manchester, Faculty of Medicine, Biology and Health<br>Drake, Richard; University of Manchester, Division of Psychology & Mental Health; Greater Manchester Mental Health NHS Foundation Trust<br>Howes, Oliver; King's College London Institute of Psychiatry Psychology and Neuroscience, Department of Psychosis Studies; South London and Maudsley NHS Foundation Trust, NIHR Biomedical Research Centre<br>Kassoumeri, Laura; King's College London Institute of Psychiatry Psychology and Neuroscience, Department of Psychosis Studies<br>Khan , Sobia; University of Manchester, Faculty of Medicine, Biology and Health<br>Lankshear, Steve; Greater Manchester Mental Health NHS Foundation Trust<br>Lees, Jane; Greater Manchester Mental Health NHS Foundation Trust; University of Manchester, Faculty of Medicine, Biology and Health<br>Lewis, Shon; University of Manchester; Greater Manchester Mental Health NHS Foundation Trust<br>Mikulskaya, Elena ; University of Manchester, Faculty of Medicine, Biology and Health<br>Oloyede, Ebenezer; King's College London Institute of Psychiatry Psychology and Neuroscience, Department of Psychosis Studies; South London and Maudsley NHS Foundation Trust, Pharmacy Department<br>Owens, Rebecca; University of Manchester, Faculty of Medicine, Biology and Health<br>Pollard, Rebecca; King's College London Institute of Psychiatry

	Psychology and Neuroscience, Department of Psychosis Studies<br>Rich, Nathalie; University College London, Department of Epidemiology & Applied Clinical Research<br>Smart, Sophie; Cardiff University, Division of Psychological Medicine and Clinical Neurosciences<br>Segev , Aviv; King's College London Institute of Psychiatry Psychology and Neuroscience, Department of Psychosis Studies; Shalvata Mental Health Center, Sackler Faculty of Medicine<br>Verena Sendt , Kyra; King's College London Institute of Psychiatry Psychology and Neuroscience, Department of Psychosis Studies<br>MacCabe, James; Institute of Psychiatry Psychology and Neuroscience Division of Academic Psychiatry, Department of Psychosis Studies; South London and Maudsley NHS Foundation Trust, National Psychosis Service

**Primary Subject Heading**:	Mental health
Secondary Subject Heading:	Public health, Patient-centred medicine, Mental health
Keywords:	Schizophrenia & psychotic disorders < PSYCHIATRY, Adult psychiatry < PSYCHIATRY, MENTAL HEALTH

I, the Submitting Author has the right to grant and does grant on behalf of all authors of the Work (as defined in the below author licence), an exclusive licence and/or a non-exclusive licence for contributions from authors who are: i) UK Crown employees; ii) where BMJ has agreed a CC-BY licence shall apply, and/or iii) in accordance with the terms applicable for US Federal Government officers or employees acting as part of their official duties; on a worldwide, perpetual, irrevocable, royalty-free basis to BMJ Publishing Group Ltd ("BMJ") its licensees and where the relevant Journal is co-owned by BMJ to the co-owners of the Journal, to publish the Work in this journal and any other BMJ products and to exploit all rights, as set out in our licence.

The Submitting Author accepts and understands that any supply made under these terms is made by BMJ to the Submitting Author unless you are acting as an employee on behalf of your employer or a postgraduate student of an affiliated institution which is paying any applicable article publishing charge ("APC") for Open Access articles. Where the Submitting Author wishes to make the Work available on an Open Access basis (and intends to pay the relevant APC), the terms of reuse of such Open Access shall be governed by a Creative Commons licence – details of these licences and which Creative Commons licence will apply to this Work are set out in our licence referred to above.

Other than as permitted in any relevant BMJ Author's Self Archiving Policies, I confirm this Work has not been accepted for publication elsewhere, is not being considered for publication elsewhere and does not duplicate material already published. I confirm all authors consent to publication of this Work and authorise the granting of this licence.

For peer review only

Journal: BMJ Open

Abstract word count: 310 (288 without headings)

Word count: 4341

Original submission date: 4th March 2022

Submission date following corrections: 18th July 2022

Submission date following final corrections: 28th September 2022

Cognitive function and treatment response trajectories in first episode schizophrenia: evidence from a prospective cohort study.

Edward Millgate[1], Kira Griffiths[1], Alice Egerton[1,2], Eugenia Kravariti[1,2], Cecilia Casetta[1,3], Bill Deakin[4,5], Richard J Drake[4,5,6], Oliver D Howes[1,2], Laura Kassoumeri[1], Sobia Khan[4], Steve Lankshear[5], Jane Lees[4,5,6], Shôn Lewis[4,5,6], Elena Mikulskaya[4], Ebenezer Oloyede[1,7], Rebecca Owens[4], Rebecca Pollard[1], Nathalie Rich[8], Sophie E Smart[9], Aviv Segev[1,10,11], Kyra-Verena Sendt[1], The STRATA Consortium, James H MacCabe[1,3]*

[1] Department of Psychosis Studies, Institute of Psychiatry, Psychology and Neuroscience, King's College London, UK

[2] NIHR Biomedical Research Centre at South London and Maudsley NHS Foundation Trust, London, UK

[3] National Psychosis Service, South London and Maudsley NHS Foundation Trust, London, UK

[4] Division of Psychology and Mental Health, School of Biological Sciences, Faculty of Biology, Medicine and Health, University of Manchester, Manchester, UK

[5] Greater Manchester Mental Health NHS Foundation Trust, Manchester, UK

[6] Manchester Academic Health Science Centre, Manchester, UK

[7] Pharmacy Department, South London and Maudsley NHS Foundation Trust, UK

[8] Department of Epidemiology & Applied Clinical Research, University College London, UK

[9] MRC Centre for Neuropsychiatric Genetics and Genomics, Division of Psychological Medicine and Clinical Neurosciences, Cardiff University, Cardiff, UK

[10] Shalvata Mental Health Centre, Israel

[11] Sackler Faculty of Medicine, Tel Aviv University, Israel

For peer review only

*Corresponding author: Edward.millgate@kcl.ac.uk

Department of Psychosis Studies,

Institute of Psychiatry, Psychology & Neuroscience,

16 de Crespigny Park,

Denmark Hill, London,

United Kingdom, SE5 8AF

For peer review only

Abstract

Objectives: This prospective cohort study tested for associations between baseline cognitive performance in individuals early within their first episode and antipsychotic treatment of psychosis. We hypothesised that poorer cognitive functioning at the initial assessment would be associated with poorer antipsychotic response following the subsequent six weeks.

Setting: NHS service users with a first episode schizophrenia diagnosis, recently starting antipsychotic medication, recruited from two UK sites (King's College London, UK & University of Manchester, UK). Participants attended three study visits following screening.

Participants: Eighty-nine participants were recruited, with 46 included in the main analysis. Participants required to be within the first two years of illness onset, had received minimal antipsychotic treatment, have the capacity to provide consent, and be able to read and write in English. Participants were excluded if they met remission criteria or showed mild to no symptoms.

Primary and secondary outcomes: Antipsychotic response was determined at 6-weeks using the Positive and Negative Syndrome Scale (PANSS), with cognitive performance assessed at each visit using the Brief Assessment of Cognition in Schizophrenia (BACS). The groups identified (responders and non-responders) from trajectory analyses, as well as from >20% PANSS criteria, were compared on baseline BACS performance.

Results: Trajectory analyses identified 84.78% of the sample as treatment responsive, and the remaining 15.22% as treatment non-responsive. Unadjusted and adjusted logistic regressions observed no significant relationship between baseline BACS on subscale and total performance (BACS t-score: OR = 0.98, $p = .620$, Cohen's d = .218) and antipsychotic response at 6-weeks.

Conclusions: This investigation identified two clear trajectories of treatment response in the first six weeks of antipsychotic treatment. Responder and non-responder groups did not significantly differ on performance on the BACS, suggesting that larger samples may be required or that an association between cognitive performance and antipsychotic response is not observable in the first two-years of illness onset.

Trial registration number: REC: 17/NI/0209

Keywords: cognition, antipsychotic response, BACS, trajectories, first episode schizophrenia

For peer review only

Article summary

Strengths and limitations of this study

- The study examined cognitive performance in a multicentre sample of first episode psychosis
- Cognitive performance was assessed at each study period with the BACS, a reliable and well-validated brief test battery which is specifically designed for schizophrenia
- The study used symptom ratings on the PANSS to determine response to treatment, a gold standard proxy within the current research field.

For peer review only

Introduction

Prompt intervention with pharmacological therapy for individuals with schizophrenia has been extensively recommended in the literature[1,2] and is reported to be associated with better functional outcomes[3,4,5]. As observed by Carbon & Correll[5], a lack of early response and improvement to antipsychotic medication is a strong predictor of later non-response. A recent diagnostic test review has even argued that non/minimal response to antipsychotic medication in the first 2 weeks of treatment may be a sufficient indication to switch antipsychotic[6]. Early and accurate detection of treatment non-responders at first episode is also more likely to result in timely treatment with clozapine, which may be associated with better outcomes[7]. Indeed, Yoshimura et al[8] found that response to clozapine was ~80% in treatment resistant patients who were commenced on clozapine early in their illness course, with this depreciating to ~30% when clozapine initiation was delayed by more than 2.8years[7,8].

Individuals who do not respond to antipsychotic medication are reported to have higher rates of smoking (56%), substance and alcohol abuse (51%) and suicidal ideation (44%), with annual treatment costs being 3 to 11 times larger than those who respond to antipsychotic medication[9]. In 2007, it was estimated that schizophrenia accounted for 30% of the total expenditure for adult mental health and social care services[10], with additional economic and societal costs due to unemployment or absence from work. These total service costs, which were estimated at £2.2 billion in 2007, have the potential to reach £3.7 billion by 2026[11]. However, it has been suggested that early intervention programmes could aid in reducing these costs substantially if adequately introduced in first episode psychosis[12], as earlier onset schizophrenia is associated with greater expected costs[11].

Early cognitive deficits may be predictive of subsequent antipsychotic response in the first episode of illness and could aid in delivering fast, early, intervention. Cognitive

For peer review only

dysfunction in schizophrenia is observable prior to illness onset[13,14] and is strongly associated with poorer functional outcomes[15,16,17]. A recent meta-analysis comparing cognitive performance in known cases of antipsychotic treatment resistance and response[18] observed worse performance in treatment resistant samples across cognitive domains, with the strongest effect in measures of verbal memory and learning and language functions. However, it is possible that illness chronicity and exposure to long-term antipsychotic treatment may have influenced these findings.

Based on the current existing literature it is plausible to argue that there may be quantifiable cognitive differences between individuals who respond to antipsychotic medication and those who do not in the early stages of the illness; seeing as deficits in cognition are observable prior to illness onset[14,15] and poor early non-response to medication being predictive of future non-response[5]. Therefore if differences are observed between groups of differing response to medication (i.e. responders and non-responders), early in their illness and treatment, this will broaden our understanding of the relationship between cognition, schizophrenia, and antipsychotic response, as well as aid clinical utility by using brief cognitive measures as a screening for potential non-response in the first episode of schizophrenia. The American Psychological Association's Working Group on Screening and Assessment have provided guidelines for determining the appropriateness of a neuropsychological measure for cognitive screening within a clinical setting[19]. The guidelines are as follows: i. provide identification for those at high risk for impairment, ii. sensitive enough to identify those who need further review, iii. brief and narrow in scope, iv. can be administered at routine visits, v. can be administered by support staff or clinicians electronically and vi. can be used to monitor progress and outcomes[20]. In high-income countries, the use of brief assessment batteries such as the BACS have been found to meet these criteria put forward by the APA working group[21].

For peer review only - http://bmjopen.bmj.com/site/about/guidelines.xhtml

Therefore, this prospective cohort study tested for associations between baseline cognitive performance using a brief cognitive battery, assessed at the initiation of antipsychotic treatment, in individuals early within their first episode of psychosis and their subsequent response to antipsychotic treatment. We hypothesised that poorer cognitive functioning at the initial assessment would be associated with poorer response over the subsequent six weeks of antipsychotic treatment.

Methods

Design

The study used a prospective cohort design with a sample of patients with first-episode schizophrenia. Participants were assessed over a period of 6-weeks, with two follow-up visits following baseline and screening assessments.

Setting

The study was part of the 'Schizophrenia: Treatment Resistance and Therapeutic Advances' (STRATA) consortium which included two UK sites in this study; King's College London (London, UK) and University of Manchester (Manchester, UK). The aim of the STRATA consortium is to identify neurobiological, cognitive, and genetic biomarkers of antipsychotic treatment resistance and non-response within schizophrenia and other related psychotic disorders.

Patient and Public Involvement

In the early development and design of the study consultations with the NIHR Maudsley Biomedical Research Centre (BRC) Service User Advisory Group (SUAG) took place to determine the feasibility of the study and its' assessments for service users. The NIHR Maudsley BRC Feasibility and Acceptability Support Team for Researchers (FAST-R) service was also used in order to receive feedback on consent forms, information sheets and protocols, as well as advice for recruitment strategies for service users.

Participants

For peer review only

89 participants aged between 18 – 65 years with a DSM-5 diagnosis of schizophrenia, schizoaffective, schizophreniform disorder, or psychosis (non-specified) (ICD-10: F20-F29) were recruited across two UK sites (King's College London and University of Manchester). Inclusion required that participants were within the first two years of illness onset, defined using the date of first initial contact with services and clinical records. Inclusion also required that participants had received minimal antipsychotic medication, which was defined as having received antipsychotic treatment for no longer than 4 weeks prior to the baseline visit, after a period of being either antipsychotic naïve or antipsychotic-free for at least 14 days. Participants were assessed at baseline within the first 2 weeks of antipsychotic medication initiation. Participants also were required to have the capacity to provide consent and the ability to read and write in English. Participants were excluded if they met modified Andreasen remission criteria[22], having mild or less scores on all of the following Structured Clinical Interview-Positive and Negative Syndrome Scale (SCI-PANSS)[23] items: delusions (P1), conceptual disorganisation (P2), hallucinatory behaviour (P3), blunted affect (N1), social withdrawal (N4), lack of spontaneity (N6), mannerisms/posturing (G5), unusual thought content (G9) on the day of assessment, as this would suggest that their symptoms were in remission. Participants were also required to show adherence to medication, as evidence by a Kemp Clinician Rating Scale (CRS)[24,25] of equal to or greater than 3 (*"Accepts only because compulsory, or very reluctant / requires persuasion, or questions the need for medication often*").

Participants were assessed within the first 14 days of starting antipsychotic medication at baseline, 2-weeks from baseline assessment (±7days of date) and 6-weeks from baseline assessment (±7 days of date), with the maximum cut-off for 6-week follow up being 56 days after baseline assessment (i.e. if an individual was assessed at the maximum follow-up periods at 2-week and 6-week visits; 8-weeks total). Participants were reimbursed for their time and expenses for participation in the study. Fourteen participants were withdrawn after providing consent, an additional 20 were withdrawn following baseline, and another 9 participants withdrawn following 2-week assessment. Participants were withdrawn if they were unable to attend the study visit, their symptoms were in remission (as per Andreasen

For peer review only

remission criteria[22]), or if they no longer wanted to take part in the study and requested to have their data removed (see Figure 1). 46 participants were eligible for inclusion in the analysis. All participants gave informed consent prior to enrolment. This study was approved by the Health and Social Care Research Ethics Committee A; REC: 17/NI/0209. All participants provided informed consent prior to participation.

[Figure 1; CONSORT]

Definitions for treatment response status

Treatment response groups were modelled through trajectory analyses using the *traj* command in STATA[26]. This tool estimates group-based trajectories over a specified time interval, clustering individuals who follow similar trajectories through a censored normal model. Akaike information criterion (AIC) and Bayesian information criterion (BIC) values were used to select the trajectory model with the lowest AIC and BIC values. Linear trajectories of up to five classes (1 to 5 trajectories) were assessed for eligibility. Rescaled PANSS scores[27] were calculated by subtracting 30 from total scores prior to producing estimates for percentage change at weeks 2 and 6 were used in the model. The results generated using this trajectory grouping were also compared to a more standard definition of treatment response which uses a >20% reduction in rescaled PANSS total scores from initial to final assessment[28,29]. Here patients not reaching a 20% reduction in rescaled PANSS total scores at the 6-week visits were categorised as non-responsive. These results are reported in the supplementary material.

Materials

Clinical and demographic measures

At baseline, participants completed the Kemp Clinician Rating Scale (CRS)[24,25], Mini-international neuropsychiatric interview (MINI)[30] (M-Psychotic Disorders; A-Major Depressive Episode; D-Manic/Hypomanic/Bipolar), Structured Clinical Interview- Positive and Negative Syndrome Scale (SCI-PANSS)[23], Clinician Rating Scale for Schizophrenia (CGI-SCH)[31] and provided demographic data. At each subsequent study visit the CRS, SCI-PANSS and CGI-SCH were repeated.

For peer review only

Neuropsychological assessment

Participants completed Version A of the Brief Assessment of Cognition in Schizophrenia (BACS)[32] at each study visit. The BACS was originally developed to assess cognitive functioning in schizophrenia, while also being an easily administrable and brief test battery[32]. The battery includes tasks pertaining to executive functions, working memory, motor/processing speed, verbal memory, verbal fluency, and attention cognitive domains. Version A includes the following tasks: i) list learning task (verbal memory); ii) digit sequencing task (working memory); iii) token motor task (motor speed); iv) category instances task (verbal fluency); v) symbol coding task (attention and speed of information processing) and vi) tower of London (executive function). All tasks on the BACS are scored such that higher scores represent better performance. Composite t and z scores for the BACS were generated using scores from published normative data[33].

Data analysis

All analyses were conducted in STATA 15/SE[34]. Wilcoxon signed rank tests were used to compare cognitive performance and symptom severity in the whole sample between visits (i.e. baseline assessment to 2-week, 2-week to 6-week, and baseline to 6-week) as not all symptom severity and cognitive variables were normally distributed. Baseline cognitive performance on the BACS was compared between trajectory groups using multivariable logistic regressions on the BACS composite and subscale scores. All models were adjusted for age, gender, and days from 1st psychotic symptom to baseline antipsychotic medication (i.e. duration of untreated psychosis; DUP). Results were then compared to groupings based on >20% reduction in rescaled PANSS total scores[27,28] from baseline assessment to 6-week follow-up (supplementary material Table S.1).

Finally, growth curve models were executed using the *xtmixed* command[35] to compare cognitive performance over time between trajectory groups to estimate any changes in cognitive performance over the study period (supplementary material Table S.2). These results were again compared to >20% PANSS reduction criteria for treatment response (supplementary material Table S.3).

Results

For peer review only - http://bmjopen.bmj.com/site/about/guidelines.xhtml

Table 1 reports the demographic descriptive of the whole sample included in analysis (N = 46) at baseline, with PANSS symptom severity scores and BACS performance for each study visit illustrated in Table 2. Data regarding antipsychotic medication was provided by all participants at baseline, all of which were treated with 2nd generation antipsychotics. At baseline 45 participants provided PANSS symptom severity ratings, with 41 providing at least one baseline measure of the BACS (Table 2). Between baseline and 2-week assessment the average follow-up was 18.19 days (SD = 6.6) and between 2-week and 6-week this was 26.69 days (SD = 9.6). Between baseline and 6-week visit, the study trial lasted 43.86 days (SD = 7.2).

Between study visits, a significant improvement in PANSS positive symptoms scores was observed in the whole sample between baseline and 2-week visits, 2-week and 6-week visits, as well as baseline and 6-week assessments (Table 2). A significant improvement in PANSS total scores was observed between baseline and 2-week and baseline and 6-week visits. No significant differences in symptom severity were observed between visits for negative symptoms (Table 2). In the whole sample, cognitive performance on the BACS verbal memory significantly improved between baseline and 2-week visits, 2-week and 6-week visits, as well as baseline and 6-week assessments (Table 2). Verbal fluency significantly improved between baseline and 2-week visits. Symbol coding, Tower of London, and overall (tscore and zscore) performance improved significantly between baseline and 2-week visits and baseline and 6-week visits (Table 2).

Table 1

Descriptive statistics of the whole sample demographics at consent (age) and baseline assessments

Variable	N	M	SD	Min	Max
Age (at consent)	46	27.30	8.17	18	50
Gender (*male*)	33 (71.74%)	-	-	-	-
Gender (*female*)	13	-	-	-	-

For peer review only

	(28.26%)				
Age of illness onset (years)	46	26.53	8.45	18	49
Duration from 1st Psychotic symptom (days) to baseline antipsychotic (DUP)	46	248.30	245.06	0	726
Duration from 1st contact with mental health services (days) to baseline antipsychotic	46	346.57	600.37	6	2358
Chlorpromazine equivalents (mg/day)	46	176.89	121.29	10	800
Number of hospitalisations	46	0.89	0.64	0	3
Years of education	42	13.62	2.82	5	20
CGI-SCH baseline score	56	Minimally ill = 1<br>Mildly ill = 4<br>Moderately ill = 12<br>Markedly ill = 15<br>Severely ill = 12<br>Among the most severely ill = 1	-	-	-
Antipsychotic medication	51	Amisulpride = 1<br>Aripiprazole = 19<br>Olanzapine = 16<br>Paliperidone = 1<br>Quetiapine = 4<br>Risperidone = 5	-	-	-

Note. *DUP* = Duration of untreated psychosis; *CGI-SCH* = Clinician Rating Scale for Schizophrenia.

For peer review only

Table 2

Mean symptom severity as rated by the PANSS and BACS performance for the whole sample for each study visit

	Baseline			2-week follow-up			6-week follow-up			Baseline vs 2-week	2 -week vs 6-week	Baseline vs 6-week
Variable	N	M	SD	N	M	SD	N	M	SD	Wilcoxon signed-rank	Wilcoxon signed-rank	Wilcoxon signed-rank
PANSS positive	45	11.93	4.77	36	9.17	5.36	38	7.26	5.13	Z = 2.76, *p* = .006*	Z = 2.67, *p* = .008*	Z = 4.50, *p* <.001*
PANSS negative	45	10.36	6.87	36	10.31	7.15	38	9.58	7.78	Z = 0.78, *p* = .435	Z = 0.62, *p* = .535	Z = 1.17, *p* = .242
PANSS general	45	19.40	8.57	36	16.64	10.60	38	15.74	10.11	Z = 3.21, *p* = .001*	Z = 2.48, *p* = .013*	Z = 0.64, *p* = .524
PANSS total	45	41.69	16.11	36	36.11	20.03	38	32.58	20.04	Z = 3.10, *p* = .002*	Z = 1.46, *p* = .144	Z = 3.35, *p* < .001*
BACS Verbal memory	41	37.83	14.02	32	41.16	13.50	36	44.56	15.02	Z = -3.14, *p* = .002*	Z = -3.15, *p* = .002*	Z = -3.88, *p* <.001*
BACS Digit sequencing	38	18.03	4.06	32	17.84	4.33	34	17.85	4.69	Z = -0.78, *p* = .433	Z = -0.40, *p* = .688	Z = 0.40, *p* = .687
BACS Verbal fluency	42	28.60	7.85	31	31.87	9.47	35	30.20	8.38	Z = -1.96, *p* = .050*	Z = 0.83, *p* = .405	Z = -1.62, *p* = .105
BACS Token motor	39	65.36	10.83	32	65.56	17.81	34	61.38	20.94	Z = -1.30, *p* = .193	Z = -0.55, *p* = .583	Z = -0.24, *p* = .812
BACS Symbol coding	39	40.15	13.13	32	45.69	12.56	32	47.25	11.96	Z = -2.25, *p* = .025*	Z = -1.07, *p* = .284	Z = -3.29, *p* = .001*

For peer review only

BACS ToL	37	14.84	4.48	29	17.38	3.29	32	16.28	4.13	Z = -3.24, *p* = .001*	Z = 1.45, *p* = .148	Z = -2.42, *p* = .016*
BACS t-score	33	26.67	11.98	28	34.14	11.68	30	30.87	14.95	Z = -3.79, *p* < .001*	Z = -0.29, *p* = .769	Z = -3.66, *p* <.001*
BACS z-score	33	-2.34	1.19	28	-1.59	1.17	30	-1.91	1.50	Z = -3.85, *p* <.001*	Z = -0.23, *p* = .820	Z = -3.67, *p* <.001*

Note. *PANSS* = Positive and Negative Symptom Scale; *BACS* =Brief Assessment of Cognition in Schizophrenia; *ToL* = Tower of London.
* = significant at $p = .05$ level.

For peer review only

Trajectories of symptom change

BIC and AIC values were generated for five classes of trajectory models (Table 3). Of these both indices indicate that the two-trajectory group is best fitted to the data. This model estimated 73.7% of the sampled population to be from one linear trajectory, with the remaining 26.3% in another.

Table 3
Selecting a trajectory model using BIC and AIC estimates

No. of classes	1	2	3	4	5
BIC	-522.21	-512.13	-517.87	-520.14	-525.88
AIC	-519.46	-506.64	-509.64	-509.17	-512.17
%. in each class	100%	73.7% ; 26.3%	73.7% ; 26.3%; 0%	60.7% ; 23.7%; 15.6%; 0%	60.7% ; 23.7%; 15.6%; 0%; 0%

Note. *BIC* = Bayesian Information Criteria; *AIC* = Akaike's Information Criteria.

The trajectories identified by the *traj* procedure are shown in Figure 2. The trajectories that emerged clearly represented responders versus non-responders. 39 participants (84.78%) of the sample were classified as antipsychotic treatment responsive and 7 as treatment non-responsive (15.22%). For responders, PANSS total score percentage change at 6 weeks was on average 32.89% (SD = 27.5) for symptom improvement. For non-responders this was -21.03% (SD = 16.1) indicating a decline in symptom improvement. Shape estimates and standard errors of antipsychotic response are shown in Table 4. Treatment responders significantly improved over the 6-week period. Descriptive statistics of clinical and demographic variables between both trajectory groups (non-responder; responder) are presented in the supplementary material (Table S.4.) In comparison to those responsive to antipsychotic medication, non-responders were on average older, had a longer duration of time from first contact with mental health services to baseline antipsychotic medication, had marginally more hospitalisations, attained more years of education, and were treated at higher chlorpromazine equivalents (supplementary material Table S.4).

For peer review only

[Figure 2]

Table 4

Parameter estimates and standard errors for both trajectories of antipsychotic response

Parameters	Trajectories	
	Non-responder (N = 7)	Responder (N = 39)
Intercept	2.54	-3.71
Linear change	0.10	-0.54
Standard error	0.06	0.09
T statistic	1.61, $p = .111$	-6.06, $p < .001$

Cognitive performance

There was a significant improvement in BACS verbal memory and symbol coding performance between baseline and 6-week assessments across the whole sample, with a significant improvement in Tower of London and BACS z and t composite scores between baseline and 2-week visits (Table 2). At baseline assessment, there was no difference in the BACS subscale or composite scores between antipsychotic responders and non-responders identified in the trajectory analysis (Table 5; Table 6). Growth curve models observed no significant change in cognitive performance over follow-up visits between trajectory groups (supplementary material Figure S.1, Table S.2). A similar pattern in results was observed when >20% PANSS reduction criteria was applied (supplementary material Figure S.2, Table S.3, Table S.5).

For peer review only

Table 5

Baseline cognitive performance for both trajectory groups

	Non-responder			Responder		
BACS measure	N	Mean	SD	N	Mean	SD
Verbal Memory	7	37.29	9.48	34	37.94	14.89
Digit Sequencing	6	20.17	5.38	32	17.63	3.74
Verbal Fluency	7	30.29	7.30	35	28.26	8.01
Token Motor	7	66.86	8.93	32	65.03	11.30
Symbol Coding	6	47.50	6.35	33	38.82	13.66
Tower of London	7	14.71	3.77	30	14.87	4.69
tscore composite	6	28.83	14.36	27	26.19	11.65
zscore composite	6	-2.12	1.41	27	-2.39	1.16

Note. *BACS* =Brief Assessment of Cognition in Schizophrenia.

Multivariable linear regression

Univariable and multivariable logistic regression models adjusting for age and gender and duration of untreated psychosis (DUP) found no significant associations between BACS performance at baseline and response trajectory over 6 weeks (Table 6), with no association of any demographic or clinical variables in multivariable models. This was also observed when utilising the >20% reduction in PANSS total criteria (supplementary material Table S.4).

For peer review only

Table 6

Results from univariable and multivariable logistic regression models for response status and baseline BACS performance

	Unadjusted					Adjusted for age, gender, and DUP				
BACS task	β	SE	95%CI	OR	P-value	β	SE	95%CI	OR	P-value
Verbal Memory	<0.01	0.03	-0.06 ; 0.06	1.00	.909	<-0.01	0.03	-0.07 ; 0.06	1.00	.918
Digit Sequencing	-0.17	0.12	-0.41 ; 0.07	0.84	.168	-0.18	0.13	-0.44 ; 0.07	0.83	.151
Verbal Fluency	-0.03	0.05	-0.14 ; 0.07	0.97	.530	-0.05	0.06	-0.17 ; 0.07	0.95	.417
Token Motor	-0.02	0.04	-0.09 ; 0.06	0.98	.683	-.0.02	0.05	-0.11 ; 0.08	0.99	.737
Symbol Coding	-0.06	0.04	-0.14 ; 0.02	0.94	.145	-0.07	0.05	-0.16 ; 0.02	0.93	.114
Tower of London	0.08	0.09	-0.18 ; 0.19	1.01	.935	-0.01	0.10	-0.20 ; 0.19	0.99	.947
t score composite	-0.02	0.04	-0.10 ; 0.06	0.98	.620	-0.02	0.04	-0.10 ; 0.06	0.98	.594
z score composite	-0.21	0.40	-0.99 ;0.58	0.81	.603	-0.23	0.41	-1.02 ;0.57	0.80	.573

Note. BACS = Brief Assessment of Cognition in Schizophrenia; *CIs* = confidence intervals; *DUP* = duration of untreated psychosis.

For peer review only

For peer review only - http://bmjopen.bmj.com/site/about/guidelines.xhtml

For peer review only

Discussion

This prospective study investigated the relationship between baseline cognitive performance and subsequent antipsychotic response over a 6-week treatment period. Across the whole sample, participants showed an overall reduction in symptom severity as well as an improvement in cognitive performance on the majority of BACS tasks. Trajectory analyses estimated two trajectory groups (73.7%, 26.3%) based on PANSS total % change from baseline; this was reflected as 84.78% of the sample being grouped as treatment responsive, and the remaining 15.22% as treatment non-responsive. Contrary to our hypothesis, baseline cognitive performance did not significantly differ between those identified as treatment responders or non-responders following 6 weeks of antipsychotic treatment. This finding remained the same when treatment response was defined as at least a 20% reduction in PANSS total scores, suggesting that there is no association between cognitive performance and antipsychotic response in first episode schizophrenia.

Across the 2-week and 6-week follow-up visits, an improvement in cognitive performance was observed for the whole sample on BACS measures of verbal memory, verbal fluency, symbol coding and Tower of London tasks, as well as the BACS composite scores. Most of these changes occurred between baseline and 2-week assessment (Table 2), with small decreases in performance on measures of verbal fluency, token motor and Tower of London tasks between 2-week and 6-week assessments, as well as for composite z and t scores. In contrast, there was a decline in performance in the token motor task across the follow-up period, and minimal changes in performance in the digit sequencing task.

The observed improvement in cognitive performance may reflect a beneficial outcome of antipsychotic treatment. 1st generation antipsychotics, introduced in the 1950s, target the positive symptoms observed in schizophrenia by acting as an antagonist at dopamine D2 receptors. Treatment with this group of antipsychotic drugs has been associated with motor and cognitive deficits in patients[36,37]. In contrast, 2nd generation antipsychotics are reported to have fewer extrapyramidal side effects[38], with these drugs also acting as an antagonist at the serotonin 5HT2A receptor, in addition to D2 dopamine receptors. Research suggests that in comparison to 1st generation, 2nd generation antipsychotics can provide some improvement in cognitive performance (e.g. clozapine[39]). Guilera et al (2009)[40] found in their meta-analysis of 18 randomised controlled trials that 2nd generation drugs provided a

For peer review only

slight improvement in performance for global cognition, as well as slight but significant improvements in measures of procedural learning, language and verbal comprehension, verbal learning and memory and visual learning and memory.

As the whole study sample was treated with 2nd generation antipsychotic drugs at baseline assessment (Amisulpride = 1, Aripiprazole = 19, Olanzapine = 16, Paliperidone = 1, Quetiapine = 4 and Risperidone = 5), it is possible that the improvement in cognitive performance observed in our sample may be a result of 2nd antipsychotic treatment effects, although 1st generation antipsychotic use could not be compared. However it has also been argued that improvements in cognitive performance over longitudinal designs may instead reflect practice effects (e.g. familiarity and procedural learning[41]), meaning that improvement in cognitive performance in our sample could also be attributable to practice effects between study visits. Lees et al[42] estimated the magnitude of these effects using both the MATRICS Consensus Cognitive Battery (MCCB)[43] and the Cog State Schizophrenia Battery[44], finding strong test-retest correlations between repeated baseline visits across cognitive batteries, with potential learning effects in social-emotional cognition. However, the authors also observed that participants may have failed to complete the initial baseline assessment due to difficulty in understanding the task, with the suggestion that future investigations using either battery would benefit from adopting initial practice sessions to reduce practice effects. Therefore an initial practice session with the BACS may have reduced the size of improvement observed in cognitive performance from baseline performance. Another way to determine the extent of practice effects in our sample would be to have a control group who is already stable on antipsychotic medication to see if similar outcomes are observed between groups.

Despite all of the sample being treated with 2nd generation antipsychotics, it is also possible that some anticholinergic effects, which differ between 2nd generation antipsychotic drugs[45], may have affected cognitive performance. Long term exposure to antipsychotic medications of high anticholinergic activity have been previously reported to impact cognitive performance in patient samples[46,47,48]. Using low and high anticholinergic activity criteria from a recent review comparing medication effects (from Stroup & Gray[49]; refer to Table 1, pg. 342), our sample had 44% (N = 20) treated with a high anticholinergic antipsychotic, meaning that the absence of significant differences between groups may have been a result of heterogeneity in medication effects. Therefore, future investigations should

For peer review only

consider the role of antipsychotic treatment effects on cognitive outcomes within schizophrenia.

Trajectory analyses identified two clearly defined trajectories of treatment response, both of which are consistent across both timepoints: one trajectory showing good response, and one of little to no response (Figure 2). Confidence intervals (Figure 2) show some overlap between trajectories in the first ~20 days since baseline assessment, with these becoming independent following this period, meaning that separation between trajectory groups was apparent at around 3 weeks. This supports the findings from Samara et al[6] who found poor/minimal response to antipsychotic treatment at 2-weeks to be predictive of future treatment non-response. In previous investigations using first episode samples, 4 or more trajectories have been identified[50,51]. However, both these investigations used longer periods of follow-up as well as raw un-adjusted PANSS scores in their analyses: as the minimum raw score of the PANSS is 30, it is recommended to rescale the scores by subtracting 30 from total scores prior to producing percentages and ratios[52]. Therefore building trajectory models using raw scores may not be appropriate to use as ratio operations (e.g. calculating proportions and percentages) require a natural zero point[52].

Growth curve models which were used to quantify change in cognitive performance between trajectory groups observed no significant changes in performance between visits. It is possible that this may be due to under sampled groups, as significant improvements for verbal memory, symbol coding, Tower of London and composite scores were observed in the whole sample. When comparing our findings to a >20% reduction in rescaled PANSS total scores criteria[24,25] there were no changes in the pattern of results to growth curve models or logistic regression outcomes. Using this criterion for treatment response resulted in a more even distribution of the total sample to groups (responder = 17 ; non-responder = 21), providing more power to comparative analyses. However despite this there was no change in the pattern of results, meaning that this criterion provided no added benefit to this analysis over trajectory-based groupings. The lack of significant difference in baseline cognition between those classified as treatment responders or non-responders after 6 weeks of treatment in our study contrasts previous research conducted which observed impaired cognitive performance in the poor response trajectory at week 4, with good performance at baseline being predictive of a good response trajectory at week 4[51]. Likewise, longitudinal research using the MCCB[43] with first-episode schizophrenia patients assessed at baseline

For peer review only

and at a 12-week follow up identified tasks of executive function and planning and reasoning ability as potential indices of antipsychotic response[53], with similar findings observed when cognitive performance is correlated with symptom severity measures[54].

Limitations

Previous investigations included sample sizes several magnitudes higher than in our study (Levine & Rabinowitz[51]; N = 491; Trampush et al[53]; N = 175) and it is likely that our sample size limited our ability to observe a significant relationship between cognitive performance and antipsychotic response. Using our sample's mean values for the BACS t composite score, a power calculation found that a total sample size of 31,304 samples would be required to detect a significant difference between trajectory groups at 90% power. When using the >20% PANSS reduction criteria this was N = 6,118, suggesting that both analyses were underpowered due to under sampling.

Another considerable limitation of the conclusions from this investigation is the expectation of detecting meaningful change in both clinical response to medication and cognition in such a short duration of follow-up. Previous longitudinal investigations into cognitive change have noted that even a period of 1 to 3 years may not be substantial to detect changes in cognitive performance[55] , questioning the additional analyses in this study comparing performance between baseline and 2-week and 6-week study visits. Likewise, Emsley et al's[56] investigation with 522 participants with first episode schizophrenia found 11.2% of their sample to not achieve clinical response (determined by a 20% improvement in PANSS total scores) until after 8 weeks, with the authors concluding that antipsychotic response is greatly varied and that longer investigations are needed to capture the large variability in clinical response[56]. Therefore it is also possible that there are participants within the sample who may have later responded to medication if the follow-up was at a longer duration, which may also partially support the lack of significant differences between groups in this study. Likewise, adopting secondary criteria for treatment response and non-response based off criteria from the TRIPP Working Group[57] would also help in seeing whether the groupings identified by trajectory analyses correspond to standardised guidelines, aiding in comparison between investigations.

Due to the issues with small sample sizes, it was not possible to adjust for additional variables which may be associated with cognitive performance. Negative symptoms have

For peer review only

routinely been associated with cognitive performance[58,59], including performance on the BACS[60]. Medication effects such as higher antipsychotic doses[61,62] and high anticholinergic antipsychotics[46,47,48] , have also been associated with deficits in cognitive performance. Future research should measure and adjust for these variables in order to determine the true association between cognition and treatment response without potential confounders.

It is also possible that premorbid histories of the sample may have resulted in a less consistent picture of cognitive performance between groups. For example, prior cannabis use, particularly during adolescence, has been found to improve cognitive performance on the BACS in comparison to those who haven't ever used cannabis[63]. In this investigation comparing performance on the BACS between patients with a schizophrenia diagnosis with and without adolescent cannabis use (ACU), those with ACU reported significantly higher composite scores, as well significant improvement in working memory and verbal memory tasks[63]. In our sample, 68% (N = 30) had previous experience of using cannabis, with the majority of this use occurring between ages 12-19 (N = 23). Therefore, it is possible that premorbid histories may have also blurred the cognitive differences between groups.

Conclusions

In this prospective cohort study, patients with a first episode diagnosis were assessed three times over a period of 6 weeks. Trajectory analyses using percentage change in PANSS total symptom scores identified two groups reflecting a good and poor response to antipsychotic medication. Baseline cognitive performance of these two groups did not predict response status at 6-weeks. This lack of discrimination between groups is potentially attributable to underpowered analyses as a result of small sample sizes but may also evidence that an association between cognition and treatment response is not observable in the first episode of schizophrenia. Overall this suggests that brief cognitive batteries for schizophrenia may not be a useful predictor of antipsychotic response in the first two-years of illness onset.

For peer review only

Contributorship:

J.H.M., A.E., B.D., R.J.D., O.D.H., L.K., C.C., A.S., R.O., S.Le., J.L., S.La., S.K., and E.Mik contributed to the design and implementation of the study. E.O., E.M., R.P., N.R., K.G., C.C., S.E.S., and K.V.S. aided in data collection. E.M. completed analyses and wrote the manuscript with the assistance of J.H.M. K.G., N.R., J.H.M., A.E., E.K., R.J.D., A.S., C.C., B.D., provided comments on the manuscript.

Funding statement:

STRATA is funded by a grant from the Medical Research Council (MRC) to JHM, grant reference MR/L011794. EM's PhD is funded by the MRC-doctoral training partnership studentship in Biomedical Sciences at King's College London. J.H.M., E.K., A.E., O.D.H. are part funded by the National Institute for Health Research (NIHR) Biomedical Research Centre at South London and Maudsley NHS Foundation Trust and King's College London. The views expressed are those of the authors and not necessarily those of the NHS, the MRC, the NIHR or the Department of Health.

Competing of Interests:

The remaining authors report no conflicts of interest.

Ethics approval:

This study was approved by the Health and Social Care Research Ethics Committee A; REC: 17/NI/0209.

Data sharing:

At the time of submission, the data governance frameworks are being put in place to make a fully anonymized version of the data available to the wider research community via the TranSMART data sharing platform: https://transmartfoundation.org/. To apply for access to the data, please contact J.H.M. at james.maccabe@kcl.ac.uk.

For peer review only

References

[1] McGlashan TH, Johannessen, JO. Early detection and intervention with schizophrenia: rationale. *Schizophrenia bulletin* 1996;22(2),201-222.

[2] Birchwood M, McGorry P, Jackson H. Early intervention in schizophrenia. *The British Journal of Psychiatry* 1997;170(1),2-5.

[3] Bond GR, Drake RE, Luciano A. Employment and educational outcomes in early intervention programmes for early psychosis: a systematic review. *Epidemiology and psychiatric sciences* 2015; 24(5),446-457.

[4] Bozzatello P, Bellino S, Rocca P. Predictive factors of treatment resistance in first episode of psychosis: A systematic review. *Frontiers in psychiatry* 2019;10, 67.

[5] Carbon, M, Correll CU. Clinical predictors of therapeutic response to antipsychotics in schizophrenia. *Dialogues in clinical neuroscience* 2014;16(4), 505.

[6] Samara, MT, Leucht C, Leeflang M, et al. Early improvement as a predictor of later response to antipsychotics in schizophrenia: a diagnostic test review. *American journal of psychiatry* 2015;172(7),617-629.

[7] Griffiths K, Millgate E, Egerton A, et al. Demographic and clinical variables associated with response to clozapine in schizophrenia: a systematic review and meta-analysis. *Psychological Medicine* 2021;1-11.

[8] Yoshimura B, Yada Y, So R, et al. The critical treatment window of clozapine in treatment-resistant schizophrenia: secondary analysis of an observational study. *Psychiatry research* 2017;250,65-70.

[9] Kennedy JL, Altar CA, Taylor DL, et al. The social and economic burden of treatment-resistant schizophrenia: a systematic literature review. *International clinical psychopharmacology* 2014;1;29(2):63-76.

[10] McCrone P, Dhanasiri S, Patel A, et al. The cost of mental health care in England to 2026. *The King's Fund* 2008;1-65.

[11] National Institute for Health and Care Excellence. Psychosis and schizophrenia in adults: prevention and management. *NICE*; 2014.

[12] Tsiachristas A, Thomas T, Leal J, et al. Economic impact of early intervention in psychosis services: results from a longitudinal retrospective controlled study in England. *BMJ open* 2016;1;6(10):e012611.

[13] Reichenberg A, Caspi A, Harrington H, et al. Static and dynamic cognitive deficits in childhood preceding adult schizophrenia: a 30-year study. *American Journal of Psychiatry* 2010;167(2),160-169.

[14] MacCabe JH, Wicks S, Löfving S, et al. Decline in cognitive performance between ages 13 and 18 years and the risk for psychosis in adulthood: a Swedish longitudinal cohort study in males. *Jama Psychiatry* 2013;70(3),261-270.

[15] Green MF, Kern RS, Heaton RK. Longitudinal studies of cognition and functional outcome in schizophrenia: implications for MATRICS. *Schizophrenia research* 2004;72(1),41-51.

[16] Carrión RE, Goldberg TE, McLaughlin D, et al. Impact of neurocognition on social and role functioning in individuals at clinical high risk for psychosis. *American Journal of Psychiatry* 2011;168(8),806-813.

[17] Horan WP, Green MF, DeGroot M, et al. Social cognition in schizophrenia, part 2: 12-month stability and prediction of functional outcome in first-episode patients. *Schizophrenia bulletin* 2012;38(4),865-872.

[18] Millgate E, Hide O, Lawrie SM, et al. Neuropsychological differences between treatment-resistant and treatment-responsive schizophrenia: a meta-analysis. *Psychological medicine* 2022;52(1),1-13.

[19] American Psychological Association. Distinguishing between screening and assessment for mental and behavioral health problems. 2014.

[20] Roebuck-Spencer TM, Glen T, Puente AE, et al. Cognitive screening tests versus comprehensive neuropsychological test batteries: a national academy of neuropsychology education paper. *Archives of Clinical Neuropsychology* 2017 Jun 1;32(4):491-8.

[21] Hurford IM, Marder SR, Keefe RS, et al. A brief cognitive assessment tool for schizophrenia: construction of a tool for clinicians. *Schizophrenia Bulletin* 2011 May 1;37(3):538-45.

[22] Andreasen NC, Carpenter JrWT, Kane JM, et al. Remission in schizophrenia: proposed criteria and rationale for consensus. *American Journal of Psychiatry* 2005;162(3),441-449.

[23] Kay SR, Fiszbein A, Opler LA. The positive and negative syndrome scale (PANSS) for schizophrenia. *Schizophrenia bulletin* 1987;13(2),261-276.

[24] Kemp R, Hayward P, Applewhaite G, et al. Compliance therapy in psychotic patients: randomised controlled trial. *Bmj* 1996;312(7027),345-349.

[25] Kemp R, Kirov G, Everitt B, et al. Randomised controlled trial of compliance therapy: 18-month follow-up. *The British Journal of Psychiatry* 1998;172(5),413-419.

For peer review only

[26] Jones BL, Nagin DS. A note on a Stata plugin for estimating group-based trajectory models. *Sociological Methods & Research* 2013;42(4),608-613.

[27] Leucht S, Kissling W, Davis JM. The PANSS should be rescaled. *Schizophrenia bulletin* 2010;36(3),461-462.

[28] Leucht S, Kane JM, Kissling W, et al. What does the PANSS mean?. *Schizophrenia research* 2005;79(2-3),231-238.

[29] Leucht S, Davis JM, Engel RR, et al. Definitions of response and remission in schizophrenia: recommendations for their use and their presentation. *Acta Psychiatrica Scandinavica* 2009;119,7-14.

[30] Sheehan DV, Lecrubier Y, Sheehan KH, et al. The Mini-International Neuropsychiatric Interview (MINI): the development and validation of a structured diagnostic psychiatric interview for DSM-IV and ICD-10. *The Journal of clinical psychiatry* 1998.

[31] Guy W. ECDEU assessment manual for psychopharmacology. *US Department of Health, Education, and Welfare, Public Health Service, Alcohol, Drug Abuse, and Mental Health Administration, National Institute of Mental Health, Psychopharmacology Research Branch, Division of Extramural Research Programs* 1976.

[32] Keefe RS, Goldberg TE, Harvey PD, et al. The Brief Assessment of Cognition in Schizophrenia: reliability, sensitivity, and comparison with a standard neurocognitive battery. *Schizophrenia research* 2004;68(2-3),283-297.

[33] Keefe RS, Harvey PD, Goldberg TE, et al. Norms and standardization of the Brief Assessment of Cognition in Schizophrenia (BACS). *Schizophrenia research* 2008;102(1-3),108-115.

[34] StataCorp. Stata Statistical Software: Release 15. 2017. College Station, TX: StataCorp LLC.

[35] StataCorp, LP. Stata multilevel mixed-effects reference manual. 2013. College Station, TX: StataCorp LP, 9(10).

[36] Kapur S, Zipursky R, Jones C, et al. Relationship between dopamine D2 occupancy, clinical response, and side effects: a double-blind PET study of first-episode schizophrenia. *American Journal of Psychiatry* 2000;157(4),514-520.

[37] Reilly JL, Harris MS, Keshavan MS, et al. Adverse effects of risperidone on spatial working memory in first-episode schizophrenia. *Archives of general psychiatry* 2006;63(11),1189-1197.

[38] Hill SK, Bishop JR, Palumbo D, et al. Effect of second-generation antipsychotics on cognition: current issues and future challenges. *Expert review of neurotherapeutics* 2010;10(1),43-57.

For peer review only

[39] Kane J, Honigfeld G, Singer J, et al. Clozapine for the treatment-resistant schizophrenic: a double-blind comparison with chlorpromazine. *Archives of general psychiatry* 1988;45(9),789-796.

[40] Guilera G, Pino O, Gómez-Benito J, et al. Antipsychotic effects on cognition in schizophrenia: a meta-analysis of randomised controlled trials. *The European Journal of Psychiatry* 2009;23(2),77-89.

[41] Goldberg TE, Goldman RS, Burdick KE, et al. Cognitive improvement after treatment with second-generation antipsychotic medications in first-episode schizophrenia: is it a practice effect?. *Archives of general psychiatry* 2007;64(10),1115-1122.

[42] Lees J, Applegate E, Emsley R, et al. Calibration and cross-validation of MCCB and CogState in schizophrenia. *Psychopharmacology* 2015;232(21),3873-3882.

[43] Nuechterlein KH, Green MF, Kern RS, et al. The MATRICS Consensus Cognitive Battery, part 1: test selection, reliability, and validity. *American Journal of Psychiatry* 2008;165(2),203-213.

[44] Maruff P, Thomas E, Cysique L, et al. Validity of the CogState brief battery: relationship to standardized tests and sensitivity to cognitive impairment in mild traumatic brain injury, schizophrenia, and AIDS dementia complex. *Archives of Clinical Neuropsychology* 2009;24(2),165-178.

[45] Lieberman JA. Managing anticholinergic side effects. *Primary care companion to the Journal of clinical psychiatry* 2004;6(suppl2),20.

[46] Strauss ME, Reynolds KS, Jayaram G, et al. Effects of anticholinergic medication on memory in schizophrenia. *Schizophrenia Research* 1990;3(2),127-129.

[47] Campbell N, Boustani M, Limbil T, et al. The cognitive impact of anticholinergics: a clinical review. *Clinical interventions in aging* 2009;4,225.

[48] Joshi YB, Thomas ML, Braff DL, et al. Anticholinergic Medication Burden–Associated Cognitive Impairment in Schizophrenia. *American Journal of Psychiatry* 2021;appi-ajp.

[49] Stroup TS, Gray N. Management of common adverse effects of antipsychotic medications. *World Psychiatry* 2018;17(3),341-356.

[50] Abdin E, Chong SA, Vaingankar JA, et al. Trajectories of positive, negative and general psychopathology symptoms in first episode psychosis and their relationship with functioning over a 2-year follow-up period. *PloS one* 2017;12(11),e0187141.

[51] Levine SZ, Rabinowitz J. Trajectories and antecedents of treatment response over time in early-episode psychosis. *Schizophrenia bulletin* 2010;36(3),624-632.

For peer review only

[52] Obermeier M, Mayr A, Schennach-Wolff R, et al. Should the PANSS be rescaled?. *Schizophrenia bulletin* 2010;36(3),455-460.

[53] Trampush JW, Lencz T, DeRosse P, et al. Relationship of cognition to clinical response in first-episode schizophrenia spectrum disorders. *Schizophrenia bulletin* 2015;41(6),1237-1247.

[54] Bilder RM, Goldman RS, Robinson D, et al. Neuropsychology of first-episode schizophrenia: initial characterization and clinical correlates. *American Journal of Psychiatry* 2000;157(4),549-559.

[55] Zanelli J, Mollon J, Sandin S, et al. Cognitive change in schizophrenia and other psychoses in the decade following the first episode. *American Journal of Psychiatry* 2019 Oct 1;176(10):811-9.

[56] Emsley R, Rabinowitz J, Medori R. Time course for antipsychotic treatment response in first-episode schizophrenia. *American Journal of Psychiatry* 2006 Apr;163(4):743-5.

[57] Howes OD, McCutcheon R, Agid O, et al. Treatment-resistant schizophrenia: treatment response and resistance in psychosis (TRRIP) working group consensus guidelines on diagnosis and terminology. *American Journal of Psychiatry* 2017 Mar 1;174(3):216-29.

[58] Addington J, Addington D, Maticka-Tyndale E. Cognitive functioning and positive and negative symptoms in schizophrenia. *Schizophrenia research* 1991 Sep 1;5(2):123-34.

[59] Hoffman RE, Rakfeldt J. Cognition, negative symptoms, and diagnosis: a comparison of schizophrenic, bipolar, and control samples. *Neurosciences* 1997;9(1):81-9.

[60] Tanaka T, Tomotake M, Ueoka Y, et al. Clinical correlates associated with cognitive dysfunction in people with schizophrenia. *Psychiatry and clinical neurosciences* 2012 Oct;66(6):491-8.

[61] Elie D, Poirier M, Chianetta JM, et al. Cognitive effects of antipsychotic dosage and polypharmacy: a study with the BACS in patients with schizophrenia and schizoaffective disorder. *Journal of Psychopharmacology* 2010 Jul;24(7):1037-44.

[62] Ballesteros A, Sánchez-Torres AM, López-Ilundain JM, et al. Is cognitive impairment associated with antipsychotic dose and anticholinergic equivalent loads in first-episode psychosis?. *Psychological medicine* 2018 Oct;48(13):2247-56.

[63] Hanna RC, Shalvoy A, Cullum CM, et al. Cognitive function in individuals with psychosis: moderation by adolescent cannabis use. Schizophrenia bulletin 2016;42(6),1496-1503.

For peer review only

Figure legends

Figure 1. A CONSORT based flowchart illustrating the number of participants and exclusions at each stage of the study trial.

Figure 2. Trajectory model of total PANSS score percentage change from baseline modelled over days since baseline assessment. The dotted linear trajectory reflects treatment non-responders and the complete line treatment responders. The grey dotted lines around each trajectory reflect the confidence intervals for trajectory each group. Percentages reflect the estimated amount of the sampled population included in each trajectory.

For peer review only

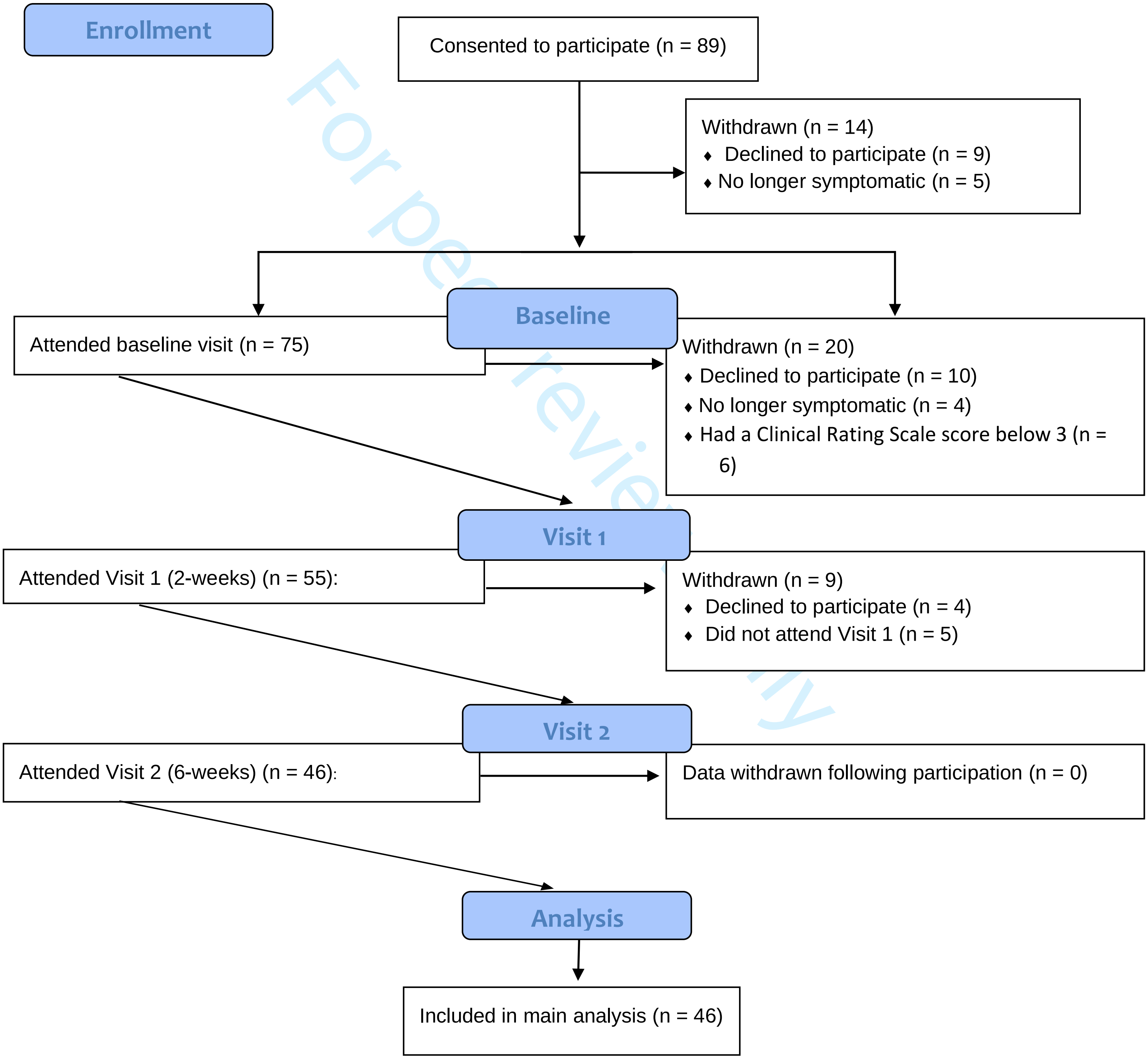

For peer review only

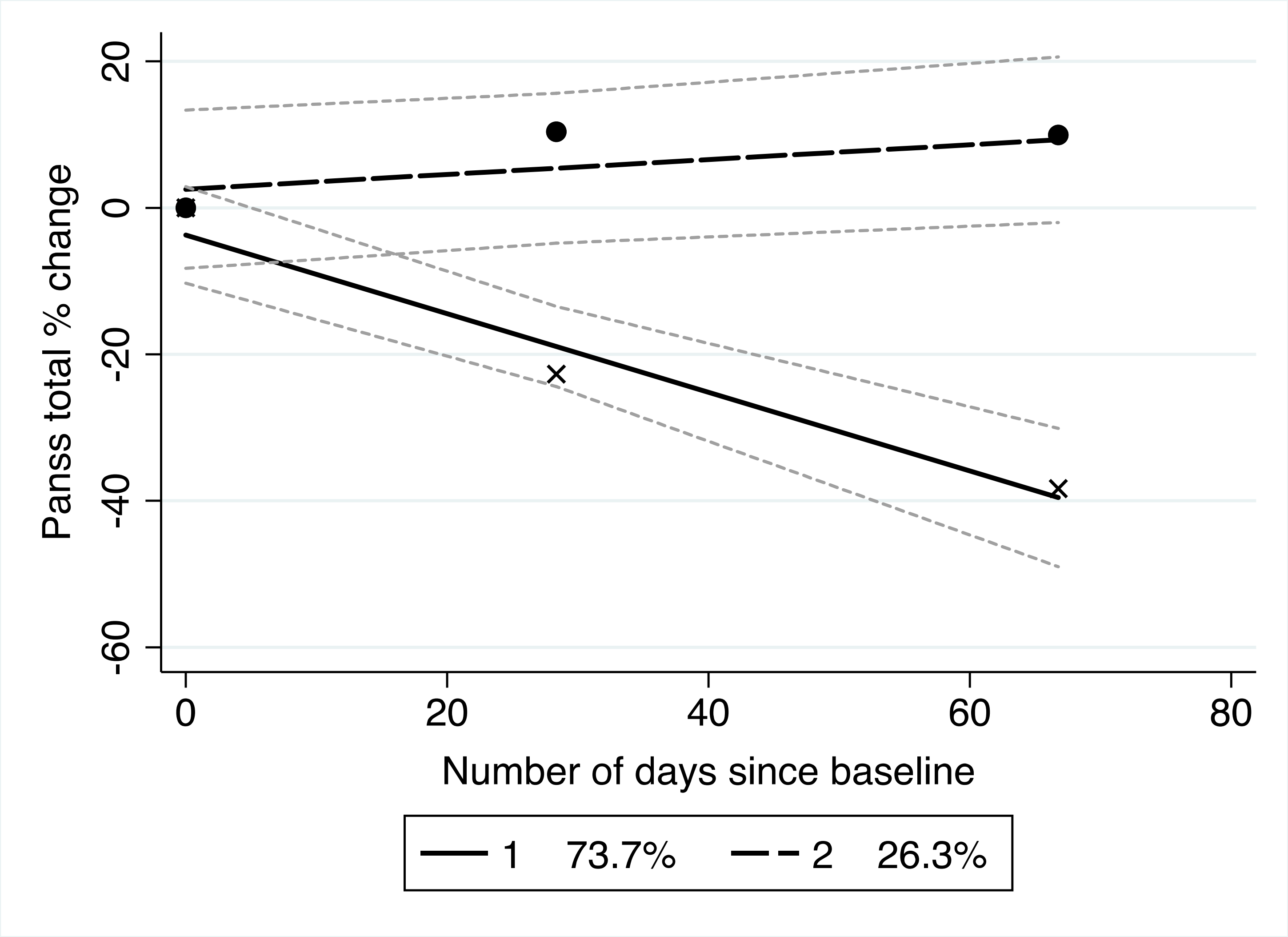
Panss total % change
20
0
-20
-40
-60
0
20
40
60
80
Number of days since baseline
1 73.7%
2 26.3%

Supplementary material

Table S.1

Results from univariable and multivariable logistic regression models for response status using PANSS total >20% reduction criteria and baseline BACS performance

	Unadjusted					Adjusted for age, gender and DUP				
BACS task	β	SE	95%CI	OR	P-value	β	SE	95%CI	OR	P-value
Verbal Memory	-0.01	0.03	-0.07 ; 0.06	0.99	.819	<-0.01	0.04	-0.08 ; 0.07	1.00	.920
Digit Sequencing	-0.10	0.10	-0.29 ; 0.09	0.90	.297	-0.06	0.11	-0.27 ; 0.16	0.95	.604
Verbal Fluency	0.04	0.05	-0.06 ; 0.14	1.04	.403	0.07	0.06	-0.05 ; 0.18	1.07	.259
Token Motor	0.03	0.04	-0.04 ; 0.10	1.03	.432	0.06	0.05	-0.03 ; 0.15	1.06	.218
Symbol Coding	-0.04	0.03	-0.11 ; 0.02	0.96	.206	-0.05	0.04	-0.12 ; 0.03	0.96	.237
Tower of London	0.07	0.09	-0.11 ; 0.24	1.07	.445	0.07	0.09	-0.12 ; 0.25	1.07	.465
t score composite	<0.01	0.03	-0.06 ; 0.07	1.00	.924	0.01	0.03	-0.06 ; 0.07	1.00	.892
z score composite	0.02	0.33	-0.63 ; 0.66	1.02	.960	0.03	0.34	-0.63 ; 0.69	1.03	.931

Note. BACS = Brief Assessment of Cognition in Schizophrenia; *DUP* = duration of untreated psychosis; *CIs* = confidence intervals.

For peer review only

Table S.2

Results from unadjusted and adjusted growth curve models comparing trajectory groups on BACS performance across all visits

	Unadjusted				Adjusted for age, gender and DUP			
BACS task	β	SE	95%CI	P-value	β	SE	95%CI	P-value
Verbal Memory	-2.15	45.79	-13.<br>49 ; 9.19	.710	-2.67	5.88	-14.20 ;8.86	.650
Digit Sequencing	-2.25	1.63	-5.45 ; 1<br>0.95	.167	-2.03	1.62	-5.19 ; 1.14	.210
Verbal Fluency	-0.90	3.29	-7.36 ; 5.55	.784	-1.31	2.99	-7.17 ; 4.54	.660
Token Motor	-2.00	6.39	-14.52 ;<br>10.53	.755	-0.45	5.89	11.99 ; 11.10	.939
Symbol Coding	-3.81	5.56	-14.71 ; 7.08	.493	-4.25	5.42	-14.87 ; 6.37	.433
Tower of London	0.18	1.56	-2.87 ; 3.24	.906	0.17	1.57	-2.90 ; 3.25	.912
t score composite	-2.49	5.70	-13.66 ; 8.68	.662	-2.43	5.78	-13.76 ; 8.89	.674
z score composite	-0.25	0.57	-1.37 ; 0.87	.658	-0.25	0.58	-1.38 ; 0.88	.665

Note. *BACS* = Brief Assessment of Cognition in Schizophrenia; *DUP* = duration of untreated psychosis; *CIs* = confidence intervals

For peer review only

Table S.3

Results from unadjusted and adjusted growth curve models comparing PANSS >20% reduction groups on BACS performance across all visits

	Unadjusted				Adjusted for age, gender and DUP			
BACS task	β	SE	95%CI	P-value	β	SE	95%CI	P-value
Verbal Memory	-4.18	4.21	-12.44 ; 4.08	.321	3.27	4.34	-11.78 ; 5.23	.451
Digit Sequencing	-2.36	1.35	-5.01 ; 0.29	.081	-1.78	1.34	-4.41 ; 0.86	.186
Verbal Fluency	-0.97	2.55	-5.97 ; 4.02	.703	-0.54	2.49	-5.42 ; 4.35	.830
Token Motor	1.81	5.52	-9.01 ; 12.62	.744	5.60	5.07	-4.33 ; 15.53	.269
Symbol Coding	-4.23	3.92	-11.92 ; 3.46	.281	-4.13	4.03	-12.03 ; 3.78	.306
Tower of London	-0.77	1.25	-3.22 ; 1.68	.538	-0.56	1.30	-3.10 ; 1.98	.667
t score composite	-2.39	4.75	-11.71 ; 6.93	.615	-1.56	4.84	-11.03 ; 7.92	.747
z score composite	-0.25	0.48	-1.18 ; 0.68	.600	-0.17	0.48	-1.12 ; 0.78	.728

Note. *BACS* = Brief Assessment of Cognition in Schizophrenia; *DUP* = duration of untreated psychosis; *CIs* = confidence intervals

For peer review only

Table S.4

Descriptive statistics of clinical and demographic variables for each trajectory group at baseline assessments

	Non-responder			Responder		
Variable	N	M	SD	N	M	SD
Age (at consent)	6	29.57	6.70	39	26.90	8.41
Gender (*male*)	6	-	-	27	-	-
Gender (*female*)	1	-	-	12	-	-
Age of illness onset (years)	7	27.54	8.09	39	26.34	8.60
Duration from 1st Psychotic symptom (days) to baseline antipsychotic (DUP)	7	177.09	207.93	39	261.08	251.38
Duration from 1st contact with mental health services (days) to baseline antipsychotic	7	461.89	801.88	39	325.88	567.82
Chlorpromazine equivalents (mg/day)	7	271.43	249.76	39	159.92	75.02
Number of hospitalisations	7	1.00	1.00	39	0.87	0.57
Years of education	6	17.00	2.53	36	13.06	2.47
PANSS positive	7	11.14	6.67	38	12.08	4.44
PANSS negative	7	12.57	5.77	38	9.95	7.04
PANSS general	7	19.57	8.70	38	19.37	8.66
PANSS total	7	43.29	13.52	38	41.39	16.68

Note. PANSS = Positive and Negative Symptom Scale.

For peer review only

Table S.5

Baseline cognitive performance for both groups using >20% PANSS reduction criteria

	Non-responder			Responder		
BACS measure	N	Mean	SD	N	Mean	SD
Verbal Memory	13	37.54	8.27	20	36.65	12.73
Digit Sequencing	12	19.25	4.20	19	17.68	3.97
Verbal Fluency	14	27.36	6.44	20	29.45	7.75
Token Motor	13	63.23	10.94	18	66.39	11.24
Symbol Coding	12	44.83	9.47	20	39.35	12.75
Tower of London	12	14.17	5.11	17	15.41	3.79
tscore composite	10	26.30	13.35	17	26.77	12.28
zscore composite	10	-2.36	1.31	17	-2.34	1.22

Note. *BACS* = Brief Assessment of Cognition in Schizophrenia; *PANSS* = Positive and Negative Symptom Scale.

For peer review only

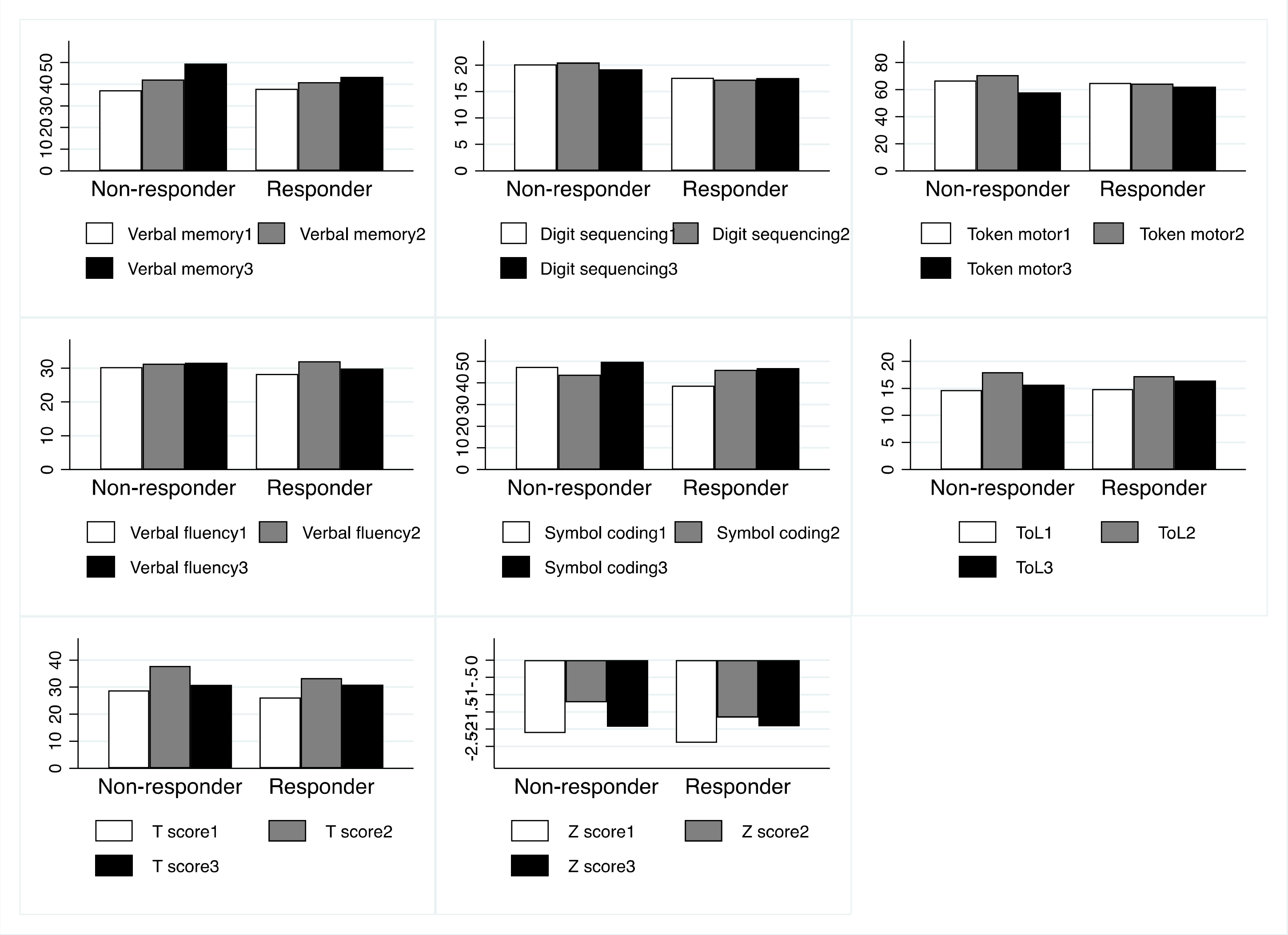

Figure S.1

Bar graphs comparing mean performance on BACS measures between trajectory groups (non-responder vs. responder) at each visit (*white* = baseline, *grey* = 2-week, *black* = 6-week)

Note. *BACS* = Brief Assessment of Cognition in Schizophrenia; *ToL* = Tower of London.

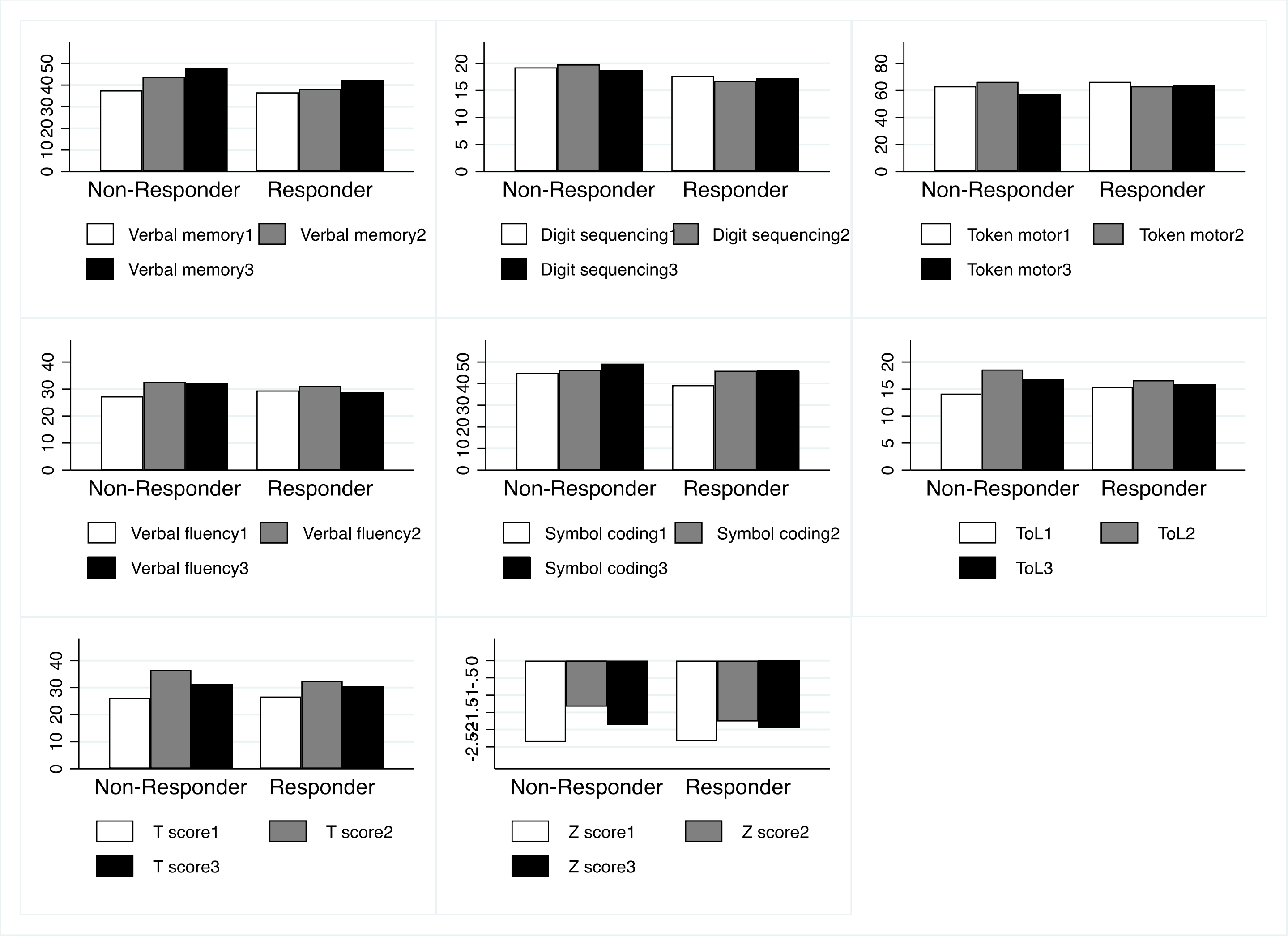

Figure S.2

Bar graphs comparing mean performance on BACS measures using >20% PANSS reduction criteria (non-responder vs. responder) at each visit (*white* = baseline, *grey* = 2-week, *black* = 6-week)

Note. *BACS* = Brief Assessment of Cognition in Schizophrenia; *ToL* = Tower of London.

For peer review only

STROBE Statement—checklist of items that should be included in reports of observational studies

	Item No	Recommendation	Noted on pg.
**Title and abstract**	1	(*a*) Indicate the study's design with a commonly used term in the title or the abstract	1,2,3
		(*b*) Provide in the abstract an informative and balanced summary of what was done and what was found	1,2,3
**Introduction**			
Background/rationale	2	Explain the scientific background and rationale for the investigation being reported	5,6
Objectives	3	State specific objectives, including any prespecified hypotheses	6
**Methods**			
Study design	4	Present key elements of study design early in the paper	7
Setting	5	Describe the setting, locations, and relevant dates, including periods of recruitment, exposure, follow-up, and data collection	7
Participants	6	(*a*) *Cohort study*—Give the eligibility criteria, and the sources and methods of selection of participants. Describe methods of follow-up<br>*Case-control study*—Give the eligibility criteria, and the sources and methods of case ascertainment and control selection. Give the rationale for the choice of cases and controls<br>*Cross-sectional study*—Give the eligibility criteria, and the sources and methods of selection of participants	7,8
		(*b*) *Cohort study*—For matched studies, give matching criteria and number of exposed and unexposed<br>*Case-control study*—For matched studies, give matching criteria and the number of controls per case	N/A
Variables	7	Clearly define all outcomes, exposures, predictors, potential confounders, and effect modifiers. Give diagnostic criteria, if applicable	7,8
Data sources/ measurement	8*	For each variable of interest, give sources of data and details of methods of assessment (measurement). Describe comparability of assessment methods if there is more than one group	7,8
Bias	9	Describe any efforts to address potential sources of bias	9
Study size	10	Explain how the study size was arrived at	N/A
Quantitative variables	11	Explain how quantitative variables were handled in the analyses. If applicable, describe which groupings were chosen and why	8,9
Statistical methods	12	(*a*) Describe all statistical methods, including those used to control for confounding	8,9
		(*b*) Describe any methods used to examine subgroups and interactions	8,9
		(*c*) Explain how missing data were addressed	N/A
		(*d*) *Cohort study*—If applicable, explain how loss to follow-up was addressed<br>*Case-control study*—If applicable, explain how matching of cases and controls was addressed<br>*Cross-sectional study*—If applicable, describe analytical methods taking account of sampling strategy	N/A
		(*e*) Describe any sensitivity analyses	8,9

For peer review only

Continued on next page

For peer review only

**Results**			
Participants	13*	(a) Report numbers of individuals at each stage of study—eg numbers potentially eligible, examined for eligibility, confirmed eligible, included in the study, completing follow-up, and analysed	6,7
		(b) Give reasons for non-participation at each stage	7,8
		(c) Consider use of a flow diagram	N/A
Descriptive data	14*	(a) Give characteristics of study participants (eg demographic, clinical, social) and information on exposures and potential confounders	9,10
		(b) Indicate number of participants with missing data for each variable of interest	N/A
		(c) *Cohort study*—Summarise follow-up time (eg, average and total amount)	N/A
Outcome data	15*	*Cohort study*—Report numbers of outcome events or summary measures over time	10,11,supplem.
		Case-control study—Report numbers in each exposure category, or summary measures of exposure	N/A
		Cross-sectional study—Report numbers of outcome events or summary measures	N/A
Main results	16	(*a*) Give unadjusted estimates and, if applicable, confounder-adjusted estimates and their precision (eg, 95% confidence interval). Make clear which confounders were adjusted for and why they were included	17, supplem.
		(*b*) Report category boundaries when continuous variables were categorized	N/A
		(*c*) If relevant, consider translating estimates of relative risk into absolute risk for a meaningful time period	17, supplem.
Other analyses	17	Report other analyses done—eg analyses of subgroups and interactions, and sensitivity analyses	13-15, Supplem.
**Discussion**			
Key results	18	Summarise key results with reference to study objectives	20,21
Limitations	19	Discuss limitations of the study, taking into account sources of potential bias or imprecision. Discuss both direction and magnitude of any potential bias	23,24
Interpretation	20	Give a cautious overall interpretation of results considering objectives, limitations, multiplicity of analyses, results from similar studies, and other relevant evidence	20,21,23
Generalisability	21	Discuss the generalisability (external validity) of the study results	20,21,23
**Other information**			
Funding	22	Give the source of funding and the role of the funders for the present study and, if applicable, for the original study on which the present article is based	25

*Give information separately for cases and controls in case-control studies and, if applicable, for exposed and unexposed groups in cohort and cross-sectional studies.

Note: An Explanation and Elaboration article discusses each checklist item and gives methodological background and published examples of transparent reporting. The STROBE checklist is best used in conjunction with this article (freely available on the Web sites of PLoS Medicine at http://www.plosmedicine.org/, Annals of Internal Medicine at http://www.annals.org/, and Epidemiology at http://www.epidem.com/). Information on the STROBE Initiative is available at www.strobe-statement.org.

For peer review only
